# Effective Behavior Change Techniques in Digital Health Interventions for the Prevention or Management of Noncommunicable Diseases: An Umbrella Review

**DOI:** 10.1093/abm/kaad041

**Published:** 2023-08-25

**Authors:** Jacqueline Louise Mair, Alicia Salamanca-Sanabria, Mareike Augsburger, Bea Franziska Frese, Stefanie Abend, Robert Jakob, Tobias Kowatsch, Severin Haug

**Affiliations:** Future Health Technologies, Singapore-ETH Centre, Campus for Research Excellence And Technological Enterprise (CREATE), Singapore; Saw Swee Hock School of Public Health, National University of Singapore, Singapore; Centre for Digital Health Interventions, Department of Management, Technology, and Economics, ETH Zurich, Zurich, Switzerland; Future Health Technologies, Singapore-ETH Centre, Campus for Research Excellence And Technological Enterprise (CREATE), Singapore; Swiss Research Institute for Public Health and Addiction, University of Zurich, Zurich, Switzerland; Klenico Health AG, Zurich, Switzerland; Future Health Technologies, Singapore-ETH Centre, Campus for Research Excellence And Technological Enterprise (CREATE), Singapore; Centre for Digital Health Interventions, Institute of Technology Management, University of St.Gallen, St.Gallen, Switzerland; Swiss Research Institute for Public Health and Addiction, University of Zurich, Zurich, Switzerland; Centre for Digital Health Interventions, Department of Management, Technology, and Economics, ETH Zurich, Zurich, Switzerland; Institute for Implementation Science in Health Care, University of Zurich, Zurich, Switzerland; School of Medicine, University of St.Gallen, St.Gallen, Switzerland; Centre for Digital Health Interventions, Department of Management, Technology, and Economics, ETH Zurich, Zurich, Switzerland; Future Health Technologies, Singapore-ETH Centre, Campus for Research Excellence And Technological Enterprise (CREATE), Singapore; Swiss Research Institute for Public Health and Addiction, University of Zurich, Zurich, Switzerland

**Keywords:** Digital health, Intervention components, Behavior change technique, Lifestyle behaviors, mHealth, eHealth

## Abstract

**Background:**

Despite an abundance of digital health interventions (DHIs) targeting the prevention and management of noncommunicable diseases (NCDs), it is unclear what specific components make a DHI effective.

**Purpose:**

This narrative umbrella review aimed to identify the most effective behavior change techniques (BCTs) in DHIs that address the prevention or management of NCDs.

**Methods:**

Five electronic databases were searched for articles published in English between January 2007 and December 2022. Studies were included if they were systematic reviews or meta-analyses of DHIs targeting the modification of one or more NCD-related risk factors in adults. BCTs were coded using the Behavior Change Technique Taxonomy v1. Study quality was assessed using AMSTAR 2.

**Results:**

Eighty-five articles, spanning 12 health domains and comprising over 865,000 individual participants, were included in the review. We found evidence that DHIs are effective in improving health outcomes for patients with cardiovascular disease, cancer, type 2 diabetes, and asthma, and health-related behaviors including physical activity, sedentary behavior, diet, weight management, medication adherence, and abstinence from substance use. There was strong evidence to suggest that credible source, social support, prompts and cues, graded tasks, goals and planning, feedback and monitoring, human coaching and personalization components increase the effectiveness of DHIs targeting the prevention and management of NCDs.

**Conclusions:**

This review identifies the most common and effective BCTs used in DHIs, which warrant prioritization for integration into future interventions. These findings are critical for the future development and upscaling of DHIs and should inform best practice guidelines.

## Teaser

Digital health interventions that incorporate social support, prompts or cues, a credible source, and self-regulatory behaviour change techniques are more effective in changing lifestyle behaviours and health outcomes.

## Introduction

Noncommunicable diseases (NCDs) such as cardiovascular diseases (CVDs), cancers, respiratory diseases, and diabetes are the leading causes of death and disability worldwide [1]. Furthermore, it is well established that many NCDs also increase the risk for mental disorders [2–5], and that risk factors for NCDs such as tobacco use, harmful alcohol, unhealthy diet, and physical inactivity commonly cluster in people with mental disorders [6]. The combined health, societal, and economic burden of NCDs and mental disorders is tremendous. Projections estimate the global cumulative economic cost of NCDs between 2010–2030 to reach US$47 trillion [7]. Furthermore, poor mental health currently costs the world economy around US$ 2.5 trillion per year and this figure is expected to rise to US$6 trillion by 2030 [8]. Therefore, tackling NCDs and their risk factors is a major public health challenge that threatens social and economic development throughout the world.

Targeting risk factors that can be modified on an individual level, for example, through lifestyle behavior change, can assist in the prevention and management of NCDs [9, 10]. Lifestyle interventions are effective in the prevention, treatment, or management of coronary heart disease [11, 12], obesity [9], diabetes [13, 14], and cancer [15]. However, successful lifestyle behavior change is often difficult to achieve and is only implemented by a fraction of those in need [16]. Furthermore, the personal everyday coaching by human healthcare professionals that often accompanies lifestyle behavior interventions is neither scalable nor financially sustainable by healthcare systems [17].

Over the last two decades, technological advances in healthcare have created new opportunities in developing evidence-based digital health interventions (DHIs) that use information and communication technology to deliver health services or treatments. Such DHIs now allow medical doctors, health professionals, and other caregivers to scale and tailor lifestyle behavior support to individuals in need at sustainable costs [18, 19]. Behavior change interventions usually contain several potentially active components or behavior change techniques (BCTs), herein referred to as BCTs, to which a change in the target behavior can be attributed. Despite growing research in the field of DHIs (since 2012, over 1,700 systematic reviews have been published on the topic of DHIs [PubMed search “digital health intervention,” filter “systemic review,” date range “2012–2022”]), it remains unclear which BCTs in DHIs are the most effective in changing lifestyle behaviors. Given the relevance of lifestyle behavior interventions for the prevention and treatment of a broad range of NCDs, understanding the BCTs that are consistently eliciting positive behavioral effects could help in the design of more effective and more efficient DHIs that have a greater impact on health outcomes. Therefore, the objective of this study was to conduct a narrative umbrella review of systematic reviews to identify the most effective health-related BCTs in DHIs that address the prevention or management of the most common NCDs and provide recommendations that will guide future research related to the development of effective DHIs.

## Methods

An umbrella review, also referred to as an “overview of reviews” or “review of reviews”, is a synthesis of existing systematic reviews that summarizes the highest level of evidence available, allowing decision makers to gain a clear understanding of a broad topic area [20]. This umbrella review was conducted in accordance with the registered protocol (Open Science Framework Registry; https://doi.org/10.17605/OSF.IO/GE2RS), and the Preferred Reporting Items for Systematic Reviews and Meta-Analyses (PRISMA) 2020 statement [21].

### Search Strategy

We searched the following electronic databases for English language articles published between January 1, 2007 (coinciding with the release of the iPhone and therefore expansion of mHealth studies) and January 24, 2021: OVID (Medline), CINAHL, Web of Science, PsychInfo, and Embase. In addition, we hand-searched reference lists of identified studies and systematic reviews to identify potentially relevant studies. The search was later updated to December 31, 2022. The entire electronic search strategy is presented in Supplementary File 2.

### Inclusion and Exclusion Criteria

Eligible participants, interventions, comparisons, outcomes, and study designs (PICOS) were identified *a priori* and are outlined below.

#### Population

Studies that included adults, or predominantly adults (not more than 25% of included primary studies targeting children or adolescents), aged 18 years or over were eligible for inclusion. Participants could be from a clinical population. Studies that included papers targeting indirectly affected groups of people (e.g., relatives, medical professionals, or parents) were excluded.

#### Intervention

Eligible interventions were those that used e- or mHealth to target the modification of one or more NCD-related risk factors. E- and mHealth interventions are defined as those that use information and communication technologies to improve health and healthcare. eHealth interventions mainly use the Internet while mHealth interventions use mobile devices such as mobile phones, patient monitoring devices, personal digital assistants, and other wireless devices [22]. Reviews were excluded if they included interventions where e- or mHealth functions were not the primary intervention component, for example interventions using human-based coaching via telephone as a primary component. Interventions that took place primarily via social networks (e.g., via Twitter or Facebook) were also ineligible.

#### Comparison

As this was an umbrella review of published systematic reviews, a comparator or control group was not required for inclusion in this study. However, given our interest in effectiveness of interventions and BCTs, we included reviews where evidence from primary experimental studies with an appropriate comparator was available.

#### Health domains and outcomes

Given the breadth of potentially relevant papers, we deemed it necessary to limit the focus of this review to those papers which focused on the prevention or management of the most common NCDs and risk factors. These were:

- NCDs: CVD, cancer, type 2 diabetes, and asthma.
- Risk factors: physical activity, diet, weight management, medication adherence, and substance use (including tobacco smoking, problem drinking, and drug use).

We did not limit our search of papers within these health domains to those reporting on specific outcomes given the purpose of the review was to qualitatively review effective BCTs across a range of health conditions rather than quantitatively synthesize effectiveness on disease-specific health outcomes.

#### Study designs

Published systematic reviews and meta-analyses of randomized-controlled trials (RCTs), nonrandomized controlled trials (NRCTs), case–control studies, quasi-experimental studies, or single-arm pre–post studies reporting both intervention effectiveness outcome data and BCTs were eligible for inclusion. Purely narrative or qualitative reviews and single studies were ineligible.

### Study Selection

Bibliographic records were extracted and imported into Covidence (https://www.covidence.org/). After excluding duplicates, titles and abstracts were independently assessed by two researchers. In the event of discrepancies, a final evaluation was carried out by a third independent person. The full texts of relevant articles were independently reviewed by two researchers for compliance with the inclusion and exclusion criteria. In the event of discrepancies, a final evaluation was carried out by a third independent person.

### Data Extraction

Data were extracted independently by one researcher using a pre-piloted data extraction form prepared using Microsoft Excel. A second author (J.L.M) checked all data extraction for accuracy. Disagreements were resolved by consensus. Extracted data included: (a) title of publication, (b) year of publication, (c) name of the journal, (d) first author, (e) health domain studied, (f) aim of the review, (g) publication period of the primary studies, (h) sample/population: clinical or general population, (i) study design of primary studies, (j) number of primary studies, (k) total sample size, (l) main contents of the intervention, (m) results on intervention effectiveness, (n) risk of bias and evidence certainty information, and (o) findings related to BCTs.

### Quality Assessment

Two reviewers (A.S.-S. and B.F.F.) independently assessed the methodological quality of each systematic review using the AMSTAR 2 rating scale [23]. AMSTAR 2 contains 16 items to critically appraise the methodological aspects of systematic reviews that include randomized or nonrandomized studies of healthcare interventions. Any disagreements were discussed and agreed upon between the two reviewers and a third reviewer (J.L.M) checked all final assessments for accuracy.

### Data Analysis and Synthesis

The information extracted from the individual reviews was summarized narratively and categorized according to health domains. Categories were not determined a priori. Instead, based on the identified reviews, the relevant areas were determined iteratively, to which the individual reviews were then assigned. In this way, it was also possible to consider reviews that combined studies on digital interventions addressing several areas simultaneously (so-called lifestyle interventions, e.g., on nutrition, physical activity, and weight reduction).

The effectiveness of DHIs was first described separately for the individual areas, based on the identified reviews and meta-analyses. Due to heterogeneity of DHIs and outcomes, both across and within health domains, pooled meta-analyses were not possible, therefore effect data are narratively summarized as reported by the authors of the included reviews. In a second step, the effectiveness of BCTs in the respective health area was evaluated. The approach was guided by Michie et al. [24] whereby all available evidence can be used to draw appropriate conclusions about BCTs that can be generalized to other settings and contexts. Initially, the most comprehensive relevant study was identified (e.g., where all features are closest to the specific behavior, intervention delivery, and target population) and an assessment was made on the effect of the relevant BCTs on the respective target behavior, and the generalizability to other health domains. Quantitative evidence, for example from meta-regression or subgroup analysis, were weighted more highly than qualitative summaries such as the frequency of BCTs used within effective interventions. This assessment was then iteratively supplemented and updated based on other relevant studies, considering the relevance and quality of these studies. This approach follows the Bayesian principles of creating an initial level of confidence in a hypothesis that is then progressively supplemented and updated with new information. The extent to which the new information changes the existing information depends on the effect size and relevance of the study. In a third step, the evidence for BCT effectiveness was summarized by health domain using a five tier classification whereby “▼” indicates good evidence of a negative effect of a BCT from subgroup or meta-regression analyses; “O” indicates mixed evidence or no effect of a BCT; “O−” and “O+” indicate some evidence for positive or negative effect of a BCT, respectively, derived from low-quality evidence such as frequency of individual BCTs within effective interventions; and “▲” indicates good evidence of a positive effect of a BCT from subgroup or meta-regression analyses. The aim of this catalog of criteria was to provide clear recommendations for BCT use within each health domain. BCTs were coded using the Behavior Change Technique Taxonomy version 1 [25] where possible, and where coding was not possible due to limited information, the description of the component or technique provided by the original authors was maintained.

### Differences From Protocol

The final protocol of this review slightly deviated from the originally registered protocol (Open Science Framework Registry; https://doi.org/10.17605/OSF.IO/GE2RS) due to significant differences in the reporting of BCTs within research trials and reviews of NCDs and common mental disorders (CMDs). Initially, it was our intention to summarize the evidence for NCDs and CMDs collectively but during data extraction, it became evident that studies of CMDs focus more on the effect of therapeutic strategies than BCTs which precluded qualitative synthesis across these domains. Additionally, the search was updated to December 31, 2022 prior to publication to ensure the most recent best evidence was summarized.

## Results

**Fig. 1.**
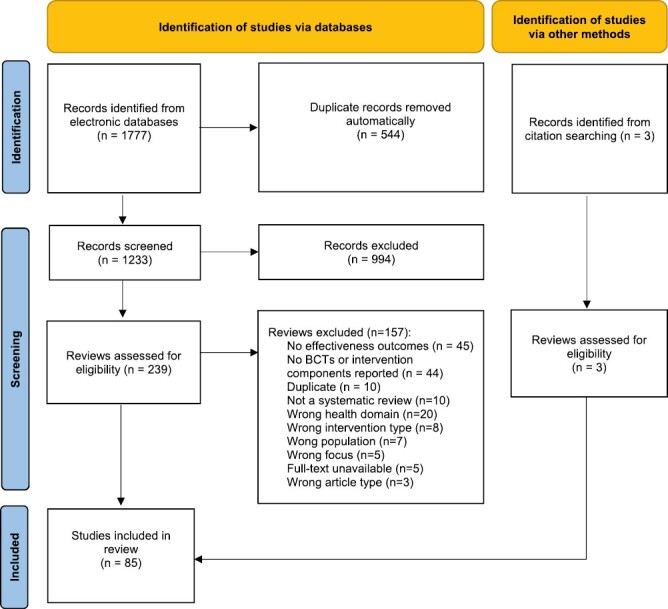
PRISMA flowchart.

After removing duplicates, 1,233 records were identified by electronic searches and three additional articles from hand-searching strategies. After title and abstract screening, 242 full-text articles were reviewed and 86 met the inclusion criteria. Following data extraction, one paper [26] was subsequently excluded due to duplicate data from another included review [27], resulting in 85 included reviews. The PRISMA flowchart with exclusion reasons is presented in Fig. 1. A summary of the included reviews is presented in Supplementary File 1 with the most comprehensive and relevant study for each health domain listed first, followed by other relevant studies. Excluded studies with reasons for exclusion are listed in Supplementary File 3. The evidence for the effectiveness of BCTs is summarized in Table 1.

**Table 1:**
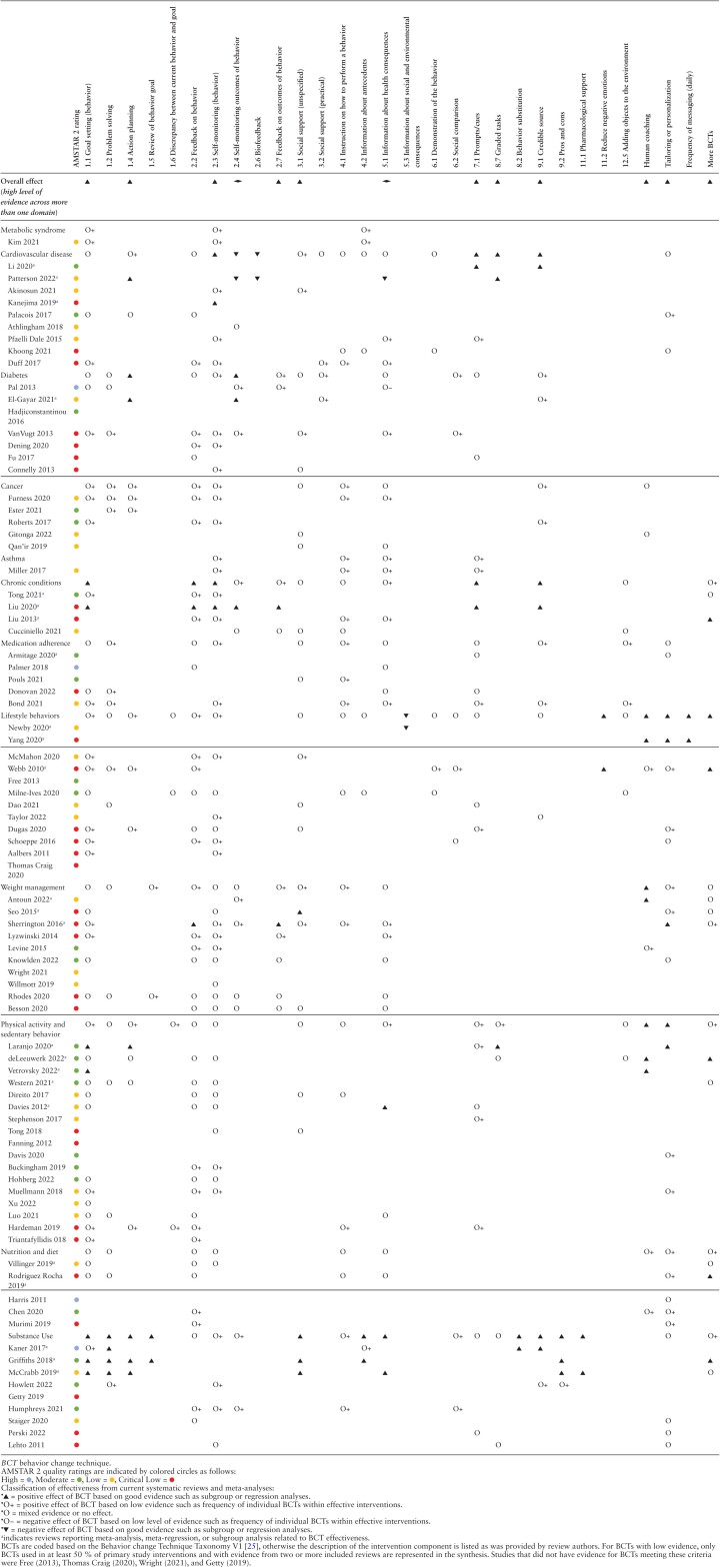
Summary of the Effectiveness of Health-Related Behavior Change Techniques Across Health Domains

### Quality of the Evidence

Only four included reviews were rated as high quality using AMSTAR 2. Most reviews were rated as either moderate (*k* = 22), low (*k* = 29), or critical low (*k* = 30). Reasons for low ratings were largely due to critical flaws whereby there was no preregistered protocol or consideration of bias when interpreting results. Secondly, primary studies within these reviews were typically subject to high risk of bias, and effect sizes were often assessed with high statistical heterogeneity. However, in most cases, the risk of bias was compromised due to the lack of blinding of participants, which is often not possible due to the nature of behavior change interventions. The full quality assessment using AMSTAR 2 is presented in Supplementary File 4.

### Summary of the Evidence

#### Metabolic syndrome

Only one low-quality systematic review and meta-analysis of RCTs focusing on eHealth interventions for patients with, or at risk of, metabolic syndrome (*n* = 2,865) was included [28]. EHealth interventions were found to have moderate to large effects on a range of metabolic risk factors, including waist circumference, glucose and lipid profiles, blood pressure, body mass index, and body mass, when compared with a comparison group. However, no significant between group effect was detected for glycated haemoglobin (HbA1c).

There was insufficient evidence to determine associations between BCTs and eHealth intervention effectiveness.

#### Cardiovascular disease

One integrative review [29], three systematic reviews [30–32], and five systematic reviews with meta-analysis [33–37] on DHIs for patients (total participants *n* = 28,991) with CVD or hypertension were identified. Two reviews were of moderate quality [31, 33], four were low quality [29, 32, 35, 36] and three were of critically low quality [30, 34, 37]. In the most comprehensive meta-analysis of RCTs available, Li et al. [33] found a significant difference in systolic (mean difference [MD] −3.78 mm Hg; *p* < .001; 95% confidence interval [CI] −4.67 to −2.89; *k* = 16) and diastolic (−1.57 mm Hg; *p* < .001; 95% CI −2.28 to −0.86; *k* = 12) blood pressure between SMS- and app-based hypertension self-management interventions and control. In the meta-analysis of seven RCTs by Khoong et al. [37], no significant differences in systolic blood pressure between mobile app-based intervention groups compared with control were reported, but significant decreases at 6-month follow-up were found in the intervention group only (MD −4.10 mm Hg; 95% CI −6.38 to −1.38). Akinosun et al. [36] conducted meta-analyses of RCTs and reported significant main effects of DHIs versus usual care for total cholesterol, high-density lipoprotein cholesterol, low-density lipoprotein cholesterol, physical activity, and food intake, but no differences for body mass index, triglycerides, blood pressure, HbA1c, or other lifestyle behaviors.

Overall, there was good evidence to suggest self-monitoring (behavior), prompts and cues, graded tasks, and credible source are effective BCTs in DHIs for the management of CVD (Table 1). Three reviews reported on the effectiveness of BCTs for self-management of CVD using quantitative analysis. In the most comprehensive review of RCTs, subgroup analyses revealed mHealth interventions offering tailored prompts and cues based on patient health status and readiness, interactive two-way communication with a credible source (i.e., the treating physician), and multiple (vs. single) features were associated with better self-management of hypertension [33]. In a meta-regression comparing the effect of individual BCTs within mHealth apps, action planning and graded tasks were moderately associated with increased physical activity, but negative associations were found for self-monitoring outcomes, receiving biofeedback and information on health consequences [35]. In a meta-analysis of four RCTs [34], self-monitoring of physical activity using a pedometer or accelerometer increased step count in patients with CVD by 2,503 steps/day (95% CI 1,916–3,090; *p* < .05).

#### Type 2 diabetes

Four systematic reviews [38–41] and three systematic reviews with meta-analysis [42–44] on type 2 diabetes self-management (total *n* = 19,368) were identified. One review was of high quality [42], one was moderate quality [44], one was low quality [43], and four were of critically low quality [38–41]. In the Cochrane review of RCTs by Pal et al. [42], there was moderate-quality evidence for a small pooled effect on HbA1c of 2.3 mmol/mol (MD −0.2%; 95% CI −0.4 to −0.1; *k* = 11) for computer-based interventions over comparator groups. The most recent meta-analysis of RCTs on the effectiveness of mobile apps for self-management of type 2 diabetes reported a significant effect on HbA1c (MD −0.38, 95% CI − 0.50 to − 0.25; *p* < .0001; *k* = 21) in favor of intervention groups compared with standard care treatment [43]. There was no evidence that eHealth interventions were effective in improving health-related quality of life or wellbeing [42, 44]. The included systematic reviews reported some evidence of significant improvements in HbA1c [38–41], body weight [39], dietary behavior [39], fasting blood glucose [38, 39], waist circumference [39], cholesterol [38], triglycerides [39], general health behaviors [38], and psychological outcomes [38] in DHI groups, but overall effectiveness in comparison to control or usual care could not be established.

Overall, there was good evidence to suggest action planning and self-monitoring outcomes of behavior are effective BCTs in DHIs for the management of type 2 diabetes (Table 1). Subgroup analyses from El-Gayar et al. [43] showed that action planning and self-monitoring outcome(s) of behavior were present in interventions reporting statistically significant reduction in HbA1c compared with the interventions not supporting these techniques. Pal et al. [42] found that self-monitoring outcome(s) of behavior and feedback on outcomes of behavior were the most commonly used BCTs in interventions that had a significant impact on HbA1c. Fu et al. [40] found that diabetes apps that elicited a significant reduction in HbA1c included feedback on behavior and alert reminders. van Vugt et al. [38] reported that online type 2 diabetes self-management interventions incorporating feedback on behavior, information about health consequences, problem solving, self-monitoring of outcome(s) of behavior, self-monitoring of behavior, and social support were all linked with improvements in health behaviors, clinical outcome measures, and psychological outcomes. Additionally, goal setting (behavior) was linked to improvements in clinical outcomes and social comparison was associated with improvements in psychological outcomes.

#### Cancer

Two systematic reviews [45, 46] and three systematic reviews with meta-analysis [47–49] on DHIs for patients with cancer or cancer survivors (total *n* = 22,653) were included. Two reviews were of moderate quality [45, 48] and three reviews were low quality [46, 47, 49]. The most comprehensive review of RCTs to date [47] found eHealth interventions were more effective in increasing physical activity (standardized mean difference [SMD] 0.34; 95% CI 0.21–0.48; *k* = 15), improving dietary behavior (SMD 0.44; 95% CI 0.18–0.70; *k* = 6) and reducing anxiety (SMD 1.21; 95% CI 0.36–2.07; *k* = 4) in current or former cancer patients compared with control. Modest but favorable trends were also found for quality of life, fatigue, and depression. Significant improvements in moderate to vigorous physical activity (MVPA) were found in a meta-analysis of five RCTs (MD = 49 min per week; 95% CI 16–82; *p* = .004; *k* = 5) and reductions in body mass index/weight in a meta-analysis of RCTs and pre–post studies (SMD −0.23; 95% CI −0.41 to −0.05; *p* = .011; *k* = 4) [48]. Another meta-analysis found connected health interventions to be effective in reducing symptoms of depression compared with usual care (SMD −0.226; 95% CI −0.303 to −0.149; *I*^2^ = 0%; *k* = 7) [49].

None of the included reviews conducted any quantitative analysis on the effectiveness of individual or combinations of BCTs within DHIs for cancer patients and survivors. However, Ester et al. [45] conducted a weight analysis and identified problem solving and action planning to be associated with physical activity. A meta-analysis in which significant pooled effects were detected for physical activity, diet, and anxiety [47], the most commonly used BCTs were goal setting (behavior), self-monitoring of behavior, information about health consequences, problem solving, action planning, feedback on behavior, instructions on how to perform a behavior.

#### Asthma

Only one low-quality systematic review with meta-analysis of RCTs focused on mHealth interventions to improve self-management in asthma patients (*n* = 954) was included [50]. mHealth interventions were found to have moderate to large effects on medication adherence (Hedges *g* = 0.63, 95% CI 0.31–0.94, *p* < .001) and quality of life (*g* = 0.64, 95% CI 0.19–1.08, *p* = .01) when compared with standard treatment. Findings also suggest improvements in well-controlled asthma and a reduction in unscheduled doctor visits. However, for other clinical outcome measures such as lung function, no significant differences were found between groups.

There was insufficient evidence to determine associations between BCTs and asthma medication adherence or clinical outcomes. However, there was an indication that the number of BCTs used fully explained the variance in quality of life. The BCTs used most frequently and in the majority of studies were information about health consequences, self-monitoring of behavior, prompts/cues, and instruction on how to perform a behavior.

#### Chronic conditions

Three systematic reviews with meta-analysis [51–53] and one systematic review [54] exploring effectiveness of DHIs in populations with different chronic conditions were included. The reviews were of moderate [51], low [54], and critically low [52, 53] quality. The most recent meta-analysis of RCTs examining the effectiveness of personalized mobile apps and fitness trackers on lifestyle behavior outcomes reported a moderate positive effect (SMD 0.663, 95% CI 0.228–1.10; *k* = 14) compared with control [51]. Liu et al. [52] examined evidence from RCTs on the effectiveness of mobile apps for self-management of type 2 diabetes and hypertension and reported significant effects on HbA1c (SMD −0.44; 95% CI −0.59 to −0.29; *p* < .001; *k* = 21), systolic blood pressure (SMD −0.17; 95% CI −0.31 to −0.03; *p* = .02; *k* = 16), diastolic blood pressure (SMD −0.17; 95% CI −0.30 to −0.03; *p* = .02; *k* = 14), fasting blood glucose (SMD = −0.29; 95% CI−0.49 to −0.10; *p* = .004), and waist circumference (SMD −0.23; 95% CI −0.43 to −0.04; *p* = .02) over control [42]. Liu et al. [53] also reported significant reductions in daytime systolic blood pressure (SMD −0.27; 95% CI −0.44 to −0.10; *p* = .002) and diastolic blood pressure (SMD −0.17; 95% CI −0.33 to −0.01; *p* = .03) in Internet interventions targeting exercise and diet for the modification of blood pressure.

Overall, there was good evidence to suggest goal setting (behavior), feedback on behavior, self-monitoring (behavior), prompts and cues, and credible source are effective BCTs in DHIs for the management of chronic conditions (Table 1). In a meta-regression conducted by Tong et al. [51], total number of BCTs in personalized features, or the number of personalized features in mobile apps or fitness trackers had no effect on lifestyle behavior outcomes. In contrast, Liu et al. conducted subgroup analysis to show that interventions using five or more BCTs are more effective in reducing blood pressure than those with fewer than five BCTs [53]. Subgroup analyses from Liu et al. [52] showed that self-monitoring outcomes of behavior (blood pressure and blood glucose), self-monitoring behavior (medication monitoring), automated feedback on behavior and outcomes of behavior (data visualization), personalized goal setting, reminders, educational materials, and communication with a credible source (healthcare provider) were associated with positive outcomes.

#### Multiple lifestyle behaviors

We identified 12 reviews on multiple lifestyle behaviors (*n* > 137,943), of which five were focused on NCD prevention [55–59], six were on both prevention and management of NCDs [60–65], and one was related to health in general [66]. Two reviews were of moderate quality [60, 61], four were low quality [55, 57, 62, 63] and six were of critically low quality [56, 58, 59, 64–66]. Evidence from a recent meta-analysis of RCTs on eHealth lifestyle behavior interventions targeting CVD risk reduction in men found eHealth intervention to be more effective than control or comparison conditions for body mass index, body weight, waist circumference, systolic and diastolic blood pressure [57]. Yang et al. [56] reported small but significant effects of eHealth interventions on health mediating variables (*d* = 0.29; 95% CI 0.20–0.38; *p* < .001; *k* = 49), health behaviors (*d* = 0.28; 95% CI 0.18–0.38; *p* < .001; *k* = 52), and health outcomes (*d* = 0.32; 95% CI 0.21–0.42; *p* < .001; *k =* 40) compared with control. Two reviews reported small (*d* = 0.16; 95% CI 0.09–0.23; *k* = 85) [66] to moderate (*d* = 0.39 ± 0.37) [59] average effect sizes for eHealth interventions over comparison conditions. However, other systematic reviews reported inconclusive findings with respect to DHI effectiveness. While Schoeppe et al. [58] found the majority (17/23; 74%) of app-based interventions targeting diet, physical activity, and/or sedentary behavior to be effective, Milne-Ives et al. [61] found only 24% (12/51) to be effective and 31% (16/51) partially effective (i.e., differences in some but not all outcomes) in improving health or behaviors over control or comparison conditions. Others reported few effective studies within their reviews of mHealth [62, 64] or e- and mHealth [65] interventions targeting health behavior change.

Overall, there was good evidence to suggest that reducing negative emotions, human coaching, and tailoring or personalization are effective strategies to improve health behaviors via DHIs (Table 1). In moderator analyses, Yang et al. [56] reported that tailoring or personalization of content based on users’ input and access to a mentor or coach were found to increase intervention effectiveness compared with interventions without these components, while Newby et al. [55] found that the BCT “information about social and environmental consequences” led to a small negative effect on self-efficacy. Webb et al. [66] reported significant moderate effects on health behaviors for interventions that provided BCTs “reduce negative emotions” (stress management) and general communication skills training, and small effects for demonstration of the behavior, problem solving, social comparison, goal setting (behavior), action planning, and feedback on behavior. In their review of apps for NCD prevention, Schoeppe et al. [58] found those which used goal setting (behavior), self-monitoring of behavior, and feedback on behavior were particularly effective for diet and physical activity behavior change. Dugas et al. [64] reported that prompts and cues, goal setting (behavior), action planning, and personalization in general, were most commonly used within effective mHealth lifestyle behavior interventions.

#### Weight management

Five systematic reviews [67–71] and five systematic reviews with meta-analyses [72–76] were specifically focused on weight management for NCD prevention (*n* > 62,567) largely focusing on both physical activity and dietary behaviors. Two studies were rated moderate quality [67, 68], three were low quality [69, 70, 72], and five were of critically low quality [71, 73–76]. Evidence from meta-analyses of RCTs suggest that DHIs are effective in achieving weight loss in general population (−1.99 kg; 95% CI −2.19 to −1.79) [72] and adults with overweight or obesity (pooled effect size = 0.43; 95% CI 0.252 to 0.609; *k* = 11; *p* ≤ .01) [75] compared with control. In a meta-analysis of RCTs, eHealth interventions were also shown to be effective in reducing in waist circumference in the general population compared with minimal intervention (mean change 2.38 cm, 95% CI 1.51–3.25; *k* = 24; *p* < .001) [73]. In their review of technology-assisted weight loss interventions, Levine et al. [67] found 75% of included studies achieved weight loss at the end of the study compared with controls. However, more recent reviews of DHIs for weight reduction in normal weight or overweight adults [68, 71] have found significant weight loss in favor of the intervention arms in only 50% of the individual studies included. Furthermore, Rhodes et al. [76] found that only 27% of studies had a positive effect on gestational weight gain in pregnant women in comparison with control groups.

Overall, there was good evidence that human coaching is effective in promoting weight loss in DHIs. One meta-regression of BCTs within Internet-based interventions identified social support as a technique associated with greater decreases in waist circumference, while self-monitoring of outcomes of behavior, goal setting (behavior), or outcomes of behavior, motivational interviewing, or the number of BCTs had no effect [73]. In another subgroup analysis, no associations were found between weight loss and any specific type or number of mobile app features, however combining a mobile app, with an activity tracker, and behavioral intervention, or intensive behavior coaching or feedback by a human coach, showed a statistically significant weight loss [72]. Another meta-analysis of RCTs reported greater weight loss for Internet-delivered interventions providing personalized feedback compared with control groups receiving no personalized feedback [74].

#### Physical activity and sedentary behavior

Eight systematic reviews [77–84] and nine systematic reviews with meta-analyses [85–93] focusing on only physical activity and/or sedentary behavior (*n* = 154,654) were included. The quality of the reviews was moderate [77–79, 85–88], low [80–82, 89–91], or critically low [83, 84, 92, 93]. Several meta-analyses of RCTs or NRCTs have shown that DHIs have a significant small to moderate effect on increasing physical activity [85–89, 93] and reducing sedentary behavior [90, 91]. mHealth interventions were found to increase daily step count by, on average, 926–1,850 steps per day [85, 87]. However, findings from other reviews are inconclusive. For example, Tong and Laranjo [92] reported a nonsignificant effect of mHealth interventions on physical activity outcomes, while Xu et al. [81], Buckingham et al. [78], and Davis et al. [77] found that only 50%, 56%, and 62.5% of mHealth interventions targeting physical activity were effective compared with control groups, respectively. The meta-analysis of RCTs by Direito et al. [90] failed to find any significant effects of mHealth interventions for total physical activity, MVPA, or walking.

Overall, there was good evidence to suggest that human coaching and tailoring or personalization strategies in DHIs are effective in increasing physical activity, however evidence for the effectiveness of other BCTs in physical activity interventions is largely mixed. In their meta-analysis of eHealth interventions, Davies et al. [89] reported that educational components were the only significant moderators of physical activity change. However, subgroup analyses from Laranjo et al. [85] linked four BCTs or BCT clusters to intervention effectiveness; text messaging, personalization, graded tasks, and goals and planning. The authors also found that automated monitoring and feedback did not reduce effectiveness. In a subgroup analysis by de Leeuwerk [86] theory-based interventions with activity trackers and coaching by a health professional were more effective than interventions without these features. Furthermore, interventions with seven BCTs or more were more effective than those with fewer than seven BCTs. By contrast, in a meta-regression by Western et al. [88], no associations were found for the number or type of BCTs. Hardeman et al. [83] reported that goal setting, action planning, discrepancy between current behavior and goal, feedback on behavior, prompts and cues, social reward, and instruction on how to perform a behavior were present in effective just-in-time adaptive interventions targeting physical activity. Triantafyllidis et al. [84] found that personalized behavioral goals and motivational coaching characterize effective eHealth interventions for physical activity promotion in healthy adults.

#### Nutrition and dietary behaviors

Two systematic reviews [94, 95] and three systematic reviews with meta-analysis [96–98] on nutrition and/or dietary behaviors (*n* > 70,601) were included. One review was of high quality [98], one was of moderate quality [94], and three were of low [96] or critically low quality [95, 97]. In the recent meta-analysis of RCTs and quasi-experimental studies from Villinger et al. [96], app-based mobile interventions were found to have a small to moderate overall pooled effect (*g* = 0.33; CI 0.21–0.44; *p* < .001) on nutritional outcomes. Effects were small for behavioral outcomes (*g* = 0.19; CI 0.06–0.32; *p* = .004; *k* = 21), and when separating behavioral outcomes into calorie and fruit and vegetable intake, only the effect for fruit and vegetable intake reached statistical significance (*g* = 0.32; CI 0.15–0.50; *p* < .001). The authors also reported a small to moderate effect (*g* = 0.23; CI 0.11–0.36; *p* < .001; *k* = 34) on nutrition-related health outcomes, such as obesity indices, blood pressure, blood lipids, and blood glucose. In a review of RCTs, Harris et al. [98] found interactive and tailored computer-based interventions had a positive effect on daily fruit and vegetable intake (weighted mean difference = +0.24; 95% CI 0.04–0.44; *k* = 12) and total daily energy consumed from fat (weighted mean difference = −1.4%; 95% CI −2.5 to −0.3; *k* = 10).

Despite reporting significant effects for app-based interventions on nutritional outcomes, in a subgroup analysis, Villinger et al. [96] failed to find any significant effect of specific BCTs within the reviewed apps. Rodriguez Rocha and Kim [97] also reported no association between specific BCTs within web- and SMS-based interventions and effectiveness on fruit and vegetable intake; however, they found higher efficacy in interventions with seven or more BCTs and in tailored programs.

#### Medication adherence

One systematic review with meta-analysis [99] and four systematic reviews [100–103] on medication adherence were included (*n* = 122,874). Reviews were of high [100], moderate [99, 101], low [102], and critical low [103] quality. A Cochrane review of four RCTs with at least 12-month follow-up reported low quality and uncertain evidence relating to the effects of mobile phone-delivered interventions to increase adherence to medication prescribed for the primary prevention of CVD [100]. However, in a more recent review of RCTs, Armitage et al. [99] found that patients with a range of conditions, including CVD, depression, Parkinson’s, psoriasis, and multimorbidity, who participated in medication adherence interventions delivered by mobile apps were more likely to adhere to prescribed medications (odds ratio [OR] 2.120; 95% CI 1.635–2.747; *k* = 9) than those who did not use such interventions. In two other systematic reviews of RCTs [101, 102], the majority of primary studies (29/38; 76%) reported improvements in medication adherence in DHI groups compared with control or usual care with Cohen’s *d* effect sizes ranging from very small (0.06) to large (0.8).

In a meta-regression of BCTs coded in more than three, but less than six of the nine reviewed studies, Armitage et al. [99] found no significant associations between the BCTs used and intervention effectiveness. Pouls et al. [101] noted strong evidence for a positive effect of strategies to teach skills (e.g., instructions on how to perform a behavior), to facilitate communication or decision-making, and to improve healthcare quality. Donovan et al. [103] reported improved medication adherence in 90% of studies using BCTs to target obtaining medication, 88% of studies including problem solving, and 60% of studies including social reward.

#### Alcohol

One high-quality Cochrane review of RCTs (*n* = 34,390) by Kaner et al. [27] found that participants receiving a DHI consumed 22.8 g (95% CI 15.4–30.3) less alcohol per week than control group participants at the longest reported follow-up. The authors provide moderate-quality evidence to support the use of DHIs in reducing the frequency and intensity of drinking per week, as well as the number of binges per week and the risk of being a binge drinker at longest follow-up.

Evidence from the adjusted meta-regression model (including only BCTs with regression coefficients >23) indicated that behavior substitution, problem solving, and credible source are associated with reduced alcohol consumption. In an unadjusted model, goal setting, and information about antecedents were also significantly associated with reduced alcohol consumption. There was no association between the number of BCTs used and effectiveness.

#### Tobacco

One moderate-quality [104] and one low-quality [105] systematic review with meta-analyses on smoking cessation (total *n* = 68,706) was included. McCrabb et al. [105] conducted a meta-analysis of 45 RCTs of Internet-based smoking cessation interventions and demonstrated significant short-term (overall OR = 1.29; 95% CI 1.12–1.50; *p* = .001) and long-term (overall OR = 1.19; 95% CI = 1.06–1.35; *p* = .004) effects on smoking abstinence compared with control. Interventions were also found to be effective for “prolonged abstinence” (OR = 1.43; 95% CI 1.09–1.87; *p* = .009) and “30-day point prevalence abstinence” (OR = 1.75; 95% CI 1.13–2.72; *p* = .013). Griffiths et al. [104] also found DHIs significantly increased the odds of quitting smoking during pregnancy compared with control groups (OR = 1.44; 95% CI 1.04–2.00; *p* = .03).

Using meta-regression, McCrabb et al. [105] found that goal setting (behavior) increased short-term effectiveness of Internet smoking cessation programs, while problem solving, action planning, social support, information on health consequences, pros and cons, and pharmacological support are associated with both short- and long-term treatment effectiveness. Similarly, in a subgroup analysis, Griffiths et al. [104] found information about antecedents, action planning, problem solving, goal setting (behavior), review behavior goals, social support, and pros and cons to be significantly associated with DHI effectiveness for smoking cessation in pregnancy.

#### Substance use: drugs, alcohol, and/or tobacco

Five systematic reviews and one meta-analysis on addictive behaviors (total *n* > 49,710) including mostly drug, tobacco, and/or alcohol use [106–110] but also alcohol use, gambling, and eating disorders [111] were included. Reviews were rated moderate [106, 111], low [108], and critically low [107, 109, 110] quality. Evidence from a meta-analysis of RCTs and quasi-experimental studies [107] on mobile-delivered contingency management interventions promoting abstinence from drugs, alcohol, and tobacco suggests interventions are effective for percentage of negative samples (*d* = 0.94; 95% CI 0.63–1.25), quit rate (*d* = 0.46; 95% CI 0.27–0.66), and longest duration abstinent (*d* = 1.08; 95% CI 0.69–1.46). Howlett et al. [106] found 34% of studies had very promising evidence (a statistically significant improvement in the primary outcome compared with control) and 42% quite promising evidence (either a statistically significant improvement within the experimental group or the between-subjects difference was significantly greater than the control group) of DHI effectiveness in reducing alcohol or substance misuse. Other evidence was largely mixed. In the review by Staiger et al. [108] only 30% of mobile app interventions reported significant reductions (with small to moderate effect sizes) in substance use after treatment or at follow-up, compared with a comparison condition.

There was no quantitative evidence available on the associations between BCTs and substance use outcomes. However, Howlett et al. [106] conducted sensitivity analysis of promise ratios and found self-monitoring of behavior, avoidance/ reducing exposure to cues for behavior, pros and cons, behavior practice/rehearsal, and credible source to be the most promising BCTs for alcohol misuse interventions, while problem solving and self-monitoring of behavior were the most promising for substance misuse interventions. Monetary incentives and escalating reinforcement were present in mHealth interventions included in the meta-analysis by Getty et al. [107], which reported large to moderate pooled effect sizes. Humphreys et al. [111] also reported that feedback on behavior, self-monitoring of behavior or outcomes of behavior, instruction on how to perform the behavior and social comparison were present in the effective high-quality studies they reviewed.

## Discussion

This umbrella review summarizes the evidence for the effectiveness of DHIs targeting the prevention and management of NCDs and systematically identifies which BCTs within these interventions are effective in improving health outcomes in adults. Using evidence from 85 reviews, spanning 12 health domains, with primary research published over a 37-year period and comprising over 865,000 individual participants, our findings suggest that DHIs are effective in improving outcomes for patients with CVD, cancer, type 2 diabetes, and asthma when compared with control or usual care conditions. Furthermore, DHIs are effective in improving health-related behaviors, including physical activity, sedentary behavior, diet, weight management, medication adherence, and abstinence from substance use in both general and clinical populations. There was strong evidence from more than one health domain that DHIs incorporating a credible source (such as communication with a professional or counselor), social support, graded tasks, prompts, and cues (in the form of messaging and reminders), and self-regulatory BCTs including goal setting, action planning, self-monitoring behavior, and feedback on outcomes of behavior are associated with greater effectiveness. Additionally, having access to a human coach, tailored or personalized content and more BCTs rather than fewer BCTs enhanced effectiveness.

Optimizing behavior change interventions is necessary to disentangle effective components from ineffective components to improve overall effectiveness. Therefore, it is crucial to identify which components, under which conditions, lead to convincing and repeatable positive impacts on behavior. Here, we provide, for the first time, a comprehensive overview of the BCTs that can be linked to effectiveness and therefore should be present in future interventions.

We found strong evidence for the incorporation of tailored reminders and professional human support (e.g., from a treating physician) in interventions that largely targeted the modification of multiple lifestyle behaviors, such as physical activity and diet. The need for professional support alongside digital tools echoes previous research highlighting the importance of human coaching [112] or lifestyle behavior support in a face-to-face setting [113]. Indeed, many commercially available health behavior interventions offer human support or coaching as a key feature [114]. However, reliance on human support within DHIs vastly reduces their scalability and cost-effectiveness potential. Given that personalized support appears to be crucial for effective behavior change, alternative delivery strategies, such as using conversational agents [114, 115, 116, 117], should be explored as a matter of priority to develop scalable and effective DHI solutions.

We also found strong evidence for the inclusion of self-regulatory BCTs in interventions targeting people with chronic conditions, including CVD and diabetes. Specifically, a combination of BCTs including goal setting, self-monitoring, and feedback seems to be consistently aligned with enhanced effectiveness. These techniques, which derive from control theory, have previously been found to increase the effectiveness of physical activity and healthy eating interventions delivered within community, primary care, and workplace settings [118, 119]. Therefore, our review extends the current evidence and confirms the effectiveness of these BCTs within digital formats. In line with previous reviews, we also found clear evidence that DHIs should be tailored and personalized to individuals [51] to improve health outcomes and should include more rather than fewer BCTs [120].

Similarly, there was strong evidence to support the inclusion of self-regulatory BCTs, such as goal setting, problem solving, and planning, in interventions targeting alcohol reduction and smoking cessation. Behavioral substitution and credible source were also identified as effective for alcohol consumption while reviewing behavioral goals, information about health consequences, pros and cons, social support, and feedback and monitoring were effective for tobacco cessation. These BCTs align closely with those seen in other lifestyle behavior interventions highlighting their effectiveness for discouraging as well as encouraging a target behavior.

Currently, there is either no or inadequate evidence to permit analysis of effective BCTs in DHIs targeting cancer and asthma self-management. Evidence from reviews of nutritional and medication adherence interventions was unable to establish any association between specific BCTs and intervention effectiveness. This highlights a need for more rigorous research on the effectiveness of individual or combinations of BCTs in these domains.

Several steps need to be taken to advance the current state of evidence on BCT effectiveness within DHIs. Firstly, more primary experimental studies with consistently reported outcomes and appropriate statistical power are needed to (a) understand the comparative effectiveness of individual BCTs or combinations of BCTs (e.g., factorial trials), (b) optimize the timing of BCT delivery depending on participant responsiveness to a DHI (e.g., sequential multiple assignment randomized trials), and (c) to understand the proximal effectiveness of BCTs on rapidly changing and dynamic behaviors (e.g., micro-randomized trials). Secondly, future research should explore contextual factors such as intervention mode of intervention delivery, setting, population, and design features, including user interface and experience (UI/UX), gamification elements, or use of persuasive design, and how these interact with BCTs and impact user engagement and effectiveness of DHIs. Particular attention should be given to optimizing human support given the effectiveness of this component but the current resource implications that preclude large-scale implementation. Thirdly, studies should consider demographic and geographic factors, cultural differences, digital literacy, and accessibility issues, since these factors can significantly influence the effectiveness of DHIs. Fourth, more longitudinal studies are needed to understand the long-term effectiveness of DHIs in sustaining behavior change and what role adaptive interventions might play over time. Finally, future research should focus on how artificial intelligence and machine learning can be integrated into DHIs to provide personalized and adaptive interventions [121]. These technologies can also be used for real-time analysis of collected data to identify trends and patterns, predict outcomes, and provide feedback to the users or clinicians.

### Strengths and Limitations

The strengths of the present review include a rigorous and systematic methodology that was prospectively registered, the comprehensive examination of DHIs targeting the most common NCDs and related risk factors, the use of AMSTAR 2 to assess review quality, coding of BCTs using the BCTTv1, and adherence to the PRISMA guidelines. However, there are also several limitations to consider. We were not able to review all health conditions related to a specific domain due to the vast number of research articles, therefore we were limited to the most common NCDs. We did not describe or cite all primary studies from included reviews due to the large number of trials represented (*k* = 2,164). Because the included reviews were not mutually exclusive in their eligibility criteria, there may be individual trials that are represented in more than one review, particularly for trials related to behavioral interventions. However, given that our review provides only a narrative summary of effect sizes for each health domain, we do not expect this to alter our conclusions. Another limitation of the review includes the omission of grey literature or studies published in languages other than English. We were also limited to the information reported by the authors in the original reviews, which employed a variety of methodologies for data synthesis, presentation, and quality assessment. Furthermore, we were unable to identify the sociodemographic status of the populations studied within the included reviews, therefore our findings may not be representative of all segments of the adult population. Finally, our findings should be regarded with some caution due to the relatively low quality of the included reviews and the high heterogeneity found in meta-analyses of effects. It is also possible that primary studies within included reviews were subject to high methodological heterogeneity whereby study designs, intervention designs, behavior change components, and outcomes were highly variable between trials and thus could explain why DHI effectiveness for certain health domains is not yet conclusive.

## Conclusions

This is the first umbrella review to provide systematic evidence on the effectiveness of BCTs within DHIs across several health domains linked to the management and prevention of NCDs. This knowledge is critical for the future development and upscaling of DHIs and will help to establish best practice guidelines. Given the additional benefits of DHIs in terms of cost-effectiveness and scalability compared with face-to-face interventions, there is convincing evidence to support the promotion of DHIs for the prevention and management of NCDs. However, further research is needed to identify the effectiveness of DHIs across all population segments. Furthermore, higher-quality evidence from rigorous experimental trials is needed to understand which BCTs work for which health domains and in which contexts.

## Supplementary Material

kaad041_suppl_Supplementary_File_1

kaad041_suppl_Supplementary_File_2

kaad041_suppl_Supplementary_File_3

kaad041_suppl_Supplementary_File_4

## Transparency Statements

- This study was registered after the study began. The study is registered at https://doi.org/10.17605/OSF.IO/GE2RS.
- The analysis plan was registered prior to beginning data extraction at https://doi.org/10.17605/OSF.IO/GE2RS.
- As this is an umbrella review of existing evidence, all data are already available in published manuscripts. The extracted data used to compile our results are available at https://osf.io/m9gec/.
- There is no analytic code associated with this study.
