# Supplementary material for: Effective Behavior Change Techniques in Digital Health Interventions for the Prevention or Management of Noncommunicable Diseases: An Umbrella Review
Source: Ann Behav Med. 2023 Aug 25;57(10):817–35. doi: 10.1093/abm/kaad041 (PMC10498822; doi:10.1093/abm/kaad041)
Supplement: kaad041_suppl_Supplementary_File_1 [file kaad041_suppl_supplementary_file_1.docx]

Supplementary File 1. Overview of included reviews under each health domain.

| **Reference and review type** | **Technology type and mode of delivery** | **Review period** | **N Primary studies and study designs** | **Population and sample size** | **Outcomes of interest** | **Intervention effectiveness** | **Effectiveness of BCTs or components** | **Most frequently used BCTs and components** | **Evidence quality** | **AMSTAR 2**  **Rating** |
| --- | --- | --- | --- | --- | --- | --- | --- | --- | --- | --- |
| **Metabolic Syndrome** | | | | | | | | | | |
| Kim 2021 [28]  Systematic review and meta-analysis | eHealth  Web-based programs, e-mail counselling, mobile devices such as cell phones, patient monitoring devices, personal digital assistants (PDAs), social media interventions, and other wireless devices | Searches:  2000-2018  Publications:  2007-2018 | 18 studies  RCTs | Clinical population: adults with metabolic syndrome or metabolic risks  N=2,865* | Metabolic disease risk factors including waist circumference, blood pressure, cholesterol profile, lipid profile, BMI, body mass, HbA1c. | *Main effects of intervention versus comparison group:*   - Waist circumference (SMD −0.35; 95% CI −0.54 to −0.15; I^2^=60%; k=11) - HDL (SMD 0.77; 0.20 to 1.34; I^2^=95%; k=12) - LDL (SMD -0.24; −0.37 to −0.12; I^2^=0%; k=9) - TG (SMD -0.14; −0.26 to −0.03; I^2^=0%; k=11) - SBP (SMD -0.25; −0.37 to −0.14; I^2^=44%; k=11) - DBP (SMD -0.32; −0.51 to −0.13; I^2^=58%; k=11) - Fasting Glucose (SMD -0.31; −0.42 to −0.19; I^2^=0%; k=8) - BMI (SMD -1.36; −2.21 to −0.51; I^2^=97%; k=10) - Body mass (SMD -1.34; −2.04 to −0.64; I^2^=98%; k=16) - No significant between group effect for HbA1c | *Quantitative analysis: Not reported* | - 4.2 Information about antecedents [education on healthy diet and physical activity] (k=unclear) - 1.1 Goal setting (behaviour) (k=unclear) - 2.3 Self-monitoring of behaviours (k=unclear) | *Cochrane Collaboration’s Risk of Bias:*   - Overall, most of the RCTs were judged to raise some concerns in at least one domain, but not to be at high risk of bias. - Most studies were at high risk of bias for allocation concealment but due to the nature of the studies it was not possible to blind participants to intervention allocation. | **Low**  One critical flaw (no pre-registered protocol)  More than one non-critical weakness (no justification for selection of study design, excluded studies not listed, no information on funding sources) |
| **Cardiovascular Disease** | | | | | | | | | | |
| Li 2020 [33]  Systematic review and meta-analysis | mHealth  SMS text messages, (50% automated messages and 50% customized messages based on the feedback of participants), smartphone apps, automated emails, wireless BP monitoring, digital medications automated or interactive voice calls, electronic medication trays | Searches:  2010-2019  Publications:  2010-2019 | 24 studies  RCTs | Clinical population: adults with a primary diagnosis of hypertension  N=8,933 | Blood pressure | *Main effects, intervention versus control:*   - SBP (MD −3.78 mmHg; 95% CI −4.67 to −2.89; I^2^=49%; k=16). - DBP (MD −1.57 mmHg; 95% CI −2.28 to −0.86; I^2^=39%; k=12) - Odds ratio of BP control in the intervention group was 1.42 times more than that in the control group (95% CI 1.23 to 1.65; I^2^=50%; k=10). | *Subgroup analysis:*  Larger overall effects were observed for trials including:   - 7.1 Prompts/cues [with tailored frequency] - 9.1 Credible source [a patient-doctor interactive loop] - multifaceted functions   *Other relevant findings:*   - Self-management education through mHealth was effective in increasing patients’ knowledge of hypertension and a healthy lifestyle, medication management, and self-efficacy. | - 4.2 Information about antecedents [Education about hypertension/ healthy lifestyle] (k=39), - 2.4 Self-monitoring of outcome(s) of behavior   [blood pressure] (k=17)   - 1.1 Goal setting (behavior) (k=15) - 7.1 prompts/cues [medication adherence] (k=10) - 9.1 Credible source (k=4) - 1.4 action planning (k=3) - 11.2 Reduce negative emotions [stress management] (k=1) | *Cochrane Collaboration’s Risk of Bias:*  overall risk of bias was relatively high (no studies were free of bias).  The funnel plot of the comparison of SBP and DBP did not show any extreme asymmetry or outliers, which suggests no significant publication bias. | **Moderate**  No critical flaws  More than one non-critical weakness (no justification for selection of study design, excluded studies not listed) |
| Patterson 2022 [35]  Systematic review and meta-regression | mHealth  Smartphone applications or computer tablet applications utilised either as a stand-alone program or as part of an intervention package (e.g. face-to-face cardiac rehabilitation, phone counselling, activity trackers such as a smart watch) | Searches:  2007-2020  Publications:  *Not reported* | 19 studies  RCTs, cluster RCTs, and quasi-experimental studies | Clinical population: CVD patients  N=1,543 | Physical Activity and sedentary behaviour | *Main effects: Not reported* | *Meta regression:*  Comparing the effect of individual BCTs present in apps versus not present in apps, on PA:   - 2.4 Action planning: medium, positive association (*β* = 0.42, 90% CrI 0.07 to 0.78; k=7/12) - 8.7 Graded tasks: medium, positive association (*β* = 0.33, 90% CrI -0.04 to 0.67; k=6/12) - 2.4 Self-monitoring of outcome(s) of behaviour: medium, negative association (β = −0.47, 90% CrI -0.79 to -0.16; k=5/12) - Biofeedback: medium, negative association (β = −0.47, 90% CrI -0.81 to -0.15; k=7/12) - 5.1 Information about health consequences: medium, negative association (β = − 0.42, 90% CrI -0.74 to -0.07; k= 6/12) - Action planning and self-monitoring of outcome(s) of behaviour and biofeedback are collinear. | - 2.3 Self-monitoring of behaviour (k=19; 100%) - 12.5 Adding objects to the environment (k=19; 100%) - 2.2 Feedback on behaviour (k=16; 84%) - 9.1 Credible source (k=14; 74%) - 2.6 Biofeedback (k=12; 63%) - 3.1 Social support (unspecified) (k=12; 63%) - 1.1 Goal setting (behaviour) (k=12; 63%) - 7.1 Prompts/cues (k=11; 58%) - 2.4 Self-monitoring of outcome(s) of behaviour (k=10; 53%) | *Revised Cochrane risk-of-bias (RoB-2) and ROBINS-I:*   - Risk of bias was high for all RCTs (*n*=10) and NRCTs (*n*=3) - The high risk of bias was primarily due to lack of blinding of outcome assessors and participants. - The cohort studies (*n*=6) were considered moderate risk of bias primarily due to small sample sizes and using self-report outcome measures. | **Low**  One critical weakness (no consideration of bias when interpreting results)  More than one non-critical weakness (missing information on inclusion criteria regarding comparator, no justification for selection of study design, study selection not done in duplicate, excluded studies not listed, no information on funding sources, no discussion of heterogeneity |
| Akinosun 2021 [36]  Systematic review and meta-analysis | eHealth and mHealth  Cell phones, smartphones, personal computers (laptops and desktops), and wearables using the internet, software applications, and mobile sensors. | Searches:  2000-2019  Publications: 2002-2019 | 25 studies  RCTs | Clinical population: patients with CVD  N=5,779 | CVD risk factors including cholesterol profile, lipid profile, BMI, blood pressure, HbA1c, physical activity, diet, alcohol intake, smoking, medication adherence | *Main effects, intervention versus usual care:*   - TC (SMD -0.29; 95% CI -0.44 to -0.15; k=9) - HDL (SMD−0.09; 95% CI −0.19 to 0.00; k=9) - LDL (SMD−0.18; 95% CI −0.33 to −0.04; k=12) - PA (SMD 0.23; 95% CI 0.11 to 0.36; k=14) - Physical Inactivity (RR 0.54; 0.39 to 0.75; k=4) - Food Intake (RR 0.79; 0.66 to 0.94; k=6) - No significant between group effect for BMI, TG, SBP, DBP, HbA1c, alcohol intake, smoking, medication adherence | *Quantitative analysis: Not reported*  *Other relevant findings:*  Interventions effective in changing Total Cholesterol, HDL, and LDL included:   - 2.4 Self-monitoring of outcome(s) of behavior - 3.1 Social support [one-on-one] - 15.1 verbal persuasion about capability | - 2. Feedback and Monitoring (k=unclear) - 3.1 Social support [incl. one-on-one] (k=unclear) - 15.1 verbal persuasion about capability] (k=unclear) - 8.1 Behavioural practice/rehearsal (k=unclear) - personalization (k=unclear) | *Modified Cochrane Collaboration AUB KQ1 Risk of Bias Assessment Tool:*   - Proportion bias at baseline was reported in 16% (4/25) of the included studies as high risk. - Intervention dropout was recorded in 32% (8/25) of the included studies at less than 10% of participants per study. - Dropouts greater than 10% of study participants were recorded as high risk for treatment efficacy. | **Low**  One critical flaw (no consideration of bias when interpreting results)  More than one non-critical weakness (no explanation for selection of study designs, no information on funding sources, no discussion about heterogeneity, publication bias reported but not discussed) |
| Kanejima 2019 [34]  Systematic review and meta-analysis | eHealth and mHealth    Web-based programs, smartphones, tablets or wearables; pedometers or accelerometers were used as the self-monitoring and measurement tool. | Searches:  Up to 2017  Publications:  2005-2016 | 6 studies  RCTs | Clinical population: participants who had CVDs and were outpatients from the recovery to maintenance phase  N=693 | Physical activity | *Main effects: Not reported* | *Quantitative analysis:*   - Self-monitoring increased PA by (MD) us (95% CI 1916 to 3090, p < 0.05; I^2^=80%; k=4). | The review focused on self-monitoring:   - 2.3 Self-monitoring of behaviors (n=6)   In addition to self-monitoring:   - 3.1 Social Support (unspecified) [counseling] (n=5) - 1.1 Goal setting (behavior) (n=6) | *Cochrane Collaboration’s Risk of Bias:*   - One study had low risk of bias. The remaining studies had unclear to high risk of bias across domains. - A high percentage of the studies showed unknown risk of bias in terms of blinding outcome assessment and high risk of bias in terms of incomplete outcome data. | **Critical Low**  More than one critical flaw (no pre-registered protocol, limited search strategy, no assessment of publication bias)  More than one non-critical weakness (no explanation for selection of study designs, study selection and data extraction not done in duplicate, excluded studies not listed, no information on funding sources, no assessment of impact of risk of bias) |
| Palacios 2017 [31]  Systematic review | eHealth  Internet. In some studies, e-mail reminders and/or motivational SMS were sent. | Searches: unknown – 2016  Publications: 2003-2015 | 7 studies  Parallel group RCT, randomized cross-over or sequential-controlled, or cluster randomized trials | Clinical population: coronary heart disease patients  N=1,321 | Physical activity, QoL | *Main effect: Not reported - results could not be synthesized.*  *Other relevant findings:*   - 4/6 studies found significant improvements in PA in favour of the intervention compared with control group. - 1/6 studies found significant improvement in dietary outcomes in favour of the intervention compared with control group. - 2/4 studies found a clinically relevant overall increase in QoL at 6 weeks in favour of the intervention compared with control group.   1/4 studies found a significant difference in depression and anxiety scores at 6 weeks, decreasing more in the intervention group. | *Quantitative analysis: Not reported*  *Other relevant findings:*   - Personalization may be important (6 studies reported significant positive between-group effects) | - 1.1 Goal setting (behavior) (n=6), - 1.4 Action Planning (n=6) - 2.2 Feedback on behavior (n=6), - 7.1 Prompts/cues [email reminders and/or motivational SMS] (n=4) - 10.3 Non-specific rewards (n=1) - 6.1 Demonstration of behavior role modelling (n=1) | *Cochrane Collaboration’s Risk of Bias:*  Overall risk of bias was relatively high. | **Moderate**  More than 1 noncritical weakness  (No pre-registration, no justification for selection of study design, no information on funding of individual studies) |
| Athilingam 2018 [29]  Integrative review | mHealth  Commercially available mobile apps specifically for heart failure self-care | Searches:  2008-2017  Publications:  *Noy reported* | 18 studies  RCT or quasi-experimental design or a pre-post-test design | Clinical population: heart failure patients  N= 847 | Heart failure outcomes including QoL, readmission, knowledge, self-care practice | *Main effects: Not reported*  Other relevant findings:   - 8/18 studies reported a trend or significant reduction in heart failure-related readmission - 7/18 studies reported improved self-management - QoL was measured by most studies; all of which reported a trend for significant improvement   3 studies reported improved heart failure knowledge | *Quantitative analysis: Not reported* | - 2.4 Self-monitoring outcomes of behavior [weight and symptoms] (n=14) - 7.1 Prompts/cues (n=8) - 5.1 Information about health consequences [heart failure education] (n=7) - 4.1 Instructions on how to perform a behavior [messaging on heart failure self-management] (n=4) | *Cochrane Collaboration’s Risk of Bias:*  Most studies were underpowered and had high bias across all categories indicating varying ranges of methodological rigor. | **Low**  One critical flaw (no pre-registered protocol)  More than one non-critical weakness (excluded studies not listed, no explanation for selection of study designs, study selection and data extraction not done in duplicate, insufficient detail on included studies, no information on funding sources) |
| Pfaeffli Dale 2015 [32]  Systematic review | mHealth  Any type of mobile phone capable of receiving SMS, websites, web applications | Searches:  Inception – 2015  Publications:  2012-2015 | 7 studies described in 9 papers  Experimental (RCT) or quasi-experimental trials | Clinical Population: Patients of any age with any type of established CVD (coronary heart disease,  Acute coronary syndrome, heart failure, stroke, congenital heart disease)  N= 1,236 | Behaviour change including physical activity, diet, smoking, alcohol, medication adherence | *Main effect: Not reported*  *Other relevant findings:*   - 5/7 studies found a positive treatment effect on behavior change. - PA: 2/2 studies on PA reported an increase in self-reported PA and walking compared to control or usual care, but not MVPA or sitting. - Medication adherence: 3/3 studies found a treatment effect in favour of the intervention compared to usual care or control. - No treatment effects were observed on dietary habits and alcohol or smoking cessation. | *Quantitative analysis:*  *Not reported* | - 5.1 Information about health consequences (k=5) - 2.3 Self-monitoring of behavior (k=4) - 7.1 Prompts/cues (k=4) - 1.1 Goal setting (behavior) (k=3) | *Cochrane Collaboration’s Risk of Bias:*   - All studies had either unclear or high risk of bias for one or more domains. - High risk of bias was most common for blinding of participant, personnel or assessors and selective outcome reporting. - Jadad score for study quality: studies scored between 2-5 out of a possible 5. | **Low**  One critical flaw (no pre-registered protocol)  More than one non-critical weakness (no justification for selection of study design, no information on funding sources, no discussion of heterogeneity) |
| Khoong 2021 [37]  Systematic review and meta-analysis | mHealth  Text messaging and mobile apps | Searches:  2005-2019  Publications:  2011-2019 | 25 studies reported in 29 papers  RCTs, pilot RCTS | Clinical population: patients that have lower educational attainment, are older, or are persons of color    N=5,006 | Blood pressure | *Main effects, intervention versus control:*   - Meta-analysis of 7 trials with 6 month follow up showed no between group difference in SBP   *Other relevant findings:*   - SPB significantly decreased in the intervention group at 6 months (MD= −4.10; 95% CI: −6.38 to −1.38) but did not significantly change in the control group (MD= -2.53; 95% CI: −8.25 to 3.18) | *Quantitative analysis: Not reported* | - 4.2 Information about antecedents (k=20) - 4.1 Instruction on how to perform a behavior (k=15) - 6.1 Demonstration of the behaviour (k=15) - 2.4 Self-monitoring outcomes of behaviour (k=9) - 7.1 Prompts/cues (k=9) - Human coaching (k=11) - Tailoring (k=13) | *GRADE:*   - Only two studies were rated as high quality. - Several studies were considered lower quality because of issues related to bias (not an RCT), indirectness (intervention included many components beyond mHealth), or imprecision (small sample size; wide confidence interval). | **Critical Low**  More than one critical flaw (appropriateness of meta-analytic method unclear, insufficient assessment of risk of bias, no consideration of bias when interpreting results, no assessment of publication bias)  More than one non-critical weakness (missing information on inclusion criteria for comparator groups, no justification for selection of study design, study selection not done in duplicate, excluded studies not listed, no information on funding sources, no assessment of impact of risk of bias, no discussion of heterogeneity) |
| Duff 2017 [30]  Systematic review | eHealth and mHealth  Computer, mobile phone, tablet, or phone (e.g., mobile phone app, emails, text messages, and phone calls). | Searches:  2000-2016  Publications:  2000-2015 | 23 studies  RCT and quasi-experimental studies | Clinical population: clinically diagnosed with CVD  N=3,633* | Physical activity | Main effect:  *Not reported*  Other relevant findings:   - 8/15 interventions that had PA as an outcome reported statistically significant improvements in PA between the experimental and control groups. - 5/15 interventions found no significant differences in PA between the experimental and control group - eHealth interventions were at par with or were significantly better than standard cardiac rehabilitation at improving PA levels of cardiac patients | *Quantitative analysis: Not reported* | *In the studies with improved PA:*   - 1.1 Goal setting (behavior) (75%) - 5.1 Information about health consequences (75%) - 2.2 Feedback on behavior (63%) - 4.1 Instruction on how to perform the behavior (63%) - 2.3 Self-monitoring of behavior (50%) - 3.2 Social support (practical) (50%) | *Cochrane Collaboration’s Risk of Bias:*   - Overall risk of bias was relatively high. | **Critical Low**  More than one critical flaw (no pre-registered protocol, no consideration of bias when interpreting results)  More than one non-critical weakness (excluded studies not listed, no information on funding sources, no discussion of heterogeneity) |
| **Diabetes** | | | | | | | | | | |
| Pal 2013 [42]  Systematic review and meta-analysis | eHealth  Computer-based software applications that respond to user input and aim to generate tailored content. | Searches:  Up to 2011  Publications:  1986-2011 | 16 studies reported in 20 articles  RCTs | Clinical population: Adult patients (aged 18 years or over) with type 2 diabetes mellitus  N= 3,578 | HbA1c, HRQoL | *Main effects, intervention versus comparator:*   - HbA1c: -2.3 mmol/mol (MD -0.2%; 95% CI -0.4 to -0.1; I^2^ = 58%; k=11) - HRQoL: Five studies reported health-related quality of life scores but none showed statistically significant between group differences.   Subgroup analysis   - The effect size on HbA1c was larger in the mobile phone subgroup MD HbA1c -5.5 mmol/mol or -0.5% (95% CI -0.7 to -0.3; k=3) | *Quantitative analysis: Not reported*  *Other relevant findings:*   - 2.4 Self-monitoring outcomes of behavior and 2.7 Feedback on outcomes of behaviour were the most commonly used BCTs in interventions that had a statistically significant impact on HbA1c. - 5.1 Information about health consequences, 1.1 Goal setting and 1.2 Problem solving were the most commonly used techniques in ineffective interventions. | *Not reported* | *Cochrane Collaboration’s Risk of Bias:*   - All of the included studies were RCTs but none were blinded. - Most of the studies had unclear or high risk of bias across domains. | **High**  No critical flaws  One non-critical weakness (no justification for selection of study design) |
| El-Gayar 2021 [43]  Meta-analysis | mHealth  Mobile application designed to run on a mobile device such as a phone or another mobile device such as a tablet or a watch | Searches:  2010-2020  Publications:  2011-2019 | 21 studies  RCTs | Clinical population: adult diabetes patients  N=1,920 | HbA1c | *Main effects, intervention versus standard care treatment:*   - HbA1c (MD −0.38, 95% CI − 0.50 to− 0.25; p<0.0001; I^2^=0%; k=21) | *Sub-group analysis:*   - Interventions supporting   1.4 Action planning, 11.3 Conserving mental resources, 2.4 Self- monitoring of outcome(s) of behavior,  3.2 Social support (practical), and 9.1 Credible source,  likely result in a reduction in HbA1c.   - 1.4 Action planning (p = 0.004) and 2.4 Self-monitoring of outcome (s) of Behavior (p = 0.03) were present in interventions reporting statistically significant reduction in HbA1c compared to the interventions not supporting these techniques. - The certainty of evidence was moderate for 1.4 Action Planning but low for 2.4 Self-monitoring of outcome (s) of Behavior | - 2.3 Self- monitoring of behavior (k=20) - 2.4 Self-monitoring of outcome(s) of behavior (k=20) - 2.5 Feedback on outcome(s) of behavior (k=18) - 4.1 Instruction on how to perform the behavior (k=16) - 9.1 Credible source (k=15) | *Cochrane Collaboration’s Risk of Bias:*   - All studies had high risk of performance bias, but due to the nature of the studies, blinding of participants and personnel was not possible - All studies had low risk of detection, attrition, and reporting bias. - Some studies had unclear risk of selection bias. - Egger’s test did not indicate the presence of publication bias (p = 0.41)   *GRADE:*   - The collective body of evidence regarding the use of mobile apps for diabetes self- management was rated as high quality - However, when considering the use of theory and support for BCTs, the quality ranged from moderate to very low | **Low**  One critical flaw (no pre-registered protocol)  More than one non-critical weakness (excluded studies not listed, no justification for selection of study design, data extraction not done in duplicate, no information on funding sources) |
| Hadjiconstantinou 2016 [44]  Systematic review and meta-analysis | eHealth  Web--based/online intervention, with a combination of other modes, such as telephone calls or SMS (short message service) texts, that provided information, education, peer support, and/or overall therapeutic components | Searches:  1995-2016  Publications:  2000-2015 | 16 studies  RCTs | Clinical population: Adults with type 2 diabetes  N=3,612 | Wellbeing | *Main effect: Not reported*  *Other relevant findings:*   - Depression: 5 studies reported outcome data for depression. The pooled mean difference between the intervention and control arms on depression score was not significant (MD= -0.31; 95%CI -0.73 to 0.11; P=.15; I^2^= 89%).   Distress: 6 studies reported outcome data for distress. The pooled mean difference between the intervention and control arms on distress scores was not significant (MD= -0.11; 95%CI -0.38 to 0.16; P=.43; I^2^=87.7%). | *Quantitative analysis: Not reported*  *Other relevant findings:*   - 4 studies that had significant improvement in distress or depression shared some common characteristics, that is, the interventions combined synchronous and asynchronous communication, with the intervention running between 2 and 6 months. Providers were mostly psychologists, and studies including peer support were moderated. | - 5.1 Information about health consequences (k=14); - 2.3 Self-monitoring of behavior (k=12) - Providing motivation (k=12) - providing feedback (n=9); - 1.1 Goal setting (behavior) (k=9) - 1.2 Problem solving (k=9) - 1.4 Action planning (k=7) - 3.1 Social support (unspecified) (k=7) - Emotional control training (n=6) - 1.5 Review of behavioral goals (n=1). | Jadad scale:   - The methodological quality of the studies was generally high. - Nevertheless, some aspects, such as intention-to-treat, single-blinding, and sample size calculation, were not clearly reported in some studies. - The funnel plot and Egger’s test (P=.60) showed no obvious publication bias for studies reporting depression outcomes. - Egger’s test (P=.98) showed some indication of publication bias for studies reporting distress outcomes. - The funnel plot suggests some studies with a small negative SMD have not been reported. This suggests that the pooled mean may have been biased towards studies showing no effect or that control is preferable to intervention. | **Moderate**  No critical flaws  More than one non-critical weakness (no justification for selection of study design, excluded studies not listed, no information on funding of individual studies, no assessment of impact of risk of bias). |
| VanVugt 2013 [38]  Systematic review | eHealth  Online self-management programs for T2DM including online synchronous and asynchronous communication or telephone contact with patients. | Searches:  1994 -2012  Publications:  2002-2012 | 8 studies reported in 13 articles  RCTs | Clinical population: patients with type 2 diabetes mellitus  N=3,813 | Health behaviours including physical activity, diet, | *Main effect: Not reported*  *Other relevant findings:*   - 7/13 RCTs reported statistically significant improvements in health behaviors (diet, physical activity/exercise, medication use, smoking). - 9/13 articles reported statistically significant improvements in clinical outcomes measures, such as glycated hemoglobin (HbA1c), fasting BG, cholesterol, and triglycerides. - 9/13 articles reported statistically significant improvements in psychological outcomes, such as depression, diabetes distress, psychosocial well-being, self-efficacy, stress, and communication. | *Quantitative analysis: Not reported*  *Other relevant findings:*  The BCTs linked to improvements in health behaviours, clinical outcome measures, and psychological outcomes:   - 2.2 Feedback on behaviour - 5.1 Information about health consequences - 1.2 Problem solving - 2.4 Self-monitoring of outcomes of behaviour - 2.3 Self-monitoring of behavior - 3.1 Social support (unspecified)   The BCTs linked to improvements in clinical outcome measures:   - Goal setting (behaviour)   The BCTs linked to improvements in psychological outcomes:   - 6.2 Social comparison | - 2.2 Feedback on behaviour (n=8) - 5.1 Information about health consequences (n=7) - 1.2 Problem solving (n=7) - 2.4 Self-monitoring of outcomes of behaviour (n=6) - 2.3 Self-monitoring of behavior (n=6). | - Study quality ranged from 50% to 83% using the quality assessment tool proposed by van Tulder et al (1997). - No studies were at low risk of bias of blinding to the care provider, the patient or the researcher. Few studies had long-term follow up measures or used intention-to-treat analysis. | **Critical Low**  More than one critical flaw (no pre-registered protocol, no consideration of risk of bias when interpreting results)  More than one non-critical weakness (missing information on inclusion criteria for comparator, excluded studies not listed, no information on funding sources, no discussion on heterogeneity) |
| Dening 2020 [39]  Systematic review | eHealth  Web-based interventions e.g., providing content on a website, content via email with text, videos, and links to various web resources. | Searches:  2013-2019  Publications:  2003-2018 | 5 studies  RCTs or pilot RCTs | Clinical population: type 2 diabetics  N= 1,056 | Diabetes risk factors including diet behaviour, HbA1c, weight, fasting blood glucose, waist circumference, triglycerides. | *Main effect: Not reported*  *Other relevant findings:*   - 4/5 studies reported significant improvements in dietary behavior, representing healthier food choices, improvements in eating habits, reductions in carbohydrates, added sugar, sodium, saturated fat and overall fat intake, and/or increases in dietary knowledge. - 3/5 studies found significant mean reductions for HbA1c ranging from –0.3% to –0.8%, and/or weight ranging from –2.3 kg to –12.7 kg, fasting blood glucose (–1 mmol/L), waist circumference (–1 cm), and triglycerides (–60.1mg/dL). | *Quantitative analysis: Not reported* | - 2.3 Self-monitoring of behaviour (n=4) - 2.2 Feedback on behaviour (n=4) | *Cochrane risk-of-bias tool for randomized trials (RoB 2):*   - One study was assessed to be of fair quality, while the remaining four were assessed to be of poor quality. | **Critical Low**  More than one critical flaw (excluded studies not listed, no consideration of bias when interpreting results)  More than one non-critical weakness (no justification for selection of study design, no information on funding sources) |
| Fu 2017 [40]  Systematic review | mHealth  NCD self-management - diabetes  Diabetes App in combination with other intervention components (e.g., website, decision support, health provider feedback, personal health record, Glucometer) | Searches:  2011-2017  Publications:  *Not reported* | 12 studies  RCT, quasi-experimental design, or pre-test and post-test | Clinical population: adults with type 2 diabetes  N=822* | HbA1c | *Main effect: Not reported*  *Other relevant findings:*   - Clinical effectiveness, measured by reductions in HbA1c, ranged from 0.15% to 1.9%. However, the reductions were only statistically significant in four studies where the app design provided the greatest interactive features (HbA1C reduction of 0.4–1.9%). | *Quantitative analysis: Not reported*  *Other relevant findings:*   - 4/12 studies reporting statistically significant HbA1c reductions included instant app messages from an algorithm database website, a primary care provider, or a dietician. - The apps provided 2.2 Feedback on behaviour [messages] and 7.1 Prompts/cues [alert reminders]. - Diabetes apps with less interactive features had less effect on HbA1c reductions. | *Not reported* | *Down and Black’s checklist:*   - Study quality scores ranged from 12-21. The score can range from 0-28 and scores less than 14 indicate below average quality. | **Critical Low**  More than one critical flaw (no pre-registered protocol, no consideration of bias when interpreting results)  More than one non-critical weakness (no explanation for selection of study designs, study selection and data extraction not done in duplicate, excluded studies not listed, no information on funding sources, no discussion of heterogeneity) |
| Connelly 2013 [41]  Systematic review | e- and mHealth  NCD self-management - diabetes  Digital interventions delivered through websites (9), mobile phones (3), CD-ROM (2), and computers (1), to promote physical activity in Type 2 diabetes management. | Searches: 1991-2013  Publications:  2003-2012 | 15 studies  RCTs | Clinical population: type 2 diabetics  N=4,567* | Physical activity, HbA1c | *Main effect: Not reported*  *Other relevant findings:*   - 9/15 studies reported a significant increase in PA compared to control (web-based, n=6; CD-ROM, n=2; computer learning based, n=1). - Of eight studies that assessed changes in HbA1c, significant decreases of 1.1-2% were found in two mobile phone interventions and 0.6% in two web-based interventions. | *Quantitative analysis: Not reported*  *Other relevant findings:*   - 3.1 Social support (unspecified) [personal coach], 2.3 Self-monitoring of behavior [logbooks], and reinforcement strategies such as phone calls and email counselling were found to be effective components for behavior change. - 3.1 Social support (unspecified) [peer support] was found to have no effect on physical activity, suggesting it was less helpful than a personal coach. - Reinforcement through an email peer support system did not improve outcomes and participants were more satisfied with personal email counselling from the study counsellor. | *Not reported* | *Tool from van Tulder et al.:*   - 6/15 articles scored at least nine, deeming them of high methodological quality. | **Critical Low**  More than one critical flaw (No pre-registered protocol, unsatisfactory risk of bias assessment, no consideration of bias when interpreting results)  More than one non-critical weakness (excluded studies not listed, study selection and data extraction not done in duplicate, no information on funding sources, no discussion of heterogeneity) |
| **Cancer** | | | | | | | | | | |
| Furness 2020 [47]  Systematic review and meta-analysis | eHealth and mHealth    Synchronous modes included telephone, Skype, and videoconferencing.  Asynchronous modes used combinations of custom or existing websites and mobile apps, with short messaging and email.  Studies that used combined methods used web-based intervention and an online moderated forum, and telephone and SMS text messaging. | Searches:  2007-2019  Publications:  *Not reported* | 24 studies  RCTs | Clinical population: cancer survivors post-treatment (n=17), patients during active cancer treatment (n=6), patients under surveillance (n=1).  N=4,583 | Physical activity, diet, mental wellbeing | *Main effects, intervention versus control:*   - PA (SMD 0.34; 95% CI 0.21 to 0.48; I^2^ =49%; k=15). - Diet (SMD 0.44; 95% CI 0.18 to 0.70; I^2^=77%; k=6). - Anxiety (SMD 1.21; 95% CI 0.36 to 2.07; I^2^=96%; k=4). - Depression (SMD 0.15; 95%CI 0.00 to 0.30; I^2^=27%; k=5). - No significant between group effect for QoL or fatigue.   *Other relevant findings:*   - Of the studies that provided change scores comparing synchronous interventions with usual care, there was a very small but non-significant favorable intervention effect on anxiety (SMD 0.15; 95% CI −0.09 to 0.40; I^2^=0%; k=2). | *Quantitative analysis: Not reported*  *Other relevant findings:*   - When analyzed by delivery method, the intervention delivery mode of synchronous or asynchronous did not impact the overall positive outcome (PA, nutrition, QoL, fatigue, depression or anxiety) effect. | - 1.1 Goal setting (behavior) (87%) - 2.3 Self-monitoring of behavior (85%) - 5.1 Information about health consequences (70%) - 1.2 Problem solving (67%) - 1.4 Action planning (62%) - 2.2 Feedback on behavior (61%) - 4.1 Instructions on how to perform behavior (61%) | *Cochrane Collaboration’s Risk of Bias:*   - One study had low risk of bias. - There was a high degree of risk associated with blinding of personnel, participants, and outcome assessment. - The reporting of study-related processes was highly variable, which led to many areas of the risk of bias being assigned as unclear. | **Low**  One critical flaw (no assessment of publication bias)  More than one non-critical weakness (no explanation for selection of study designs, excluded studies not listed, no information on funding sources, no assessment of impact of risk of bias, no discussion of heterogeneity) |
| Ester 2021 [45]  Systematic review | eHealth and mHealth  Mobile app, SMS text messages, wearable activity tracker, website, email, or other eHealth | Searches:  Inception to January 2021  Publications:  2005-2020 | 67 studies reported in 71 papers  RCTs, non-randomised single- or two-arm trials | Clinical population: patients with cancer  N=6,655 | Physical activity and wellbeing | *Main effects: Not reported*  *Other relevant findings:*   - Statistically significant postintervention improvements in PA behavior were reported in 52% (35/67; 18 between-group, 17 within-group) of interventions. - The remaining 32 interventions reported in no change (29/67, 43%), decreases in PA (1/67, 2%), or did not report on statistical significance (2/67, 3%). | *Quantitative analysis: Not reported*  *Other relevant findings:*  *Weight analyses* showed the greatest associations between   - increased PA levels and PA as a primary outcome (0.621) - interventions using websites (0.656) or mobile apps (0.563) - interventions integrating multiple behavioral theories (0.750) - interventions using BCTs of 1.2 Problem solving (0.657) and 1.4 Action planning (0.645) | - 2.3 Self-monitoring of behaviour (95%) - 9.1 Credible source (93%) - 1.1 Goal setting (behaviour) (90%) - 12.5 Adding objects to the environment (90%) - 3.1 Social support (unspecified) (83%) - Review behaviour goal (71%) - 2.2 Feedback on behaviour (66%) - 4.1 Instructions on how to perform a behaviour (66%) - 5.1 Information about health consequences (57%) - 8.1 Behavioural practice/rehearsal (53%) - 8.3 Habit formation (53%) - 1.2 Problem solving (52%) - 7.1 Prompts/cues (52%) - 1.4 Action planning (46%) | *Cochrane Collaboration’s Risk of Bias:*   - All studies had concerns with high risk of bias, mostly because of the risk of confounding, measurement bias, and incomplete reporting - The overall RoB among the 45 RCTs ranged from some risk (4/45, 8%) to high risk (41/45, 91%). - Because of the risk of confounding, 95% (21/22) of the nonrandomized studies were found to have critical RoB. RoB in the measurement of outcome was moderate (10/67, 15%) or serious (9/67, 13%) for most single-arm studies, whereas it remained low across other categories. | **Moderate**  No critical flaws  More than one non-critical weakness (missing information on inclusion criteria for comparator groups, excluded studies not listed, no information on funding sources) |
| Roberts 2017 [48]  Systematic review and meta-analysis | eHealth and mHealth  Online courses/modules, biweekly/weeks emails, workshops, email counselling, virtual walking environment, website, automated system, personalized education, nutrition/PA log, video demonstrations, text messages, PA manual. | Searches:  Inception to 2016  Publications:  2012-2016 | 15 studies  RCTs, NRCTs, one-arm pre-post comparison studies | Clinical population: Adults (≥18 years) who had a cancer diagnosis of any type  N=1,335* | Physical activity | *Main effects, intervention versus control:*   - MVPA (MD = 49 min per week; 95% CI 16 to 82, p=0.004; I^2^= 73%; k=5 RCT) - BMI/weight (SMD = −0.23; 95% CI −0.41 to −0.05, p=0.011; I^2^=0%; k=4).   *Other relevant findings:*   - Meta-analysis of dietary intake was not appropriate. One out of three RCTs reported a significant effect on dietary outcomes. - DBCIs resulted in a decrease in fatigue, but this was not significant (SMD = −0.23; 95% CI −0.51 to 0.05; p = 0.103; I^2^=78%; k=3). - Overall, there were no significant changes on cancer-specific QoL (MD = 0.61; 95%CI −1.83 to 3.06; p = 0.62; I^2^=42%). | *Quantitative analysis: Not reported* | - 2.3 Self-monitoring of behavior (n= 15) - 1.1 Goal setting (behavior) (n= 13) - 9.1 Credible source (n= 13) - 2.2 Feedback on behavior (n= 12) | *Cochrane Collaboration’s Risk of Bias:*   - No studies had a low risk of bias overall. - Study quality was deemed to be low for the majority of included studies.   A funnel plot suggested that there may be some indication of publication bias among smaller studies reporting MVPA in minutes per week. | **Moderate**  No critical flaws  More than one non-critical weakness (missing information on inclusion criteria for comparator, excluded studies not listed, no information on funding sources, impact of risk of bias not assessed) |
| Gitonga 2022 [49]  Systematic review and meta-analysis | eHealth  ‘Connected’ interventions (offering a two‐way communication in the flow and use of data) including web-based applications, smart applications, and wearable devices. | Searches:  up to 2021  Publications:  2012-2020 | 37 studies  RCTs, NRCTs, qualitative studies | Clinical population: Cancer patients  N=8,956 | Psychological wellbeing, QoL | *Main effects, intervention versus usual care:*   - Symptoms of depression (SMD -0.226; 95% CI -0.303 to -0.149; I^2^= 0%; k=7) - Symptoms of anxiety (SMD -0.188; 95% CI 0.279 to -0.0963; I^2^= 63.7%; k=7) | *Quantitative analysis: Not reported*  *Other relevant findings:*   - Mixed efficacy was found for CH interventions targeting psychosocial support and rehabilitation - Mixed findings for CH interventions offering symptom burden, symptom monitoring, and self‐management - CH‐mediated peer support and social networking interventions had overall promising efficacy on psychological and QoL outcomes - Mixed findings for health coaching and PA training. | - Psychoeducation and Information support (e.g. cognitive behavioral therapy) (k=26) - Psychosocial support and rehabilitation (k=18) - Symptom monitoring, reporting, self-management (k=18) - 3.1 Social support (unspecified) [peer and social support] (k=9) - Health coaching and PA training (k=3) | *Mixed Methods Appraisal Tool:*   - 14/37 studies met 4-5 criteria (high quality) - 23/37 studies met 2-3 criteria (moderate quality)   No significant publication bias was detected based on the insignificant Egger's test and through funnel plot examination | **Low**  One critical flaw (no consideration of bias when interpreting results)  More than one non-critical weakness (missing information on inclusion criteria for comparator groups, no explanation for selection of study designs, excluded studies not listed, no information on funding sources, no assessment of impact of risk of bias) |
| Qan'ir 2019 [46]  Systematic review | eHealth  Self‐guided websites; web‐based programs supported by medical professionals; and a mobile health app with clinical nurse supervision. | Searches: 2000-2018  Publications: | 10 studies  RCT or quasi‐experimental research design | Clinical population: patients with prostate cancer at different stage  N= 1,124* | Mental wellbeing, HRQoL | *Main effects: Not reported*  *Other relevant findings:*   - Anxiety: 1/5 studies reported a significant reduction in anxiety among technology-based intervention users (ANCOVA: CI= -4.131 to ‐2.763, d=.67, p≤.001) compared with patients receiving usual care. - Depression: 2/7 studies reported a significant improvement in depression in the technology-based intervention users compared with the participants in control conditions. - HRQoL: 3/8 studies indicated significant improvement in HRQoL among technology-based intervention users. | *Quantitative analysis: Not reported* | - 5.1 Information about health consequences (k=8) - 3.1 Social support (unspecified) (k=5) - Psychoeducation and cognitive behavioural therapy (k=4) - 9.1 Credible source (k=4) - 2.4 Self-monitoring of outcomes of behaviour (k=4) - 4.1 Instruction on how to perform the behaviour (k=3) - 1.2 Problem solving [coping] (k=3) - 9.2 Pros and cons (k=2) - 3w - 2.7 Feedback on outcomes of behaviour (k=1) - 7.1 Prompts/cues (k=1) - 11.2 Reduce negative emotions (k=1) | *Cochrane Collaboration’s Risk of Bias:*   - In general, there was a high risk of bias and underpowered sample sizes. - No studies were at low risk of bias overall. - All had various levels of risk of bias in different domains. | **Low**  One critical flaw (no pre-registered protocol)  More than one non-critical weakness (missing information on inclusion criteria for comparator, data extraction not done in duplicate, excluded studies not listed, no information on funding sources) |
| **Asthma** | | | | | | | | | | |
| Miller 2017 [50]  Systematic review and meta-analysis | mHealth  Mobile app or SMS platform. | Searches:  Up to 2016  Publications:  2005-2016 | 11 studies  RCTs | Clinical population: individuals with asthma  N=954 | QoL, medication adherence, asthma control | *Main effects, intervention versus standard treatment:*   - Medication adherence (*g* = 0.63; 95% CI 0.31 to 0.94; P<0.001) - QoL (*g* = 0.64; 95% CI 0.19 to 1.08; P=0.01) - mean percentage of unscheduled visits (data unclear) - well-controlled asthma (data unclear)   *Main effects, intervention versus paper-based:*   - No effects for medication adherence or symptom-monitoring adherence. | *Quantitative analysis: Not reported* | - 5.1 Information about health consequences (85%) - 2.3 Self-monitoring of behavior (62%) - 7.1 Prompts/cues (62%) - 4.1 Instruction on how to perform the behavior (54%) - 3.2 Social support (practical) (46%) - 2.7 Feedback on outcomes of behavior (46%) - 1.4 Action planning (31%) - 2.6 Biofeedback (15%) - 4.2 Information about antecedents (8%) - 6.1 Demonstration of the behavior (8%). | *Cochrane Collaboration’s Risk of Bias:*   - No studies had low risk of bias overall. - The majority had unclear risk of bias particularly for blinding of outcome assessment, allocation concealment and selective reporting. - Six studies had high risk of bias for incomplete outcome data. | **Low**  One critical flaw (no pre-registered protocol)  More than one non-critical weakness (no justification for selection of study design, study selection and data extraction not done in duplicate, no information on funding sources) |
| **Chronic Conditions** | | | | | | | | | | |
| Tong 2021 [51]  Systematic review, meta-analysis and meta-regression | mHealth  Personalized mobile app or fitness tracker | Searches:  2010-2020  Publications: 2010-2020 | 39 studies  RCTs, quasi-experimental studies | Clinical Population: Chronic conditions  N=77,243 | Lifestyle behavior outcomes including physical activity and sedentary behavior, diet, smoking, and alcohol consumption | *Main effects, intervention versus control:*   - Lifestyle behavior outcomes: moderate positive effect (SMD 0.663, 95% CI 0.228 to 1.10; k=14). | *Meta-regression:*   - Analyses of total number of BCTs in personalized features, or the number of personalized features were not statistically significant. | - 2.2 Feedback on behavior (k=26) - 2.3 Self-monitoring of behavior (k=24) - 1.1 Goal setting (behaviour) (k=19) | *Cochrane Collaboration’s Risk of Bias:*   - Risk of bias was assessed as low in 3/5 categories in 7 RCTs. - The Egger’s test had an intercept of 2.88 (95% CI 1.24–4.52, p-value = 0.002) indicating possible publication bias | **Moderate**  No critical flaws  More than one non-critical weakness (no justification for selection of study design, unclear whether data extraction was done in duplicate) |
| Liu 2020 [52]  Systematic review and meta-analysis | mHealth  Mobile app-assisted self-care interventions. | Searches:  2007-2019  Publications:  2008-2019 | 27 intervention arms reported in 24 papers  RCTs | Clinical population: patients with type 2 diabetes and/or hypertension  N=2,223* | Blood pressure, HbA1c, blood glucose, waist circumference, BMI, weight, cholesterol profile, lipid profile | *Main effects, intervention versus control:*   - HbA1c (SMD= −0.44; 95%CI −0.59 to −0.29; *P*<.001; I^2^=50; k=21), - SBP (SMD= −0.17; 95%CI −0.31 to −0.03; *P*=.02; I^2^=41; k=16) - DBP (SMD= −0.17; 95%CI −0.30 to −0.03; *P*=.02; I^2^=25; k=14). - Fasting BG (SMD = −0.29; 95% CI −0.49 to −0.10; P=.004; I^2^=2) corresponding to an absolute MD of −0.66 mmol/L (95% CI −1.06 to −0.26) - Waist circumference (SMD = −0.23; 95% CI −0.43 to −0.04; P=.02; I^2^=0) corresponding to an absolute MD of −1.62 cm (95% CI −2.84 to −0.40). - No effects for body weight, BMI, total cholesterol, LDL-C, HDL-C, and triglycerides.   *In sub-group analysis:*   - Mobile app-assisted interventions led to significant reductions in SBP in hypertensive patients (SMD= −0.28; 95%CI −0.51 to −0.04; *P*=.02), but not in diabetic patients (SMD= −0.08; 95%CI −0.29 to 0.13; *P*=.46).   No significant change in DBP was observed in either hypertensive patients (SMD= −0.20; 95%CI −0.47 to 0.08; *P*=.17) or diabetic patients (SMD= −0.12; 95%CI −0.28 to 0.04; *P*=.16). | *Subgroup analysis:*  More favourable effects were detected in interventions with the following features than those without:  SBP   - 2.4 Self-monitoring outcomes of behaviour [BP monitoring] - 7.1 Prompts/cues [reminders] - Education materials - 2.2 Feedback on behaviour [data visualization] - diet- and physical activity–monitoring features was not associated with reductions in SBP   SBP and DBP   - 1.1 Goal setting (behavour) - 2.7 Feedback on outcomes of behaviour [data visualization]   HbA1c   - 2.3 Self-monitoring of behaviour [medication monitoring] - 9.1 Credible source [communication with health care providers] | *Not reported* | *Cochrane Collaboration’s Risk of Bias:*   - All studies were at high risk of bias for blinding of participants. - Most of the studies had unclear or high risk of bias across domains.   GRADE:   - SBP=moderate - DBP=moderate - Fasting BG = moderate - Waist circumference= moderate - Body weight = moderate - Total cholesterol = moderate - LDL-C = moderate - HDL-C= moderate - Triglycerides = moderate - HbA1c = low - BMI = low | **Critical Low**  More than one critical flaw (no pre-registered protocol, no consideration of bias when interpreting results)  More than one non-critical weakness (excluded studies not listed, no information on funding sources, no assessment of impact of risk of bias) |
| Liu 2013 [53]  Meta-analysis | eHealth  Internet. Some interventions had supplemental components that were not Internet-based, such as text messages, in-person visits, and live support. | Searches:  Up to 2012  Publications:  2004-2012 | 13 studies  RCT or case-control studies | Clinical population: Individuals with hypertension, obesity, diabetes, or post-menopausal women  N=2,221 | Blood pressure | *Main effects, intervention versus control:*   - SBP (SMD= -0.27; 95% CI -0.44 to-0.10; P=0.002; I^2^=61%; k=13) - DBP (SMD= -0.17; 95% CI; -0.33 to -0.01; P=0.03; I^2^=57; k=13).   Effect sizes translate to a decrease in SBP of 3.8 mmHg (95% CI 5.63 to 2.06) and DBP of 2.1 mmHg (95% CI 3.51 to 0.65) | *Subgroup analysis:*  Larger overall effects were observed for trials with 5 or more BCTs on:   - SBP (ES= -0.46; 95% CI -0.60 to-0.33) - DBP (ES= -0.31; 95% CI -0.44 to -0.18)   *Other relevant findings:*  BCTs that were used in more than 50% of the successful Internet-based interventions included the following:   - 5.1 Information about health consequences - 2.2 Feedback on behavior (86%) - 2.3 Self-monitoring of behaviors (71%) - 4.1 Instructions on how to perform a behavior (71%) | - *Not reported* | *Not reported*  No publication bias was observed for SBP (Begg test, P=0.71); however, the Begg test was significant for DBP (P=0.04). The funnel plot for DBP indicates a bias toward studies that had large standard errors, and studies with null findings tended to be missing. | **Critical Low**  More than one critical flaw (no pre-registered protocol, no risk of bias assessment, no consideration of bias when interpreting results)  More than one non-critical weakness (no justification for selection of study design, study selection not done in duplicate, excluded studies not listed, no information on funding sources, no assessment of impact of risk of bias, no discussion of heterogeneity) |
| Cucciniello 2021 [54]  Systematic Review | mHealth  Smartphone apps | Searches:  2008-2019  Publications:  2009-2019 | 69 studies  RCTs, pilot RCTs, cluster-randomized, individual cross-over design | Clinical population:  Patients with chronic conditions (diabetes, cardiovascular diseases, chronic respiratory diseases, and cancer)  N=7,993* | Physiological and clinical outcomes including HbA1c, medication adherence | *Main effects: Not reported*  *Other relevant findings:*   - Cardiac outcomes: 3/3 studies reported positive outcomes. - Endocrine outcomes: 7/20 studies reporting significant differences in HbA1c between groups at follow-up. 13/20 reported neutral results. - General outcomes: 1/1 study reported positive outcomes - Metabolism and nutrition outcomes: 1/1 study reported positive outcomes - Physical functioning: 2/7 studies reported positive outcome. 5/7 studies reported neutral results. - Global quality of life: 2/5 studies reported positive outcome. 3/5 studies reported neutral results. - 5/7 studies assessing the impact on medication adherence reported significant outcome improvements. 2/7 studies reported neutral results. | *Quantitative analysis: Not reported* | - 4.1 Instruction on how to perform a behaviour (k=49, 71%) - 2.7 Feedback on outcomes of behaviour (k=41, 59%) - 3.1 Social support (unspecified) (k=39, 57%) - 12.5 Adding objects to the environment (k=39, 57%) - 2.4 Self-monitoring of outcome(s) of behaviour (k=38, 55%) | *Revised Cochrane risk-of-bias tool for randomised trials (RoB 2):*   - Sixty-four (93%) studies presented an overall high risk of bias per the Cochrane RoB 2 tool, whilst only five had a low risk for at least four domains - The main issue was the plausible impossibility to blind study participants to the intervention, together with potential deviations from the intended interventions | **Low**  One critical flaw (no pre-registered protocol)  More than one non-critical weakness (excluded studies not listed, data extraction not done in duplicate, no information on funding sources, no discussion of heterogeneity) |
| **Lifestyle behaviours** | | | | | | | | | | |
| Newby 2020 [55]  Systematic review and meta-analysis | eHealth  Interactive voice response, automated text, email, website, DVD. | Searches:  Inception to 2018  Publications: 2001-2018 | 20 studies  RCTs | General population  N= 5,624 | Self-efficacy | *Main effects, intervention versus control/usual care:*   - Self-efficacy (g = 0.190, CI 0.078 to 0.303; I^2^=69.8%). - Following removal of studies presenting a high risk of bias, the effect on self-efficacy remained significant (g = 0.211, CI 0.092 to 0.329). | *Moderator analysis*   - Including the BCT 5.3 Information about social and environmental consequences led to a small negative effect on self-efficacy (g= -0.297; Q=7.072; p= 0.008). - Interventions with the BCT 5.3 Information about social and environmental consequences had a lower effect (g = −0.029, CI − 0.222 to 0.164) than interventions without this BCT (g = 0.268, CI 0.165 to 0.372). - No other BCTs had a significant effect on self-efficacy. | - 1.1 Goal setting (behavior) (k=11) - 4.1 Instruction on how to perform the behavior (k=11) - 2.3 Self-monitoring of behavior (k=9) - 1.2 Problem-solving (k=9) - 1.4 Action planning (k=5) - 5.1 Information about health consequences (k=5) - 2.2 Feedback on behaviour (k=5) | *Cochrane Collaboration’s Risk of Bias:*   - One study had a low risk of bias, seven had a moderate risk of bias, and 12 had a high risk of bias. - The domain contributing most frequently to a high risk of bias rating was ‘selective reporting’. - Other domains frequently contributing high ratings included ‘random sequence generation’ and ‘allocation concealment’.   Examination of the funnel plot for self-efficacy and Begg’s test (p = .223) identified no evidence of publication bias. Publication bias was, however, indicated by Egger’s test (p = .018) | **Low**  One critical flaw (publication bias examined but not reported or discussed)  More than one non-critical weakness (data extraction not done in duplicate, excluded studies not listed, no information on funding sources, no discussion of heterogeneity) |
| Yang 2020 [56]  Meta-analytic review | eHealth  Web-based interactive health interventions with or without other type of media or face-to-face meetings. | Searches:  unclear  Publications:  2002-2016 | 67 studies  RCT, quasi-experiment, or within-subject pre-post designs | General population: general healthy adults or at-risk population  N=47,741 | General health /Health behaviors | *Main effects, intervention versus control:*   - Health mediating variables (weighted mean effect size d=0.29; 95% CI .20 to .38; p<.001; *k*=49), - Health behaviors (weighted mean effect size d=0.28; 95% CI .18 to .38; p<.001; *k*= 52) - Health outcomes (weighted mean effect size d=0.32; 95% CI .21 to .42; p<.001; *k=* 40).   *Moderator analysis:*   - Substance abuse (d=0.38; 95% CI .21, .56; p<.001) - Mental health (d=0.57; 95% CI .36 to .77; p<.001), - Nutrition, PA and overweight (d=0.29; 95% CI .19 to .40; p<.001) - Cancer (d=0.12; 95% CI .06 to .19; p<.001) - Chronic diseases (d=0.29; 95% CI .19 to .39; p<.001) - Health interventions with interactive features are effective in improving mediating variables, behaviors, and outcomes in a consistent manner. I^2^ =90.27% indicated large between-study variance. | *Moderator analysis:*  Significantly enhanced effectiveness was observed for interventions with the following features compared with those without:   - A mentor or coach (d = .63, 95% CI .37 – .88, p<.001) - Source interactivity (i.e., tailoring or personalization of the content based on the users’ input) (d = .35, 95% CI .29 to .42, p<.001)   The frequency of intervention messages had a moderating effect:  Daily messaging was more effective (d =.79, 95% CI .41–1.17, p<.001), versus one-shot messages (d =.30, 95% CI .15 to .44, p<.001) or continuously accessible message or system(s) (d =.22, 95% CI .16 – .28, p<.001). | - *Not reported* | *Not reported*  There was no evidence of publication bias. | **Critical Low**  More than one critical flaw (no pre-registered protocol, no risk of bias assessment, no consideration of bias when interpreting results)  More than one non-critical weakness (missing information on inclusion criteria for population and comparator, no justification for selection of study design, excluded studies not listed, insufficient details on included studies, no information on funding sources, impact of risk of bias not assessed, conflicts of interest not described) |
| McMahon 2020 [57]  Systematic review and meta-analysis | eHealth  Mobile phones, computers, laptops, tablets. | Searches:  Up to 2020  Publications:  2003-2015 | 9 studies  RCTs | General population: Males  N=1,329* | Lifestyle behaviors | *Main effects, intervention versus control/comparison:*   - BMI: -0.64 kg/m^2^ (Z=-2.75, p=0.01, I^2^ =76%; k=7). - Body weight: -2.27 kg (Z= −3.25, p<0.01, I^2^ =77%; k=8). - Waist circumference: -2.46 cm (Z= −2.30, p=0.02, I^2^=85%; k=6). - SBP: -4.22 mmHg (Z= −3.57, p<0.01, I^2^=0; k=6) - DBP = -2.87 mmHg (Z= −3.56, p<0.01, I^2^=0; k=6) | *Quantitative analysis: Not reported* | - 2.2 Feedback on behavior (k=7) - 1.1 Goal setting behaviour (k=8) - 2.3 Self-monitoring of beahviour (k=7) - 3.1 Social support (unspecified) (k=5) | *Cochrane Collaboration’s Risk of Bias:*   - Overall, risk of bias across the included trials was rated as low. - Three trials had unclear risk of bias and one trial had high risk of bias. | **Low**  One critical flaw (publication bias examined but not reported or discussed)  More than one non-critical weakness (no justification for selection of study design, excluded studies not listed, no information on funding sources) |
| Webb 2010 [66]  Systematic review and meta-analysis | eHealth  The primary components of the intervention must have been delivered via the Internet (not including CD-ROMs, SMS messaging, or other computer applications). | Searches: Inception -2008  Publications: 2000-2008 | 85 studies  RCTs | Not reported  N=43,236 | Health behaviors | *Main effects, intervention versus control:*   - The weighted average effect size across all interventions was small (d= 0.16; 95% CI 0.09 to 0.23; k=85). - PA (d= 0.24; 95% CI 0.09 to 0.38; k = 20) - Dietary behavior (d= 0.20; 95% CI 0.02 to 0.37; k = 10) - Alcohol consumption (d= 0.14; 95% CI 0.00 to 0.27; k = 9). - Interventions that targeted smoking abstinence tended to have slightly smaller effects on behavior that did not reach statistical significance (d= 0.07; 95%CI -0.04 to 0.18; k = 12). - 7Interventions that targeted multiple behaviors tended to have slightly smaller effects on behavior (d= 0.12; 95% CI 0.08 to 0.17; k = 10) than did interventions that targeted a single behavior (d= 0.17; 95% CI 0.09 to 0.24; k = 75), although both effects were statistically significant. | Significant effect on behaviour were observed for interventions that provided:   - 11.2 Reduce negative emotions [stress management] (d= 0.50, 95% CI 0.27 to 0.72; k=5) - General communication skills training (d= 0.49, 95% CI 0.25 to 0.73; k=3).   The following BCTs had effects on behavior that exceeded d= 0.20 (Median d= 0.28):   - 6.1 Demonstration of the behaviour - 1.2 Problem solving - 6.2 Social comparison - 1.1 Goal setting (behaviour) - 1.4 Action planning - 2.2 Feedback on behaviour   Small, but significant, effects on behavior were observed for interventions that provided:   - Automated tailored feedback (d= 0.18, k = 18, 95% CI 0.07 to0.28) - Enriched information environment (d= 0.15, k =30, 95% CI 0.07 to 0.23). - Access to an advisor (d= 0.29, k = 23, 95% CI 0.16 to 0.42), - Scheduled contact with an advisor (d= 0.22, k = 13, 95% CI 0.09 to 0.36) - Peer-to-peer access (d= 0.20, k = 20, 95% CI 0.09 to 0.21).   *Meta-regression:*   - the number of BCTs used had a significant positive impact on effect size (ß = 0.36, t = 3.48, P< .001). Interventions that used more techniques tended to have larger effects on behavior than did interventions that used fewer techniques. | - 5.1 Information about health consequences (k = 29) - 2.3 Self-monitoring of behavior (k = 28) - 1.2 Problem solving (k = 26) | *Not reported* | **Critical Low**  More than one critical flaw (no pre-registered protocol, no risk of bias assessment, no consideration of bias when interpreting results, no assessment of publication bias)  More than one non-critical weakness (missing information on inclusion criteria for population, no justification for selection of study design, study selection and data extraction not done in duplicate, excluded studies not listed, insufficient details on included studies, no information on funding sources, impact of risk of bias not assessed, conflicts of interest not described) |
| Free 2013 [60]  Systematic review and meta-analysis | mHealth  Mobile phones, PDAs, hand-held computers. | Searches:  1990-2010  Publications: 1990-2010 | 75 studies  RCT with parallel groups, cluster RCT, NRCTs | General population and clinical population.  N=17,538 | Objective measures of health or health service delivery, and subjective measures of health behaviours, disease management, health service delivery or use, and cognitive outcomes. | *Main effects, intervention versus comparison:*   - Smoking cessation interventions more than doubled biochemically-verified smoking cessation at 6 months ([RR] 2.16; 95% CI 1.77 to 2.62; p<0.0001; I^2^=3.6%; k=2) - Weight: There were no statistically or clinically significant changes in weight for trials using SMS to reduce calorie intake and increase physical activity (SMD -2.14kg; 95% CI -7.05 to 2.77 kg; I^2^=0%; k=not reported) or for trials using application software to reduce calorie intake (SMD 0.10; 95% CI -0.49 to 0.69 kg; I^2^=0%; k=not reported).   *Other relevant findings:*   - One diabetes self-management intervention reduced HbA1c (pooled MD -0.27; 95%CI -0.48 to -0.06; I^2^ =8.5%). - 6/12 studies reporting diabetes control outcomes showed statistically significant benefits. - Two trials found no change in BP outcomes. - 1/3 trials reporting lipid profile outcomes showed a statistically significant reduction. - One trial found no effect on weight loss. - For patients with asthma, one trial reported a statistically significant improvement in lung function. - For patients with hypertension, 1/2 studies showed a statistically significant reduction in BP. | *Quantitative analysis: Not reported* | - 2.2 Feedback on behavior (k=13) - 1.1 Goal setting behaviour (k=12) - 5.1 Information on health consequences (k=11) - Tailoring (k=11) - 2.3 Self-monitoring of behavior (k=10)   1.2 Problem solving (k=8) | *Cochrane Collaboration’s Risk of Bias:*   - Of 26 studies on health behavior change, 2 trials were at low risk of bias for all quality criteria. - Of 48 studies on disease management, 2 trials were at low risk of bias for all quality criteria. - No evidence of publication bias. | **Moderate**  No critical flaws  More than one non-critical weakness (no explanation for selection of study designs, no information on funding sources) |
| Milne-Ives 2020 [61]  Systematic review | mHealth  Mobile apps | Searches:  2014-2019  Publications: 2014-2019 | 52 studies  RCTs | Mixed: office workers, smokers; excessive drinkers, overweight and obese adults, general population, primary household cooks + children, CVD patients, students, primary care centre patients, participants with mild mental distress, underactive adults  N=not reported | Health behaviors | *Not reported*  *Other relevant findings:*   - Only 12/51 studies found that the app had a significantly better effect (P<.05) on participant health or behavior change outcomes than control or comparator groups - 16/51 studies found some evidence of effectiveness—whether there was a significant difference between the groups on some but not all of the outcomes, a significant difference over time but not between groups, or a significant improvement over the control only in a subgroup of the population. - The remaining 23 studies either found no significant difference between groups or effect on the primary outcome (22/51) or found that the app performed worse than the comparator (1/51). | *Quantitative analysis: Not reported*  *Other relevant findings:*   - There was mixed evidence for the BCTs used. - The most frequently used BCTs were all associated two to three times more with studies that found no significant effect compared with those that found a significant effect on the specified outcomes.   Only 4 BCTs had more significant evidence than not, but only by 1 study. These 4 BCTs were also only used in at most 2 apps:   - 1.6 Discrepancy between current behavior and goal - 4.2 Information about antecedents - 6.1 Demonstration of the behavior - 12.5 Adding objects to the environment | Only four BCTs were used in more than half of the apps:   - 1.1 Goal setting (behavior) (52%) - 2.2 Feedback on behavior (54%) - 2.3 Self-monitoring of behavior (72%) - 4.1 Instructions on how to perform the behavior (54%) | *Cochrane Collaboration’s Risk of Bias:* One study had a low risk of bias across all domains. The remaining studies had unclear or high risk of bias. | **Moderate**  No critical flaws  More than one non-critical weakness (excluded studies not listed, insufficient detail on included studies, no information on funding sources, no discussion of heterogeneity) |
| Dao 2021 [62]  Systematic Review | mHealth  Ecological-momentary intervention (EMI) provided in real time and in real world settings via a smartphone. | Searches:  2011-2020  Publications:  2011-2020 | 17 studies  RCTs, quasi-experimental studies | General population and clinical populations  N= 652 | Health behaviors including diet, physical activity, substance use, mental wellbeing | *Main effect: Not reported*  *Other relevant findings:*   - The 4 included RCTs reported nonstatistically significant improvements in substance abstinence, diet, weight loss, and sedentary time compared with the control group - Only 4 (24%) quasi-experimental studies reported statistically significant pre-post improvements in self-reported primary outcomes, namely depressive (*P*<.001; k=1) and psychotic symptoms (*P*=.03; k=1), drinking frequency (*P*<.001; k=1), and eating patterns (*P*=.01; k=1). | *Quantitative analysis: Not reported* | - 3.1 Social support (unspecified) (k=13, 76%); - 7.1 Prompts/cues (k=10, 59%) - 1.2 Problem solving (k=9, 53%) - 2.2 Feedback on behavior (k=6, 35%) - 2.3 Self-monitoring of behavior (k=7, 41%) - 3.1 Social support (emotional) (k=6, 35%) | *Cochrane risk of bias tool and Risk Of Bias In Non-randomized Studies of Interventions tool*:   - The risk of bias of the 4 RCTs was assessed as unclear for most of the risk of bias tool categories - Overall risk of bias in nonrandomized studies was assessed as serious for most studies | **Low**  One critical flaw (no consideration of bias when interpreting results)  More than one non-critical weakness (no explanation for selection of study designs, no information on funding sources, no discussion of heterogeneity) |
| Taylor 2022 [63]  Systematic review | mHealth  mHealth app intervention that included a virtual representation  (avatars and computerised agents to promote behaviour change, in the form of a coach, and as an interactive, customisable, image that provided participants with feedback) | Searches:  inception to 2021  Publications:  2017-2020 | 5 studies  RCTs, cross-sectional design | General population and clinical populations  N= 509 | Weight, diet, medication adherence, knowledge, self-care, exercise | *Main effect: Not reported*  *Other relevant findings:*   - 5/5 studies reported a significant change in weight loss, medication adherence, diet, exercise, knowledge, and self-care behaviours, with a greater behavioural change with either an avatar or an agent. - Weight Loss: One RCT showed significant weight loss in the avatar intervention group relative to the control group. - Exercise: Compared to baseline, participants significantly increased their weekly exercise volume (MET-mins/week = 0.618; SD = 0.119, p < 0.001) in response to the coach/goal recommendations. | *Quantitative analysis: Not reported* | - 2.3 Self-Monitoring of behaviour (k=4) - 9.1 Credible Source (k=4) - 13.1 Identification of self as role model (k=4) - 9.3 Comparative Future Outcomes (k=2) - 2.2 Feedback on Behaviour (k= 2) - 7.1 Prompts/Cues (k=2) | *Mixed Methods Appraisal Tool (MMAT):*   - All the included studies were of low or moderate quality - The lower scores were attributed to poor reporting of research design, unclear or inconsistent findings, therefore questioning the validity and reliability.   Of the 2 RCTs, the overall risk of bias was 50% moderate risk for 1 study and 50% low risk for the other study. | **Low**  One critical flaw (no pre-registered protocol)  More than one non-critical weakness (missing information on inclusion criteria for comparator, no justification for selection of study design, unclear whether data extraction was done in duplicate, excluded studies not listed, no information on funding sources, no discussion on heterogeneity) |
| Dugas 2020 [64]  Systematic review | mHealth  Apps, SMS, tracking with wearables in addition to information, supporting websites, health coaching, self-monitoring data, group counselling, education session. | Searches:  2007-2017  Publications:  2009-2017 | 21 studies  RCTs | Mixed: General population (32.2%); Clinical populations including patients with chronic conditions (e.g., HIV or Tuberculosis; 40.1%), or overweight and obesity (22.7%)  N=8,853* | Lifestyle behaviors | *Main effects: Not reported*  *Other relevant findings:*   - Evidence of mHealth effectiveness remains inconclusive: - 8/21 studies (44.4%) reported non-significant improvements in intervention arms compared to comparator arms - 6/21 (33.3%) reported significant improvements - 3/21 (16.7%) reported a significant difference for at least one, but not all, primary outcomes - 1/21 studies (5.6%) found that outcomes in the intervention arm were worse than those in the comparator arm. | *Quantitative analysis: Not reported*  *Other relevant findings:*  BCTs used in effective studies (k=6):   - 7.1 Prompts/cues (87.5%) - General personalization (50%) - 1.1 Goal setting (behavior) (37.5%) - 1.4 Action planning (37.5%)   BCTs used in ineffective studies (k=9):   - 2.3 Self-monitoring of behaviour (70%) - 3.1 Social support (unspecified) (60%) - 2.2 Feedback on behavior (50%) - 7.1 Prompts/cues (40%) | - 2.3 Self-monitoring behavior (50%), - 2.2 Feedback on behavior (47%), - Personalising intervention content based on performance (41%). | *Not reported* | **Critical Low**  (No pre-registered protocol, limited search strategy, no risk of bias assessment, and no consideration of bias when interpreting results)  More than one non-critical weakness (excluded studies not listed, no explanation for selection of study designs, study selection not done in duplicate, no information on funding sources, no discussion of heterogeneity) |
| Schoeppe 2016 [58]  Systematic review | mHealth  Stand alone apps or apps used in conjunction with physical education, parental education, counselling sessions, printed materials, motivational emails, websites, and pedometer use. | Searches: 2006-2016  Publications:  2010-2016 | 27 studies reported in 30 articles  RCT, NRCTs, randomised trials, pre-post studies | General population  N=2,699 | Diet, physical activity and sedentary behavior | *Main effect: Not reported*  *Other relevant findings:*   - App interventions showing significant between-group improvements in the behavioral and health outcomes tended to be multi-component interventions, with sample sizes above 90 participants and intervention durations longer than 8 weeks. - Of the 23 studies that targeted adults, 17 reported significant improvements in diet (n= 6), physical activity (n= 13), sedentary behavior (n=1), and other improved outcomes including weight status (n= 4), fitness (n= 1), blood pressure (n= 2) and cholesterol (n= 1). - Of the studies reporting significant findings, 11 studies detected significant between-group differences in diet (n=5), physical activity (n= 9) and weight status (n= 2) in favour of the app intervention group. - Seven studies found significant within-group improvements in diet (n=1), physical activity (n= 4), sedentary behavior (n= 1) and weight status (n=2), blood pressure (n= 2) and cholesterol levels (n= 1). - Five studies reported no significant changes in the health outcomes of interest, and no significant findings were found in relation to the outcome glucose levels (assessed in one study). | *Quantitative analysis: Not reported*  *Other relevant findings:*  BCTs that were part of efficacious apps included:   - 1.1 Goal-setting (behaviour) - 2.3 Self-monitoring of behaviour - 2.2 Feedback on behaviour   Some efficacious interventions also incorporated other BCTs, such as:   - Motivational messages (k=3) - Health education/tailored advice (k=6) - Reinforcement (k=4) - Gamification in the form of exergames, award and rewards (k=5) - 6.2 Social comparison [social support through interaction with peers (n=3) and friendly team challenges (k=4)]. | *Not reported* | *CONSORT checklist:*   - Overall, study quality ranged from high (k=11), to fair (k=8), and low (k=8). - Most of the 13 interventions that used an app in combination with other intervention strategies were of high quality (k=9), whilst most of the 14 stand-alone app interventions were of fair (k=6) or low quality (k=6). - Fewer studies reported sample size calculations and included randomisation and blinding procedures in their study design. | **Critical Low**  More than one critical flaw (no pre-registered protocol, inadequate assessment of risk of bias, no consideration of bias when interpreting results)  More than one non-critical weakness (missing information on inclusion criteria for comparator, no justification for selection of study design, excluded studies not listed, no information on funding sources, no discussion on heterogeneity) |
| Aalbers 2011 [59]  Systematic review | eHealth  Mainly web-based whereby the user had direct access and could interact with the intervention instruction program on the worldwide web, without the need of installing any software | Searches: 1995-2010  Publications:  2002-2010 | 10 studies reported in 12 papers  RCTs and NRCTs | General population: Adults aged 50 and older, community dwelling without major physical or cognitive disabilities  N=4,984 | Lifestyle behaviours | *Main effect: Not reported*  *Other relevant findings:*   - Average effect size (Cohen's d): from 7 studies for the online interventions in comparison to the offline control 0.19 (±0.21) and online control groups 0.39 (±0.37). | *Quantitative analysis: Not reported* | - Social network forums (k=7) - 2.3 Self-monitoring of behaviour (k=6) - 1.1 Goal setting (behaviour) (k=5) - 2.2 Feedback on behavior (k=3) - 7.1 Prompts/cues (k=2) | *2009 Cochrane EPOC (Evidence Practice and Organization of Care Group) form:*   - No studies scored positive on all quality criteria. - Most studies lacked a concise description on the sequence generation in randomization (n=6), allocation concealment (n=7), and protection against contamination (n=6). | **Critical Low**  More than one critical flaw (no pre-registered protocol, limited search strategy)  More than one non-critical weakness (excluded studies not listed, no information on funding sources, no information on conflict of interest) |
| Thomas Craig 2020 [65]  Systematic review | eHealth and mHealth  Automated digital health technologies (i.e., mHealth, eHealth, telemedicine, internet-based platforms, Internet of Things (IoT) sensors/wearables, etc.) | Searches:  2013-2020  Publications:  2013-2020 | 30 studies reported in 33 papers  RCTs, single-arm observational studies | General population and clinical populations  N= 4,778* | Health behaviors including physical activity, diet, sun protection, medication adherence, | *Main effect: Not reported*  *Other relevant findings:*   - Few studies reported associated changes in health (e.g., limited to clinical and intermediate) outcomes following digital behavior change interventions. - Weight loss varied, with a range of mean weight loss (1.44 – 3 kg) reported across six studies. - Three studies in prediabetic or diabetic patients reported a range of decreased HbA1c (−0.5% to −0.1%) at endpoint. | *Quantitative analysis: Not reported* | - 1.Fedback and monitoring (k=27) - Shaping knowledge (k=23) - Associations (k=23) - Goals and planning (k=21) - Social support (k=11) - Reward and threat (k=8) - Regulation (k=7) - Natural consequences (k=6) - Comparison of behavior (k=5) - Repetition and substitution (k=5) - Comparison of outcomes (k=3) - Scheduled consequences (k=3) - Antecedents (k=2) - Self-belief (k=1) | *Oxford*  *Levels of Evidence:*   - 16 trials provided Level 1b evidence; 13 studies were assessed as Level 2b; and 4 observational studies provided Level 4 evidence. - Reasons for downgrading study quality included selection bias, high loss to follow-up, and lack of intent-to-treat analysis. | **Critical Low**  More than one critical flaw (no risk of bias assessment, no consideration of bias when interpreting results)  More than one non-critical weakness (no justification for selection of study design, excluded studies not listed, no information on funding sources) |
| **Weight management** | | | | | | | | | | |
| Antoun 2022 [72]  Systematic review and meta-analysis | mHealth  Mobile apps, trackers, social media. | Searches: Inception to January 2022  Publications: 2011-2021 | 34 studies reporting 50 intervention arms  RCTs | General population  N=4,084* | Weight loss | *Main effects, intervention versus control:*   - Weight loss at 3 months: –1.99 kg (95% CI –2.19 to –1.79 kg; *I*^2^=81%) - Weight loss at 6 months –2.80 kg (95% CI –3.03 to –2.56 kg; *I*^2^=91%) | *Subgroup analysis*   - There was no association between weight loss and any specific type or number of app features.   *Subgroup analysis: Type of intervention accompanying the mobile app*  The combination of the mobile app, tracker, and behavioural interventions showed a statistically significant weight loss of:   - –2.09 kg (95% CI –2.32 to –1.86 kg; *I*^2^=91%) at 3 months - –3.77 kg (95% CI –4.05 to –3.49 kg; *I*^2^=90%) at 6 months   *Subgroup analysis:*  *Type of behavioural intervention; human-based versus passive*  Only the combination of the mobile app with intensive behaviour coaching or feedback by a human coach showed a statistically significant weight loss of:   - –2.03 kg (95% CI –2.80 to –1.26 kg; *I*^2^=83%) at 3 months –2.63 kg (95% CI –2.97 to –2.29 kg; *I*^2^=91%) at 6 months | - 2.4 Self-monitoring outcomes of behaviour (intervention arms=50; 100%) - 5.1 Information about health consequences [education] (arms=15; 30%) - 2.2 Feedback on behaviour or 2.7 Feedback on outcomes of fbeahviour (arm=20; 40%) - 3.1 Social support (unspecified) (arms=12; 24%) - 10.1 Reward outcome (arms=7; 14%) - Gamification (arms=3; 6%) | *Cochrane Collaboration’s Risk of Bias:*   - One-third of the articles had a high risk of bias; however, this bias could not have been avoided because of the nature of the app and its effect on blinding.   The funnel plots were symmetrical, suggesting that there was no publication bias.  Although some of the studies used commercial apps, the study team developed most of them. | **Low**  One critical flaw (no pre-registered protocol)  More than one non-critical weakness (missing information on inclusion criteria for comparator groups, no information on funding sources) |
| Seo 2015 [73]  Meta-analysis | eHealth  Interventions using the Internet as a major tool and targeting lifestyle (promote healthy diet, physical activity, or both). | Searches:  1980-2014  Publications:  2001-2014 | 31 studies  RCTs | General population  N=8,442 | Waist circumference | *Main effects, intervention versus minimal intervention:*   - Waist circumference reduction (mean loss 2.38 cm; 95% CI 1.51 to 3.25; p<.001; I^2^=97.2%; k=24)   *Main effects, intervention versus paper-, phone-, or person-based interventions:*   - No difference in effect with respect to the waist circumference change (mean change −0.61 cm; 95% CI −2.05 to 0.83; P=.42).   *Other relevant findings:*   - Overall, Internet-based interventions significantly reduced waist circumference (mean change −2.99 cm, 95% CI −3.68 to −2.30), as did minimal interventions (mean change −0.81 cm; 95% CI −1.41 to −0.20) and other interventions (mean −2.82 cm; 95% CI −3.89 to −1.74). - Large and significant between-study heterogeneity was observed (I^2^=93.3%, P<.001). | *Meta-regression*  *Significant effects on waist circumference were observed for Internet interventions that provided:*   - 3.1 Social support (unspecified) (mean difference −1.16 cm, P=.03). - No significant effects were observed for monitoring, goal setting, motivational interviewing, or the number of components. | - Tailoring (n=21), - 3.4 Self-monitoring of outcomes of behaviour) (n=21) - 1.1 Goal setting (behaviour) or 1.3 Goal setting (outcome) (n=15) - Motivational interviewing (n=4) - 3.1 Social support (unspecified) (n=11) - 10.8 Incentives (outcome) (n=1) | *Cochrane Collaboration’s Risk of Bias:*  **results only partially reported*   - 17/31 studies provided details on random sequence generation - 18/31 studies provided details on allocation concealment - only 2/31 studies reported blinding participants, and 9/31 studies reported blinding assessors - The bias assessment indicated no evidence of selective reporting of outcomes. - no significant publication bias was detected (*P*=.31 for Begg’s test) for Internet-based interventions as evaluated by the waist circumference change in each study arm. | **Critically Low**  More than one critical flaw (no pre-registered protocol, no consideration of bias when interpreting results)  More than one non-critical weakness (missing information on inclusion criteria for comparator, no justification for selection of study design, data extraction not done in duplicate, excluded studies not listed, no information on funding sources, impact of risk of bias not assessed) |
| Sherrington 2016 [74]  Systematic review and meta-analysis | eHealth  At least in part via the internet. Incorporating any form of individualized feedback to the participants either human-delivered or computer-generated personalized feedback (using algorithms that sent pre-programmed responses based on participant input or choices) delivered via web-based messages or email. | Searches:  Inception -2012  Publications:  2001-2012 | 12 studies reported in 14 articles  RCTs | General population and clinical population: Adults with BMI > 25 kg/m^2^  N=3,547 | Weight loss | *This study focused on personalized feedback* | *Main effects, interventions with personalized feedback versus control with no feedback:*   - Weight loss MD -2.13 kg (95% CI -2.97 to -1.29; p<0.00001; I^2^=99%; k=12)   *Other relevant findings:*   - All outcomes were found to be statistically and clinically (≥5% body weight loss) significant for study end of intervention results and at 3 and 6 months, but not at 12 months. - Only BMI and waist circumference outcomes illustrated statistically significantly greater losses for the internet-delivered interventions with personalized feedback compared with the control groups receiving no personalized feedback ≥ 12 months. - The most effective studies in terms of weight loss (kg) ranged from 7 to 14 BCTs and were not consistent in relation to included BCTs. | - 2.2 Feedback on behaviour (k=13) - 5.1 Information about health consequences (k=13) - 2.3 Self-monitoring of behavior (k=13) - 1.1 Goal setting (behavior) (k=9) - 3.1 Social support (unspecified) (k=8) - Self-monitoring of outcome of behaviour (k=8) - 4.1 Instruction on how to perform a behavior (k=7) - 1.3 Goal setting (outcome) (k=6) - 1.2 Problem solving (k=5). | *Cochrane Collaboration’s Risk of Bias:*   - Only 2/12 studies were of low risk of bias for all criteria. - Selective reporting was the only criterion to receive high risk of bias scores for 4 studies.   3 studies provided monetary incentives for the completion of assessments that may have acted as a co-intervention in respect of retention rates. | **Critical Low**  More than one critical flaw (no consideration of bias when interpreting results, no assessment of publication bias)  More than one non-critical weakness (no justification for selection of study design, data extraction not done in duplicate, excluded studies not listed, insufficient details on included studies, no information on funding sources, impact of risk of bias not assessed) |
| Lyzwinski 2014 [75]  Systematic review and meta-analysis | mHealth  New generation use mobile devices that are commercially available, including: Mobile phones, smartphones, iPads, iPods, MP3 players, and Personal Digital assistants (PDA’s). PDA’s were included if they were of newer generation with updated feedback thermometers installed. | Searches:  Up to 2013  Publications:  2007-2013 | 12 studies reported in 17 articles (5 secondary analyses)  RCTs | General population with overweight or obesity  N=1,300 | Weight loss, diet and physical activity | *Main effects, intervention versus control:*  Weight loss (kg) -medium significant effect size SMD 0.43 (95% CI 0.252 to 0.609; p<0.001; I^2^=44.6%; k=11) | *Quantitative analysis: Not reported* | - 2.3 Self-monitoring of behaviour (k=12) - 1.1 Goal setting (behavior) (k=12) - 2.2 Feedback on behaviour or 2.7 Feedback on outcomes of behaviour (k=10) - 5.1 Information about health consequences (k=8) - Encouragement (k=6) - 8.1 Behavioral practice/rehearsal (k=7) - 3.1 Social support (unspecified) (k=unclear). | *Cochrane handbook for trial appraisal risk of bias:*   - 6/12 studies were graded as having a low risk of bias by meeting at least 3 of the 5 domains.   The funnel plot indicated some possibility of publication bias. | **Critical Low**  More than one critical flaw (no pre-registered protocol, appropriateness of meta-analytic method unclear)  More than one non-critical weakness (no justification for selection of study design, study selection and data extraction not done in duplicate, excluded studies not listed, no information on funding sources, no assessment of impact of risk of bias, no discussion of heterogeneity) |
| Levine 2015 [67]  Systematic review | eHealth and mHealth  Web-based applications, clinician-guiding software, home PCs, mobile applications, and short message services (SMS, “texting”). | Searches:  2000-2014  Publications: 2001-2013 | 16 studies  RCTs | General population via a primary care setting  N=6,786* | Weight loss | *Main effects: Not reported*  *Other relevant findings:*   - Compared to the control group, most (12/16, 75%) technology-assisted interventions achieved weight loss at the end of the study period. - Weight loss in active treatment arms ranged from 0.08 kg to 5.4 kg (0.8% – 5.8% of initial body weight). - The percentage of patients losing at least 5% of baseline weight ranged from 5% to 45%. - The degree of weight loss in this review compares favorably to other primary care-based weight loss interventions without technology. | *Quantitative analysis: Not reported*  *Other relevant findings:*   - Interventions employing clinician-guiding software and feedback from personnel may be more likely to promote weight loss, as 86% and 85% of studies using these tools showed significant weight loss, respectively. - In contrast, interventions without personnel (fully automated) were less likely to do so, with only 33 % demonstrating weight loss, suggesting that technology cannot fully replace human interactions with the healthcare team.   2.3 Self-monitoring of behaviour via technology occurred in 11/13 (85 %) studies reporting weight loss (1.3–5.4 kg), compared to paper and pencil methods in 2/13 (15 %) reporting weight loss (1.1–3.96 kg). | - 2.3 Self-monitoring behaviour (k=13; 81 %) - Automated 2.2 Feedback on behaviour (75%) - Lifestyle coaching (68%) - In-person feedback (62%) - 7.1 Prompts/cues [reminders] (43%), - MD feedback (31%) - 3.1 Social support (unspecified) [person-to-person support] (31%) | *Delphi (Verhagen et al. 1998) and Cochrane Effective Practice and Organization of Care (EPOC) criteria:*   - Trials scored between 4 and 8 of nine points for both the Delphi and EPOC bias criteria. - Of studies meeting 7 or more EPOC criteria, weight loss ranged from 1.5 kg to 5.4 kg, whereas those meeting less than 7 EPOC criteria reported 0.08 to 3.36 kg weight loss. - Blinding of the provider, patient, and outcome assessor were   the most common sources of bias. | **Moderate**  No critical flaws  More than one non-critical weakness (missing information on inclusion criteria for population and comparator, no justification for selection of study design, excluded studies not listed, no information on funding sources, no discussion of heterogeneity) |
| Knowlden 2022 [68]  Systematic review | eHealth  Websites, phone or video delivery, text messaging, group interaction (e.g., discussion boards) | Searches: 2000-2021  Publications:  *Not reported* | 11 studies  RCTs and quasi-experimental trials | General population: Normal weight, overweight or obese adult males  N=1,748 | Obesity prevention | *Main effects: Not reported*  *Other relevant findings:*   - 9/11 studies produced significant results on the primary outcome however of these, only five produced group-by-time intervention effects - 4/9 trials reporting significant weight loss reported clinically significant weight loss ≥5% weight loss from baseline | *Quantitative analysis: Not reported* | - 2.3 Self-monitoring of behaviour (k=10) - 2.2 Feedback on behaviour or 27 Feedback on outcomes of behaviour (k=8) - 5.1 Information about health consequences [health counseling] (k=8) - 1.1 Goal setting (behaviour or 1.3 Goal setting (outcome) (k=9) | *Modified version of the Jadad Scale:*   - *Quality assessment scores ranged from 5 (k=1), 7 (k=5), and 9 (k=5), with a mean score of 7.72.* | **Moderate**  No critical flaws  More than one critical weakness (no justification for selection of study design, excluded studies not listed, no discussion of heterogeneity) |
| Wright 2021 [69]  Systematic review | eHealth  Online programs or internet modules, telephone, text messages, videoconferencing, mobile application, audiovisual media, wireless fidelity scales, emails, access to a private Facebook group. | Searches:  Inception to January 2022  Publications: 2011-2021 | 14 studies reported in 17 papers  RCTs, quasi-RCTs or controlled before and after studies | Clinical population: Adults that have undergone or have a confirmed, scheduled bariatric procedure  N=1,633 | Postoperative weight loss, weight loss maintenance, eating psychopathology, QoL, depression screening, and self-efficacy | *Main effects: Not reported*  *Other relevant findings:*   - Weight loss: Overall, eHealth was found to be as effective as or more effective compared with control groups, in all except 1 study where the control significantly improved weight loss percentage - Eating psychopathology: 6/8 papers reported significant improvements - QoL: 3/7 papers reported significant improvements versus control. - Depression: 1/5 papers reported an improvements - Self-efficacy: 4/4 papers reported significant differences versus control. | *Quantitative analysis: Not reported* | - 2.3 Self-monitoring of behavior (k=6) - Shaping knowledge (k= 6) - 3.1 Social support (unspecified) (k=5) - 1.3 Goal setting (outcome) (k=5) - 1.2 Problem-solving (k=5) - 2.4 Self-monitoring of outcomes of behavior (k=5) | *Cochrane Collaboration’s Risk of Bias:*   - Overall bias was low for 5 papers, 7 papers had some concerns, and high risk for 5 papers. - For the randomization process, 41% of studies had some concerns or high risk; - For missing outcome data 47% had some concerns or high risk | **Low**  One critical flaw (no consideration of bias when interpreting results)  More than one non-critical weakness (missing information on inclusion criteria for outcomes, no justification for selection of study design, excluded studies not listed, no discussion on heterogeneity) |
| Willmott 2019 [70]  Systematic review | eHealth  Web-based (e-learning, website, email, e-counselor, e-newsletter, and Wi-Fi enabled scale, social network sites), mobile-based, and multicomponent interventions. | Searches:  Up to 2018  Publications:  2006-2018 | 24 studies  RCT, NRCTs, and cohort studies (pretest-posttest and posttest only) | General population: healthy participants free of acute illness or chronic disease  N=31,747* | Weight management | *Main effects: not reported*  *Other relevant findings:*   - 8/12 studies reported significant positive weight-related changes (e.g., maintenance of a healthy weight or reversal of small gains), and 4 reported mixed outcomes. - 712/24 studies did not report any statistically significant changes in the weight-related measure(s). | *Quantitative analysis: Not reported* | - 2.3 Self monitoring of behaviour (k=19) - 1.1 Goal setting (behaviour) (k=10) - Tailored or personalized Feedback (k=10) - 3.1 Social support (unspecified) (k=9) - Contact with an interventionist (k=6) - 7.1 Prompts/cues (k=6) | *Effective Public Health Practice Project’s (EPHPP) quality assessment tool:*   - 19/24 studies were rated as weak, 5/24 as moderate, and none as strong. | **Low**  One critical flaw (no pre-registered protocol)  More than one non-critical weakness (missing information on inclusion criteria for population, no justification for selection of study design, excluded studies not listed, no information on funding sources, no discussion on heterogeneity) |
| Rhodes 2020 [76]  Systematic review and meta-analysis | mHealth  Text messaging, apps, websites. | Searches:  up to -2019  Publications:  2012-2018 | 11 studies  RCT, pilot studies | General population: pregnant women  N=3,280* | Gestational weight gain | *Main effects, intervention versus control:*   - ITT data showed a non-significant effect of the interventions, with a mean difference in total GWG of−0.28 kg (95% CI −1.43 to 0.87; I^2^=0%; k=3). - The mean difference in total GWG for per protocol data was −0.65 kg (95% CI −1.98 to 0.67; *I^2^*=53%; k=4).   *Other relevant findings:*   - 3/11 studies reported significant positive effects of their interventions on gestational weight gain (GWG) and physical activity in comparison with control groups. - Most studies were pilot RCTs and insufficiently powered to detect an effect, however these findings indicate that exclusively digital interventions to manage GWG may be less effective than those using interpersonal delivery. | *Quantitative analysis: Not reported*  *Other relevant findings:*  7 BCTs were common to the 3 effective interventions:   - 1.1 Goal setting (behavior) - 1.2 Problem solving - 1.5 Review of behavior goals - 2.2 Feedback on behavior - 3.1 Social support (unspecified) - 5.1 Information about health consequences - 5.6 Information about emotional consequences - 1.5 Review of the behavior goal was the only BCT used exclusively in the 3 effective interventions. - The 3 information-only interventions that included no active or interactive BCTs, such as goal setting, self-monitoring, problem solving, or feedback, were ineffective. - The 3 effective interventions used, on average, twice the number of BCTs compared with other interventions (mean 14, SD 2.9 vs mean 6.8, SD 4.1). | - 5.1 Information about health consequences (k=11) - 1.1 Goal setting (behavior) (k=8) - 1.2 Problem solving (k=7) - 2.3 Self-monitoring (behavior) or 2.4 Self-monitoring of outcome of behaviour (k=7) | *Cochrane Collaboration’s Risk of Bias:*   - Five studies were deemed to have an overall high risk of bias, three had a low risk of bias, and three were classified as having some concerns. | **Critical Low**  More than one critical flaw (no consideration of bias when interpreting results, no assessment of publication bias)  More than one non-critical weakness (no justification for selection of study design, excluded studies not listed, no information on funding sources, impact of risk of bias not assessed, no discussion of heterogeneity) |
| Besson 2020 [71]  Systematic Review | e- and mHealth  Personal computers, mobile phones or smartphones and personal digital assistants (PDA), including Interactions with the participants managed through websites, specific software for the PDA study, SMS, social networks or mobile applications (apps). | Searches: 2004-2018  Publications:  2004-2018 | 15 studies  RCTs and case control trial | General population: Overweight participants who were free from other acute illnesses or chronic disease  N=not reported | Weight loss | *Main effects: Not reported*  *Other relevant findings:*   - 9/15 studies showed a significant difference in weight loss between conditions, in favour of one (or more) intervention arm. - The remaining studies failed to find significance between conditions but were using a comparison with an active intervention, potentially indicating equivalent efficacy. | *Quantitative analysis: Not reported*  *Other relevant findings:*   - Self-monitoring was included in the intervention arm(s) that were found to be effective compared to information only comparisons or no treatment controls and also in studies finding no difference between interventions and information-only comparison. - Feedback was also present in the intervention arm(s) of effective interventions and in those found to be no different to controls. | - 5.1 Information about health consequences (n=15) - 2.3 Self-monitoring behavior or 2.4 Self-monitoring outcomes of behavior (n=15) - 2.2 Feedback on behavior or 2.7 Feedback on outcomes of behavior (n=10) - 3.1 Social support (unspecified) (n=9) - 1.1 Goal setting (behaviour) or 1.3 Goal setting (outcomes) (n=7) - 7.1 Prompts/cues n=7) - 1.4 Action planning (n=6) - 10.10 Reward (outcome) (n=2) - 1.2 Problem-solving (n=2) | *Not reported* | **Critical Low**  More than one critical flaw (no pre-registered protocol, no consideration of risk of bias when interpreting results)  More than one non-critical weakness (excluded studies not listed, no explanation for selection of study designs, no information on funding sources, no discussion of heterogeneity, no information of conflict of interest) |
| Physical activity and sedentary behaviour |  |  |  |  |  |  |  |  |  |  |
| Laranjo 2020 [85]  Systematic review, meta-analysis and meta regression | mHealth  Activity trackers, mobile apps, email, human involvement (face-to-face or phone calls) and text messaging | Searches: 2007-2020  Publications:  2014-2019 | 35 studies; 28 studies included in the meta-analysis  RCTs | General population  N=7,454 | Physical Activity | *Main effects, intervention versus true and active control:*   - PA (SMD 0.350, 95% CI 0.236 to 0.465, p<0.0001, I^2^ =69%; k=28), corresponding to an increase of 1850 steps per day (95% CI 1247 to 2457). | *Subgroup analyses:*  *Significant effects on PA were observed for interventions that provided:*   - Goals and planning (SDM 0.446, 95% CI 0.33 to 0.562, p<0.0001) - Graded tasks (SDM 0.512, 95% CI 0.337 to 0.687, p=0.031) - Text messaging (SDM 0.495, 95% CI 0.335 to 0.654, p=0.028) - Personalisation (SDM 0.541, 95%CI 0.365 to 0.718, p=0.006) - studies where the authors mentioned conflicts of interest (SDM 0.529, 95%CI 0.388 to 0.671, p=0.004) - studies mentioning behavior change theories (SDM 0.449, 95%CI 0.312 to 0.587, p=0.018). | - 1.1 Goal setting (behavior) (k=19) - 7.1 Prompts/cues (k=16) - 4.1 Instruction on how to perform the behavior (k=14) - 3.1 Social support (unspecified) (k=9) - Gamification or exergames (k=14) - Personalisation features (k=12) | *Cochrane Collaboration’s Risk of Bias:*   - Risk of bias was assessed as low for at least 4 out of 6 categories in 17 studies.   *GRADE:*   - The available evidence is of low-to-moderate quality - Adjusted results accounting for the presence of publication bias remained significant. | **Moderate**  No critical flaws  More than one critical weakness (no justification for selection of study design, data extraction not done in duplicate) |
| De Leeuwerk 2022 [86]  Systematic review and meta-analysis | mHealth  Activity trackers | Searches:  Up to March 2021  Publications: 2005-2020 | 21 studies  RCTs | Clinical population:  adults <=3months after hospitalization or inpatient rehabilitation. No restrictions on the medical reason of the inpatient period.  N=2,355 | Physical activity, physical functioning | *Main effects, intervention versus control:*   - PA: a significant small positive effect in favour of the intervention (SMD = 0.34; 95%CI 0.12 to 0.56; I^2^=73%; k=13) - Physical functioning (13/21 studies): no significant effect (SMD=0.09; 95%CI -0.02 to 0.20; I^2^=8%; k=13). - There was considerable heterogeneity between studies. | *Meta-regression was not possible*  *Subgroup analyses:*  Significantly enhanced effectiveness on PA was observed for interventions with the following features compared with those without:   - ≥7 BCTs (SMD=0.60, 95%CI 0.18;1.02, *p* =0.005) - Theory-based interventions with activity trackers (SMD = 0.66, 95%CI 0.14; 1.18, *p* =0.01) - Interventions with coaching by a health professional (SMD = 0.44, 95%CI 0.19; 0.69, *p* = 0.0004). | - 2.2 Feedback on behaviour (k=23) - 1.1 Goal setting (behaviour) (k=15), - 1.4 Action planning (k=12) - 2.3 Self-monitoring of behaviour (k=15) - 8.7 Graded tasks (k=12) - 12.5 Adding objects to the environment (k=15) | *PEDro:*   - The PEDro score of the included trials ranged from 3 to 8. - 13 studies were judged as low risk of bias - 8 studies were judged as high risk of bias. - Blinding of participants and therapists was not possible in any study due to the intervention setting.   Egger’s regression test indicated no significant asymmetry of the funnel plots therefore unlikely influence of publication bias. | **Moderate**  No critical flaws  More than one non-critical weakness (excluded studies not listed, no justification for selection of study design, no information on funding sources, publication bias reported but not discussed) |

| Vetrovsky 2022 [87]  Systematic review and meta-analysis | mHealth  Pedometers, wearable fitness trackers, and smartphone apps | Searches:  Up to 2022  Publications:  2007-2022 | 85 studies  RCTs | Clinical and general population  N= 12,057 | Physical activity | *Main effects, intervention versus control:*   - PA: post-intervention MD 926 steps/day (95% CI 651 to 1201). - PA: follow-up, MD 413 steps/day (95% CI 210 to 615). | *Sensitivity analyses*  showed little change to the pooled effect sizes when excluding studies with:   - Active control arms receiving an additional PA intervention component beyond activity monitor and a set goal (k=32; MD 1023, 95% CI 772 to 1275; I2 52%) - Studies with active control arms receiving a non-PA intervention (k=66; MD 915, 95% CI 613 to 1217; I^2^ 87%) - studies with intervention duration <4 weeks (k=74; MD 883, 95% CI 599 to 1166; I^2^ 88%) - studies with sample size <40 (k=63; MD 824, 95% CI 523 to 1125; I^2^ 89%). - When removing studies with active control arms not having a set goal, the MD between the intervention and active control arms substantially decreased (k=48; MD 710, 95% CI 459 to 961; I^2^ 78%).   *Moderator analysis:*   - Only intervention duration, the presence of a set goal, and the addition of human counselling (especially via phone/video calls) demonstrated significant moderating effects. | *Not reported* | *Cochrane Collaboration’s Risk of Bias:*   - Random sequence generation and reporting bias varied a lot between studies (54 low, 14 unclear and 17 high risk of bias). - Allocation concealment was generally poorly reported, with a large number of studies being assessed as having an ‘unclear’ risk of bias for this domain (34 low, 49 unclear and 2 high risk of bias). - The majority of studies were judged to be at high risk of performance bias (7 low, 4 unclear and 74 high risk of bias due to unavoidable lack of blinding.   Egger’s regression test indicated no significant asymmetry of the funnel plots therefore unlikely influence of publication bias. | **Moderate**  No critical flaws  More than one non-critical weakness (excluded studies not listed, no information on funding sources) |
| --- | --- | --- | --- | --- | --- | --- | --- | --- | --- | --- |
| Western 2021 [88]  Systematic review and meta-analysis | E and mHealth  Websites, activity trackers, text messaging or email feedback or prompts, and mobile applications. | Searches:  1990-2020  Publications:  2007-2019 | 19 studies  RCTs | Clinical and general population  N=5,419 | Physical activity | *Main effects, intervention versus control:*   - PA in low SES groups: SMD 0.06 (95% CI -0.08 to 0.20; I^2^=18%). - PA In high SES groups: SMD 0.34 (95% CI 0.22 to 0.45; I^2^=0%). | *Subgroup analyses:*   - of individual BCTs with more than one constituent indicator (goals and planning, feedback and monitoring, shaping knowledge, natural consequences, comparison of behaviour, reward and threat, and ante- cedents) did not indicate sub-group effects of PA.   *Meta-regression:*   - There was no association with the number or type of BCTs. | - 2.3 Self-monitoring of behaviour (81%) - 1.1 Goal setting behavior (76%) - 2.2 Feedback on behaviour (76%) - 1.2 Problem-solving (52%) - 1.4 Action planning (52%) - 5.1 Information about health consequences (48%) - 1.5 Review behaviour goal (43%)   3.1 Social support (unspecified) (43%) | *Cochrane Collaboration’s Risk of Bias (RoB2):*   - 4/19 studies were considered low risk of bias for all categories - 4 studies had one category judged to have some concerns - 6 studies had two categories judged to have some concerns. - 4 studies had at least one category judged as high risk of bias. - Given the behavioural nature of the trial, blinding to allocation was not possible in any of the studies. | **Moderate**  No critical flaws  More than one non-critical weakness (excluded studies not listed, no information on funding sources) |
| Davies 2012 [89]  Meta-analysis | eHealth  Internet and/or email. | Searches:  1990-2011  Publications:  2001-2011 | 34 studies  RCT or quasi-experimental studies | General population (50%) and clinical populations (50%) including; overweight (n = 5), Type 2 diabetes (n = 4), arthritis (n = 1), cardiac rehabilitation (n = 1), metabolic syndrome (n = 1), physical disabilities (n = 1), chronic disease (n = 1), multiple sclerosis (n = 1), a diagnosed mental illness (n = 1), and cardio obstructive pulmonary disorder (n = 1)  N=11,885 | Physical activity | *Main effects, intervention versus comparison:*   - PA (d= 0.14; p<0.001; k=34) - The overall mean effect for sustained PA at least 6 months post-intervention resulted in a small but significant effect size (d= 0.11; p<0.01; k=11). - Homogeneity tests from the fixed-effect analysis revealed significant heterogeneity across studies (Q= 73.75; p<0.001). | *Moderator analysis:*  Significant effects of PA change was observed for interventions with the following features compared with those without:   - 5.1 Information about health consequences [education (exchange of information intended to influence physical activity)] (d= 0.20) | - 2.2 Feedback on behaviour (k=28) - 5.2 Information about health consequences [education] (k=24) - 7.1 Prompts/cues [email reminders] (k=21) - 1.1 Goal setting (behaviour) (k=21) - 2.3 Self-monitoring of behaviour (k=19) - Facilitator (k=15) - Asynchronous communication (k=15) - Quiz (k=13) - Synchronous communication (k=6) | Modified tool from Haynes et al. 1979:   - None of the studies were rated as poor; 10 (29%) were rated fair, and 24 (71%) as good quality.   The results of the Egger test revealed that publication  bias was present (p<0.001) | **Low**  One critical flaw (no pre-registered protocol)  More than one non-critical weakness (excluded studies not listed, study selection not done in duplicate, no information on funding sources) |
| Direito 2017 [90]  Systematic review and meta-analysis | mHealth    mHealth technologies employed were PDA, mobile phones/SMS, biosensors, smartphones/apps, tablet computers and websites | Searches:  Inception-January 2015  Publications:  2007-2015 | 21 studies  RCTs | General population: (young people≤18 years and adults≥18 years) with no pre-existing medical conditions or contraindications  N=1,701 | Physical activity and sedentary behavior | *Main effects, intervention versus control:*   - Sedentary behavior: (SMD = −0.26, 95 % CI −0.53 to −0.00; I^2^=0%).   Summary effects across studies were small to moderate and non-significant for   - total PA (SMD 0.14; 95 % CI−0.12 to 0.41; I^2^=60%) - MVPA (SMD 0.37; 95 % CI−0.03 to 0.77; I^2^=78%) - Walking (SMD 0.14; 95 % CI−0.01 to 0.29; I^2^=0%) | *Quantitative analysis: Not reported* | - 1.1 Goal setting (behavior) (81%) - 2.3 Self-monitoring of behavior (74%) - 3.1 Social support (unspecified) (65%) - 2.2 Feedback on behavior (55%) - 4.1 Instruction on how to perform the behavior (55%) - 12.5 Adding objects to the environment (48%) - 5.1 Information about health consequences (45%) - 7.1 Prompts/cues (45%). | *Cochrane Collaboration’s Risk of Bias:*   - Incomplete reporting of methods hindered risk of bias judgement for several studies. - Allocation concealment approaches were mainly judged at unclear risk of bias. - Studies were judged at high risk of performance bias since it is impractical and very hard to blind participants to a PA behavior change intervention. | **Low**  One critical flaw (no consideration of bias when interpreting results)  More than one non-critical weakness (excluded studies not listed, no information on funding sources) |
| Stephenson 2017 [91]  Systematic review and meta-analysis | E and mHealth  Software/computer prompts, emails, websites to relay information and provide feedback, text messages, activity monitors with online companion app. | Searches:  Up to 2016  Publications:  2012-2016 | 17 studies  RCTs | General population  N=1,967 | Sedentary behavior | *Main effects, intervention versus comparison:*   - Sedentary behavior: mean reduction of−41.28 min/day (95% CI -60.99 to −21.58; I^2^= 77%; k=15). - Objectively measured sedentary behavior: mean reduction −35.07 min/day (95% CI -46.5 to −23.57; I^2^=21%; k=8) - Subjectively measured sedentary behavior: mean reduction of −52.66 min/day (95% CI −93.63 to −11.69; I^2^=88%; k=7). - For studies reporting short-term measures (≤3 months), the pooled analysis showed a mean reduction of −42.42 min/day (95% CI -63.2 to −21.63; I^2^=61%; k=10) in favor of the intervention group. - For studies reporting medium-term (>3 to 6 months) measures, the pooled effect showed a mean reduction of −37.23 min/day (95% CI -73.70 to −0.75; I^2^=85%; k=5). - 3 studies reported long-term measures of sedentary behavior (>6 months), with pooled analysis showing a mean reduction of −1.65 min/day (95%CI -14.77 to 11.47; I^2^=23%). | *Quantitative analysis: Not reported* | - 4.1 Instruction on how to perform a behavior (k=15) - 3.1 Social support (unspecified) (k=12) - 7.1 Prompts/cues (k=11) - 12.5 Adding objects to the environment (k=11).   The most frequently coded BCTs for computer, mobile and wearable components of the interventions were:   - 7.1 Prompts and cues (k=10), - 2.3 Self-monitoring of behavior (k=7) - 3.1 Social support (unspecified) (k=7) - 1.1 Goal setting (behavior) (k=5). | *Cochrane Collaboration’s Risk of Bias:*   - Overall, 13 studies were judged to have a high risk of bias based on: allocation concealment, blinding of outcome assessment, and incomplete outcome data. - 3 studies were deemed to be at an unclear risk of bias due to incomplete outcome data and allocation concealment. - 1 study was judged to be at a low risk of bias. | **Low**  One critical flaw (no assessment of publication bias)  More than one critical weakness (no justification for selection of study design, study selection and data extraction not done in duplicate, excluded studies not listed, no information on funding sources, impact of risk of bias not assessed) |
| Tong 2018 [92]  Systematic review and meta-analysis | mHealth  Studies used mobile apps in isolation or as part of a more complex intervention with other components (e.g., wearable activity trackers). | Searches:  Up to 2018  Publications:  2012-2017 | 19 studies  RCTs, quasi-experimental studies and non-experimental studies | General population and clinical population including chronic obstructive pulmonary disease (n=1), attention deficit hyperactivity disorder (n=1), prostate cancer (n=1), child-hood cancer survivors (n=1), and stroke survivors (n=1)  N= 953* | Physical activity | *Main effects, intervention versus comparison:*   - PA: no significant effect (SMD= 0.957, 95% CI −1.09 to 3.00; I^2^= 99.6%; k=4). | *Quantitative analysis: Not reported*  *Other relevant findings:*  In 3 studies exploring participant preferences, goal setting, reward for progress in performing PA and personalized feedback were the most preferred features. | - 2.3 Self-monitoring of behavior [facilitated via wearable activity trackers] (k=12) - 3.1 Social support (k=17) - 1.1 Goal setting (behaviour) (k=6) | *Cochrane Collaboration’s Risk of Bias:*   - 1/4 included RCTs, 2 studies were deemed as having the lowest risk of bias. - All studies had a low risk of bias for random sequence allocation, and a high risk of bias for blinding of participants and personnel. - Non-RCTs had a higher risk of bias; however detailed assessment was not possible due to the quality of reporting. | **Critical Low**    More than one critical flaw (no consideration of risk of bias when interpreting results, no assessment of publication bias)  More than one non-critical weakness (missing information on inclusion criteria for comparators, impact of risk of bias not assessed, no discussion on heterogeneity) |
| Fanning 2012 [93]  Meta-analysis | mHealth  Interventions delivered via SMS, PDA, smartphone app and/or native app. | Searches: 2000-2012  Publications:  2007-2012 | 11 studies  Experimental studies with a comparison group | General population  N=1,351 | Physical activity | *Main effects, intervention versus control:*   - PA: significant moderate overall weighted mean effect size of g = 0.54 (95% CI = 0.17 to 0.91, P = .005; I^2^= 88.61%; k=11).   *Other relevant findings:*   - Intervention components delivered via mobile phone yielded a significant moderate effect (g =.52,95% CI = 0.11 to .94, P = .01) on pedometer steps.   The effects were non-significant for both MVPA duration (g = 0.20, 95% CI = -0.19 to 0.60, P= .31) as well as for PDA delivered (g = .68, 95% CI = -0.88to 2.25, P = .39), with lacking significance in the latter likely due in large part to the small number of studies and considerable heterogeneity. | *Quantitative analysis: Not reported* | - 2.3 Self-monitoring of behaviour (k=5) - 2.2 Feedback on behavour (k=4) - 7.1 Prompts/cues (k=4) - 1.1 Goal setting (behaviour) (k=2) - 3.1 Social support (unspecified) (n=2) | *Guide to Community Preventative Services* data extraction form:   - Four studies were classified to be of “good” quality, and seven studies were classified to be of “fair” quality. | **Critical Low**  More than one critical flaw (no pre-registered protocol, no risk of bias assessment, appropriateness of meta-analytic method unclear, no consideration of bias when interpreting results, no assessment of publication bias)  More than one non-critical weakness (missing information on inclusion criteria for comparator groups and population, no explanation for selection of study designs, excluded studies not listed, no information on study designs, no information on funding sources, impact of risk of bias not assessed) |
| Davis 2020 [77]  Systematic review | mHealth  Tailored mHealth interventions consisting of counseling, self-monitoring, group texts, tailored SMS and emails, pedometer, personal coaching calls, websites, telecoaching, video games, chatroom, reminders. | Searches:  *Not reported*  Publications: 2013-2018 | 16 studies  RCT, cluster RCT, pilot RCTs, or quasi-experimental studies | General population (n=12) and clinical population (n=4) including diabetes (n=2), chronic obstructive pulmonary disease (n=1), and coronary heart disease (n=1).  N=2,309 | Physical activity | *Main effect: Not reported*  *Other relevant findings:*   - PA 10/16 studies (62.5%) reported significant improvements or greater PA levels for the intervention groups compared to the controls. - 6/16 studies found no between group differences. - Of the studies that found no between group differences, 2/6 reported decreases in PA outcomes from baseline to follow-up, 3/6 reported positive changes for both the intervention and control groups, and 1/6 reported no within group differences. | *Quantitative analysis: Not reported*  *Other relevant findings:*   - 3/5 studies reported significant improvements in PA for the tailored intervention arm compared to the non-tailored treatment arms. | - 2.2 Feedback on behavior (k=9) - 1.1 Goal setting (behaviour) (k=6) - Tailoring to personal preferences (e.g., name or time of day messages were sent; n=6) | *Cochrane Collaboration’s Risk of Bias:*   - For overall bias, 7 studies were judged to have some concerns, 7 were judged to be low risk, and 2 were judged to be high risk. | **Moderate**  No critical flaws  More than one non-critical weakness (excluded studies not listed, no justification for selection of study design, no information on funding sources, no discussion of heterogeneity) |
| Buckingham 2019 [78]  Systematic review | mHealth  Wearable activity monitors or trackers, smartphone apps, motivational or persuasive text messaging or e-mails, computer software or websites linked to the activity monitor, and dedicated social media groups. | Searches:  2007-2018  Publications:  2009-2018 | 25 studies  RCTs and quasi-experimental studies | General population: participants in the workplace  N=73,415* | Physical activity and sedentary behavior | *Main effects: Not reported*  *Data were not sufficient to calculate effectiveness outcomes.*  *Other relevant findings:*   - 14/25 studies (56%) reported a significant increase in one or more measures of PA, over time or relative to the control or comparison group. - 7/10 studies rated as ‘high’ or ‘moderate’ quality reported a significant impact of the intervention on PA. - 4/11 studies (36%) using a wearable activity monitor as a single mHealth tool reported a significant absolute or relative increase in PA, compared with 10/14 studies (71%) using smartphone apps or activity monitors combined with apps. - 9/14 studies (64%) using multi-component interventions reported a significant impact on PA compared with 5/11 studies (45%) that assessed standalone mHealth interventions. | *Quantitative analysis: Not reported* | - 2.3 Self-monitoring of the behavior or 2.4 Self-monitoring of outcome of behavior (k=22) - 2.2 Feedback on the behavior or 2.7 Feedback on outcome of behavior (k=21) - 1.1 Goal setting (behavior) or 1.3 Goal setting (outcome) (k=17) - 6.2 Social comparison (k=14) - 3.1 Social support (unspecified) (k=12) - 10.2 Material rewards and 10.1 Material Incentives (behavior) (k=11) - 5.1 Information about health consequences (k=11) - 7.1 Prompts/cues (k=9) - 1.4 Action planning (k=8) - 8.7 Graded tasks (k=4) - 4.1 Instruction on how to perform the behaviour (k=4) - 1.2 Problem solving (k=3) | *Effective Public Health Practice Project (EPHPP) tool:*   - Only one study was judged as ‘strong’ quality. - 9 studies were assigned a ‘moderate’ quality rating - 15 studies were given a ‘weak’ rating. | **Moderate**  No critical flaws  More than one non-critical weakness (excluded studies not listed, no explanation for selection of study designs, no information on funding sources) |
| Hohberg 2022 [79]  Systematic review | eHealth  Combination of one-on-one meetings via telephone, video call, in person, and web-based interventions including text message and chat. | Searches:  2000-2020  Publications:  2001-2019 | 25 studies  RCTs | Clinical and general population  N=5,923 | Physical activity | *Main effect: Not reported*  *Other relevant findings:*   - Of the 19 studies investigating blended care interventions reporting effect sizes, the range was between −0.31 and +0.92, i.e., from a negative (detrimental) to a large positive (beneficial) effect. - 10/19 studies revealed a small positive effect (d= 0.20 to 0.49) of blended care on PA compared to control. - 4/19 demonstrated medium to large positive effects (d=0.55 to 0.92). - 3/19 studies showed no relevant positive effect (*d*=0.00 to 0.19) - 2/19 studies reported a negative effect size. | *Quantitative analysis: Not reported* | - 2.2 Feedback on behaviour (k=17) - 2.3 Self-monitoring of behaviour (k=17) - 1.1 Goals setting behaviour (k=15) - 1.2 Problem solving (k=10) - 1.4 Action planning (k=8) - 5.1 Information about health consequences (k=7) - 3.1 Social support (unspecified) (k=6) - 1.5 Review behaviour goal (k=6) - 2.4 Self-monitoring of outcomes of behaviour (k=6) - 4.1 Instructions on how to perform the behaviour (k=5) | Risk of Bias tool:   - Overall, 56% (14/25) of the studies had a low risk of bias, 44% (11/25) had some concerns, and no study was rated with a high risk of bias. - Most of the concerns were about outcome measurement. | **Moderate**  No critical flaws  More than one non-critical weakness (no explanation for selection of study designs, no information on funding sources) |
| Muellmann 2018 [80]  Systematic review | eHealth  Websites, phone, and text messaging. | Searches: Inception to 2017  Publications:  1997-2017 | 20 studies reported in 25 articles  RCT or quasi-experimental | General population: older adults aged 55+ without severe pre-existing chronic medical conditions  N=6,671 | Physical activity | *Main effect: Not reported*  *Other relevant findings:*   - Participation in eHealth interventions to promote PA led to increased levels of PA when compared to no intervention control groups, at least in the short term. - However, the results were inconclusive regarding the question of whether eHealth interventions have a greater impact on PA behavior among older adults than non-eHealth interventions (e.g., print interventions). | *Quantitative analysis: Not reported* | - Tailored advice (k=20) - 1.1 Goal setting (behaviour) (k=15) - 2.3 Self-monitoring behaviour (k=15) - 2.2 Feedback on behaviour (k=15) - 4.1 Instruction on how to perform a behaviour (k=not reported) | *Cochrane Collaboration’s Risk of Bias:*   - Overall, risk of bias of the included studies was rated as moderate (n=11) to high (n=13). - Only one study had low risk of bias. | **Low**  One critical flaw (no consideration of bias when interpreting results)  More than one non-critical weakness (no justification for selection of study design, excluded studies not listed, no information on funding sources, no discussion of heterogeneity) |
| Xu 2022 [81]  Systematic review | mHealth  Gamification delivered through digital devices (eg, PCs, tablets, smartphones, and wearable devices). | Searches:  Inception to 2020  Publications:  2013-2020 | 50 studies  RCTs, NRCTs | General population and clinical population  N=9,977 | Physical activity and sedentary behavior | *Main effect: Not reported*  *Other relevant findings:*   - Step count: 8/16 (50%) studies reported that the gamification interventions exerted a positive impact compared to control - Total PA: 3/8 (38%) RCTs reported statistically significant difference between intervention and control - Light PA: 3/5 (60%) RCTs showed that compared with the control groups, the intervention groups spent more time in LPA - Moderate PA: 4 (100%) RCTs reported that the differences between the intervention and control groups were not significant, while 2 (100%) NRCTs reported significant effects. - Vigorous PA: 4 (100%) RCTs reported no difference between the intervention and control groups, while 2 NRCTs reported significant increases in intervention versus control - MVPA: 3/6 (50%) RCTs reported gamification interventions had positive effects on MVPA - Sitting time: 1/3 (33%) RCTs reported that the intervention group spent less time in sitting compared with the control group | *Quantitative analysis: Not reported* | - 1.1 Goal setting (behaviour) (k=30, 60%) - 10.10 Reward (outcome) (k=25, 50%) - 10.4 Social reward (k=22; 44%) - 2.2 Feedback on behaviour (k=21, 42%) - 6.2 Social comparison (k=12; 24%) - 3.1 Social support (unspecified) (k=2, 4%) - Challenges (k=6, 12%) - Competition (k=16; 32%) - Collaboration (k=16; 32%) | *Cochrane Effective Practice and Organization of Care Group risk-of-bias criteria*   - Generally, 58% (29/50) of the studies performed well with at least 6 of the 9 evaluation criteria reported as low risk. - As the RCTs and single-group pretest–posttest studies involved random sequence generation and allocation concealment, they were high risk. - Furthermore, because 38% (19/50) of the studies had no control group, the applicable criteria relating to between-group comparisons were not fulfilled. | **Low**  One critical flaw (no pre-registered protocol)  More than one non-critical weakness (missing information on inclusion criteria for population, no justification for selection of study design, excluded studies not listed, no information on funding sources) |
| Luo 2021 [82]  Systematic review | eHealth and mHealth  Automated conversational agents | Searches: Inception to 2020  Publications:  *Not reported* | 20 studies  RCT, quasi-experimental or qualitative | General population  N=1,780 | Physical activity | *Main effect: Not reported*  *Other relevant findings:*   - 6/10 RCTs found that participants in the conversational agent group outperformed participants in the control group on various PA measures. - 1/10 RCTs did not find significant difference between intervention an control - In 2/3 RCTs interacting with a conversational agent significantly increased step counts and self-reported activity across all conditions; however, including financial incentives and rewards further boosted activity levels. | *Quantitative analysis: Not reported* | - 5.1 Information about health consequences (k=15) - Motivational messages - 1.2 Problem-solving (k=14) - 1.1 Goal setting (behaviour) (k=13) - 2.3 Self-monitoring of behaviour (k=14) - 2.2 Feedback on behaviour (k=14) - Exercise tips (k=11) - 1.4 Action planning (k=5) - 7.1 Prompts/cues (k=4) - 10.1 Material incentive (behaviour)(k=2) | *Mixed Methods Appraisal Tool*   - Methodological quality varied across studies, and few studies adequately addressed issues of user engagement, safety and ethics. | Low  One critical flaw (no consideration of bias when interpreting results)  More than one non-critical weakness (no information on funding sources, no discussion of heterogeneity) |
| Hardeman 2019 [83]  Systematic review | mHealth  Just-in-time adaptive interventions aimed at increasing physical activity level and or reduce sedentary behavior. | Searches:  Up to 2018  Publications:  2011-2018 | 14 studies reported in 19 papers  Studies of any design, including qualitative and quantitative studies reporting data or findings from development work, feasibility or pilot studies and definitive evaluation studies | General population  N=616 | Physical activity | *Main effect: Not reported*  *Other relevant findings:*  There was mixed evidence for intervention effects on physical activity and sedentary behavior. However, no study was designed to be powered to detect effects on behavior and only six studies were randomized.  Randomized studies:   - 2 studies found the percentage time spent in sedentary behavior (primary outcome) decreased significantly in all three intervention conditions, compared to baseline (p<.005). - 1 study found that the number of episodes of prolonged inactivity (> 2 h) per day was lower (p< .02) when participants received inactivity reminders, compared to control when they did not receive reminders. - 3 studies found no statistically significant effects on physical activity. | *Quantitative analysis: Not reported*  *Other relevant findings:*  BCTS in studies with positive effects on behavior included   - Goal setting (behavior) - 1.4 Action planning - 1.6 Discrepancy between current behavior and goal - 2.2 Feedback on behavior - 7.1 Prompts/cues - 10.4 Social reward - 4.1 Instruction on how to perform the behavior | - 1.1 Goal setting (behavior) (k=14) - 7.1 Prompts/cues (k=14) - 1.4 Action planning (k=9) - 2.2 Feedback on behavior (k=11) | *Risk of bias not reported*  *mERA checklist:*   - 9/12 studies did not report any details on integration into existing health information systems, cost-assessment, user information and training, solutions for delivery at scale, adaptation of the intervention, data security/confidentiality protocols and alignment with national and regulatory guidelines. | **Critical Low**  More than one critical flaw (no risk of bias assessment, no consideration of bias when interpreting results)  More than one non-critical weakness (no explanation for selection of study designs, excluded studies not listed, no information on funding sources, no discussion of heterogeneity) |
| Triantafyllidis 2018 [84]  Systematic review | eHealth and mHealth  Web-based or mobile phone, sensing devices with accelerometer or pedometer. | Searches:  2012-2017  Publications:  *Not reported* | 24 studies  RCT, feasibility studies | General population and clinical population  N=8,221* | Physical activity | *Main effect: Not reported*  *Other relevant findings:*   - All RCTs targeting PA demonstrated significant increases in PA (n=7). These studies included inactive adults, young or middle-aged adults with a BMI between 25 and 35 kg/m^2^, pre- diabetics, and patients with coronary artery disease. - Significant weight loss was reported in 3/ 4 studies assessing weight (targeted at patients with metabolic syndrome, patients with lower extremity disabilities, and overweight adults) - Improved glycosylated hemoglobin was reported in 2/3 studies assessing glycose concentration (targeted at patients with diabetes). - Effectiveness was also reported in change for health risk behavior in inactive individuals, and process out- comes such as diagnosis of obesity and healthcare quality data for overweight children, both through the use of an electronic health record. | *Quantitative analysis: Not reported* | - 1.1 Goal setting (behaviour) (k=16, 67%) - 2.2 Feedback on behaviour (k=15, 63%) | *Effective Public Health Practice Project (EPHPP) tool:*   - There were 19 RCTs and 5 feasibility studies. - Based on the six EPHPP criteria for selection bias, design, confounders, blinding, data collection, and drop-outs, 2 RCTs were poorly rated and 17 were of moderate quality. - The quality of the feasibility studies was weak, mainly because of their non-randomized design and lack of blinding. | **Critical Low**  More than one critical flaw (no pre-registered protocol, no consideration of risk of bias when interpreting results)  More than one non-critical weakness (no justification for selection of study design, data extraction not done in duplicate, excluded studies not listed, no information on funding sources, no discussion on heterogeneity) |
| Nutrition and Diet |  |  |  |  |  |  |  |  |  |  |
| Villinger 2019 [96]  Systematic review and meta-analysis | mHealth  Mobile interventions using a fully automated mobile application and that assessed any kind of nutrition behavior.  App could also be used in combination with other intervention strategies such as groups sessions, weekly meetings and online tools, coaching calls, text messages, and emails, or face‐to‐face contact. | Searches:  2006-2017  Publications:  2011-2017 | 41 studies  RCT and pre-post studies | General population and clinical population:  overweight or obese patients (k=16)  diabetes or prediabetes patients (k=8)  non-clinical general population samples: k=15  N=6,348 | Nutritional outcomes | *Main effects, intervention versus control:*   - There was an overall significant small‐to‐medium positive effect for app‐based mobile interventions, with overall Hedges' g = 0.33 (CI 0.21 to 0.44, P < .001; I^2^=86.79%; k=41). - Behavioral outcomes: small significant effect size Hedges' g= 0.19 (CI 0.06 to 0.32, P= .004; I^2^ = 62.96%; k=21) - When separating behavioral outcomes only the effect for fruit and vegetable intake reached statistical significance with Hedges' g = 0.32 (CI 0.15 to 0.50; P < .001; Q(7) = 11.83; P = .106; I^2^ = 24.19%). - Nutrition‐related health outcomes: small to medium effect size Hedges' g = 0.23 (CI 0.11 to 0.36, P < .001; I^2^ = 84.15%) - When separating nutrition‐related health outcomes, the strongest effect was found for obesity indices with Hedges' g = 0.30 (CI 0.15 to 0.45; P < .001; I^2^ = 87.88%; k=32; outcome n = 76). - Body weight: g = 0.27 (CI 0.13 to 0.41; P< .001; I^2^= 81.93%; k=31; outcome n=39) - BMI: g = 0.37 (CI 0.18 to 0.55; P< .001; I^2^ = 81.55%; k=17; outcome n=21). - BP: g = 0.21 (CI 0.01 to 0.42; P= .043; I^2^= 73.81%; k=7; outcome n=19) - Blood lipids: g = 0.15 (CI 0.03 to 0.28; P= .018; I^2^ = 0.00%; k=5, outcome n=22). - Cholesterol: g = 0.37 (CI 0.04 to 0.71; P = .031; I^2^= 72.81%; k=5; outcome n=7). - Blood sugar was positive but not statistically significant, Hedges' g = 0.18 (P = .429). | *Meta regression:*   - No significant effect for the implemented BCT clusters and single BCTs (technique present vs. not present in the intervention) - The number of single BCTs implemented varied from 2 to 11 between studies, but there was no difference in effectiveness with increasing number of BCTs. - 19 BCTs were implemented in two or more intervention studies, however there was no evidence that any of the BCTs predicted the pooled effect size. - Hence, there does not appear to be a single effective approach to changing nutrition behaviors and their main nutrition‐related health outcomes. | - 1.1 Goal setting (behavior) (k=28) - 1.3 Goal setting (outcome) (k=14) - 1.5 Review behavior goal(s) (k=20) - 2.2 Feedback on behavior (k=37) - 2.3 Self‐monitoring of behavior (k=41) - 2.4 Self‐monitoring of outcome(s) of behavior (k=17) - 2.7 Feedback on outcome(s) of behavior (k=11) - 3.1 Social support (unspecified) (k=12) - 3.2 Social support (practical) (k=10), - 3.3 Social support (emotional) (k=12). | *CONSORT 2010 checklist:*   - Overall, study quality ranged from high (n=29) to fair (n=12). - 89% of the 27 RCTs were classified as “high” quality and 43% of the non‐RCTs were classified as being of “high” quality. - Areas of high risk were around reporting of sample size calculations and randomization. - Most of the studies did not include blinding procedures in their study design. Therefore, the results of the evaluation of risk of bias according to the six International Cochrane Collaboration criteria indicate a high or unclear risk of bias for the two blinding procedures criteria and the allocation concealment criterion. - There was no evidence of publication bias. | **Low**  One critical flaw (no pre-registered protocol)  More than one non-critical weakness (missing information on inclusion criteria for comparator, no justification for selection of study design) |
| Rodriguez Rocha 2019 [97]  Meta-analysis | eHealth    Internet-based, computer-based, and short-message service (SMS) interventions. | Searches:  1999-2018  Publications:  1999-2018 | 19 studies  RCT, cluster RCT, NRCTs | General population  N=6,894 | Fruit and vegetable intake | *Main effects, intervention versus control:*   - Fruit and vegetable intake: overall effect size 0.26 (SE=0.05; 95% CI 0.17 to 0.35; p< .001; I^2^ = 62.77). - Computer-based interventions as eHealth tool had the largest positive ES (0.44, p < .001; k=3), closely followed by SMS interventions (ES = 0.41, SE = 0.10, 95% CI 0.21 to 0.61, p < .01; k=3). - Internet-based interventions (k=9) showed a small positive ES of 0.19 (SE = 0.05, 95% CI 0.09 to 0.29, p < .001). - CD-ROM, mobile apps, and video game interventions (k=4) did not show a statistically significant positive ES. - These differences between eHealth tools were statistically significant. | *Moderator analysis:*   - Interventions using 7 to 8 BCTs (k=4) showed a statistically significant larger positive ES of 0.42 (SE= 0.10, 95% CI 0.21 to 0.62, p< .001) compared with interventions using 4 to 6 BCTs (k=7) and 1 to 3 BCTs (k=7). - There was no significant effect for 4.1 Instruction on how to perform behavior, 2.2 Feed- back on behaviour, 1.2 Problem solving, 1.1 Goal setting (behaviour), or 5.1 Information about health consequences.   *Subgroup analysis:*   - Tailored interventions (k=15) presented an overall statistically significant positive ES of 0.27 (SE = 0.05, 95% CI 0.16 to 0.37, p < .001), while nontailored interventions (k=4) showed a small positive ES of 0.22 (SE = 0.11, 95% CI 0.00 to 0.44, p = .05), though this difference was not significant between groups. | - 4.1 Instruction on how to perform behavior (k=13) - 2.2 Feedback on behaviour (k=9) - 1.1 Goal setting (behavior) (k=8) - 1.2 Problem solving (n=unclear) - 5.1 Information about health consequences (n=unclear). | *Cochrane Collaboration’s Risk of Bias and Agency for Healthcare Research and Quality Standards:*   - Most studies were of fair quality (*n* = 12), five of good quality, and only two studies were of poor quality of evidence. | **Critically low**  More than one critical flaw (no pre-registered protocol, no consideration of bias when interpreting results)  More than one non-critical weakness (study selection not done in duplicate, excluded studies not listed, no information on funding sources) |
| Harris 2011 [98]  Systematic review and meta-analysis | eHealth  Interactive computer software programs that tailored output according to user input. Users may interact with the programs as members of a small group, as well as individually. | Searches:  1990-2009  Publications:  1990-2010 | 43 studies  RCTs | General population and clinical population aged 13 years or older  N= 21,811* | Dietary behavior | *Main effects, intervention versus control:*  *Significant effects were observed for:*   - Servings of fruit and vegetables per day: weighted MD of +0.24 (95% CI 0.04 to 0.44; I^2^=83%; k=12) - Total energy consumed from fat per day: MD –1.4% (95% CI –2.5 to –0.3%; I^2^=77%; k=10)   No significant effect for:   - Total fat consumed per day: MD –0.78g (95% CI –2.5 to 0.95g; I^2^=28%; k=12) - Saturated fat intake per day: MD –0.24g (95% CI –1.44 to 0.96g; I^2^=78%; k=5) - Dietary fibre per day: MD +1.45g (95% CI –0.02 to 2.92g; I^2^=60%; k=2); - Daily energy intake: +4kcal (95% CI –85 to 93kcal; I^2^=13%; k=5) - BMI: MD –0.1kg/m^2^ (95% CI –0.7 to 0.4 kg/m^2^; I^2^=0%; k=9) - Weight: MD 0.6kg (95% CI –3.48 to 4.63; I^2^=86%; k=4) | *Quantitative analysis: Not reported* | - Tailoring of information or feedback (k=43; NB inclusion criteria) - 1.1 Goal setting (behavior) (k=14) - 2.2 Feedback on behaviour (k=14) - 5.1 Information about health consequences (k=13) - 1.2 Problem solving (k=13) - 2.3 Self-monitoring of behavior (k=14) - 4.1 Instruction on how to perform the behavior (k=12) - Review of behavioral goals (k=11),   3.1 Social support (unspecified) (k=10). | *Cochrane Collaboration’s Risk of Bias:*   - Four studies had low risk of bias across domains. The remaining studies had unclear or high risk of bias for one or more domains. - Highest risk of bias was observed for blinding.   *Effective Public Health Practice Project (EPHPP) quality assessment:*   - 13 studies were of moderate quality and 30 were of weak quality overall.   There was a strong suggestion of publication bias (effects on servings of fruit and vegetables per day; Egger’s test, *p* = 0.008). | **High**  One non-critical weakness (missing information on inclusion criteria for comparator groups) |
| Chen 2020 [94]  Systematic review | e- and mHealth  Personal digital assistant, online education, video games, smartphone applications | Searches:  up to 2018  Publications: 2008-2018 | 15 studies  RCTs and non-randomized experimental studies | General population  N=not reported | Eating behavior | *Main effect: Not reported*  *Other relevant findings:*   - PDA: Effect sizes for an increase in fruit and vegetable intake and a decrease in fat and energy were low to moderate (0.1–0.3) in most studies, except one small study (n=27 adults) where effect sizes were higher (0.7–0.9). - Online Education: The overall effect sizes in these web-based interventions were relatively low, but the high effect size 1.08 for increase in nutritional knowledge supported the importance of the internet for disseminating knowledge. - Video games: Effect sizes in these studies for nutritional knowledge and dietary change were high (0.6-0.8). - Smartphone Apps: Although effect sizes for this intervention ranged from low to medium, sodium intake was significantly reduced in two studies. | *Quantitative analysis: Not reported*  *Other relevant findings:*   - Several studies demonstrated improvements in interventions that used a PDA coupled with feedback. - Many trials reported that delivery of personalized feedback messages in real-time was critical for supporting self-regulation during weight loss programs. - The efficiency of all the interventions increased when coupled with tailored feedback and counselling. - This review found that any type of intervention should be combined with appropriate feedback and counselling in order to sustain the desired effects. | *Not reported* | *Cochrane Risk of Bias tool Version 2 (RoB2):*   - All studies were classified as low risk in the “selection of the reported result” and “measurement of the outcome” domains. - Some studies were identified as having high risk of bias in the “missing outcome data” and “deviations from intended interventions” domains. - Bias in other domains was largely unclear. | **Moderate**    No critical flaws  More than one non-critical weakness (excluded studies not listed, no information on comparator group, no explanation for selection of study designs, study selection not done in duplicate, no information on funding sources, no discussion of heterogeneity) |
| Murimi 2019 [95]  Systematic review | eHealth and mHealth  Nutrition / prevention  Websites, smartphone applications, text messages, and  online course. | Searches:  2009-2018  Publications:  2010-2018 | 27 studies  RCTs; pre-test and post-test design; and  quasi-experimental studies | General population.  N=35,548* | Nutrition-related including anthropometric measures BMI, waist circumference); bio-chemical measurements (eg, blood vitamin D concentration); dietary intakes; nutritional knowledge; preferences; attitudes; behaviors; self-efficacy; stage of change; dietary diversity score; or physical activity. | *Main effect: Not reported*  *Other relevant findings:*   - 10/27 studies met their primary objectives as evidenced by their reported results, while 16 studies partially met their stated objectives, and only 1 study did not meet any of its stated objectives. | *Quantitative analysis: Not reported*  *Other relevant findings:*  6 factors were associated with successful interventions:   - use of tailored messages and/ or individualized feedback - participant engagement, as measured by the level of interaction between investigators and participants - intervention duration 3 months or more - identification of specific targeted behaviors vs general health - alignment of intervention activities with stated objectives - use of theory-based interventions | *Not reported* | *GRADE:*   - 5 studies were assessed as having low risk of bias and 22 had moderate risk of bias. | **Critical Low**  More than one critical flaw (no pre-registered protocol, insufficient assessment of risk of bias)  More than one non-critical weakness (no justification for selection of study design, excluded studies not listed, no information on funding sources, no discussion of heterogeneity) |
| Medication adherence |  |  |  |  |  |  |  |  |  |  |
| Armitage 2020 [99]  Systematic review and meta-analysis | mHealth  Medication reminder app, artificial intelligence app for pill count, Parkinson's tracker app, education app. | Searches:  1990-2018  Publications:  2014-2018 | 9 studies  RCTs | Clinical population:  cardiovascular disease (n=5); depression (n=1); Parkinson’s disease (n=1); psoriasis (n=1); multimorbidity (n=1)  n=1,159* | Medication adherence | *Main effects, intervention versus control:*   - Medication adherence: (OR 2.120; 95% CI 1.635 to 2.747; I^2^= 9.8%; k=9). | *Meta regression:*  Those coded in more than 3, but less than 6 of the 9 studies were included in the meta-regression   - no significant associations between the BCTs used and the effect size. | - Tailored (k=9) - 7.1 Prompts/cues (k=7) - Report whether or not the behavior was performed’ (k=6). - Monitoring of behavior by others without feedback (k=unclear) - 2.2 Feedback on behavior (k=unclear) - 3.1 Social support (unspecified) - Habit formation (k=unclear) - Goal setting (k=unclear) | *Cochrane Collaboration’s Risk of Bias:*   - Overall, all studies were deemed as unclear in their risk of bias. - The most common sources of risk of bias were the absence of blinding of participants and personnel to the outcome measure and a lack of objective measurements of medication adherence. | **Moderate**  No critical flaws  More than one non-critical weakness (excluded studies not listed, no explanation for selection of study designs, no information on funding source, publication bias reported but not discussed) |
| Palmer 2018 [100]  Systematic review | mHealth  Any mobile phone-specific delivery mechanism, including short messaging service (SMS), multimedia messaging (MMS), applications (apps) and Interactive Voice Response. | Searches: Inception to 2017  Publications:  2012-2017 | 4 studies  RCTs | Clinical population: Adults who have been prescribed medication for the primary prevention of CVD  N=2,429 | Medication adherence | *Main effect: Not reported*  *Other relevant findings:*   - There is low-quality evidence relating to the effects of mobile phone-delivered interventions to increase adherence to medication prescribed for the primary prevention of CVD; some trials reported small benefits while others found no effect. - On the basis of this review, there is currently uncertainty around the effectiveness of these interventions. | *Quantitative analysis: Not reported* | - 2.2 Feedback on behavior (k=3) - 5.1 Information about health consequences (k=3) - 5.2 Salience of consequences (k=3) | *Cochrane Collaboration’s Risk of Bias:*   - All studies had low to unclear risk of bias across domains, with the exception of one study that had high risk of bias of performance bias. | **High**  No critical flaws or non-critical weaknesses |
| Pouls 2021 [101]  Systematic Review | eHealth  Mobile apps, monitoring devices, SMS text messages or IVR interventions, e-training modules through an online portal. | Searches:  2014-2019  Publications:  2014-2019 | 21 studies  RCTs | Clinical population: adults with chronic preventive maintenance medication  N=10,464 | Medication adherence | *Main effect: Not reported*  *Other relevant findings:*   - Overall, 17/21 interventions yielded a statistically significant improvement of medication adherence compared to the control group. - For 14 of these interventions an effect size (Cohen d) could also be calculated; 2 interventions reported a large effect size (Cohen d ≥ 0.8), 4 had a medium effect size (Cohen d ≥ 0.5 < 0.8), 3 had a small effect size (Cohen d ≥ 0.2 < 0.5), and 5 interventions had a negligible effect size (Cohen d < 0.2). - For the remaining 3 interventions an OR could be calculated which showed strong odds for becoming adherent in the intervention group (OR ≥ 2.0). | *Quantitative analysis: Not reported*  *Other relevant findings:*   - There was strong evidence for a positive effect of strategies to teach skills [4.1 Instruction on how to perform a behaviour], to facilitate communication or decision-making, and to improve health care quality. - For all other intervention strategies (e.g., to support, to inform, and educate) there was conflicting evidence. | - 5.1 Information about health consequences [education] (23/29, 79%) - 3.1 Social support (unspecified) (15/29, 52%) | *Cochrane Collaboration’s Risk of Bias:*   - 15/21 studies had a positive score on at least five domains and were regarded high-quality studies - 2 studies had the lowest score with 2 out of 7 domains scored as positive. - The most common sources of risk of bias were the absence of blinding of participants and personnel | **Moderate**  No critical flaws  More than one non-critical weakness (no justification for selection of study design, excluded studies not listed, no information on funding sources, no discussion of heterogeneity) |
| Bond 2021 [102]  Systematic review | mHealth  Automated phone messages including interactive voice recognition, electronic reminder device, text messages, phone calls, partner support, non-digital pill boxes, in person consultation, email, web portal, micro letter, and apps. | Searches:  2000-2020  Publications: 2006-2020 | 17 studies reported in 20 papers  RCTs | Clinical population: Patients of any age who were prescribed statins in any setting for the primary or secondary prevention of CVD. | Medication adherence for primary or secondary prevention of CVD | *Main effect: Not reported*  *Other relevant findings:*   - 12/17 studies (71%) reported a statistically significant improvement on participant adherence to statin medication for those using mHealth interventions compared to usual care - If these 12, 7 reported effect sizes ranging from 0.06 to 0.75 - The relative improvement in adherence ranged from 2% to 63%, with 5 studies (29%) demonstrating less than 10% relative improvement, 3 studies (18%) between 10–25%, and 6 studies (35%) over 25% relative improvement | *Quantitative analysis: Not reported* | - 7.1 Prompts/cues (k=16; 11 effective studies, 69%) - 5.1 Information about health consequences (k=12; 9 effective studies, 75%) - 12.5 Adding objects to the environment (k=10; 6 effective studies, 60%)   Of the BCTs coded in more than 3 studies, the BCTs with the highest proportion of successful interventions was:   - 1.1 Goal setting (behaviour) (k=3; 3 effective studies, 100%) - 4.1 Instruction on how to perform a behaviour (k=3; 3 effective studies, 100%) - 9.1 Credible source (k=9; 7 effective studies, 78%).   and the lowest proportion:   - 1.2 Problem solving (k=6, 4 effective studies, 67%) - 2.3 Self-monitoring of behaviour (k=3; 2 effective studies, 67%). | *Cochrane Risk of Bias tool Version 2 (RoB2):*   - 4 studies were determined as having “some concerns”. - 13 studies had a “high” overall risk of bias. - No included studies were deemed to have a “low” risk. - “Deviations from intended intervention” had the greatest proportion of “high” risk scores. - Participant engagement with the intended intervention was not sufficiently assessed and accounted for in 9 studies. | **Low**  One critical flaw (no pre-registered protocol)  More than one non-critical weakness (excluded studies not listed, no explanation for selection of study designs, study selection and data extraction not done in duplicate, no information on funding sources) |
| Donovan 2022 [103]  Systematic review | mHealth a  Automated two-way digital communication technologies including Interactive Voice Response or text messaging using SMS or pagers | Searches:  Up to 2020  Publications:  1996-2020 | 36 studies reported in 43 papers  RCTs, NRCTs, cohort studies, case-control studies | Clinical population: adults who were self-managing their medication for any long-term condition in their own home  N=38,509 | Medication adherence | *Main effect: Not reported*  *Other relevant findings:*   - 19/34 studies found improvement in medication adherence - 7/34 studies had unclear findings on medication adherence - 8/34 studies found no improvement on medication adherence - 7/20 studies reported improved clinical outcomes - 9/20 studies reported no improvement in clinical outcomes and 4/20 had unclear findings, even if there were improvements in medication adherence | *Quantitative analysis: Not reported*  *Other relevant findings:*  Medication adherence was improved in:   - 90% of studies using BCTs to target obtaining medication - 5/11 studies using Prompts/cues - 60% of studies including Social Reward - 43% of studies providing feedback - 38% of studies which monitored medication taking without feedback - 7/8 studies delivering Problem Solving - 50% of studies including Information about health consequences | - 7.1 Prompts/Cues (k= 19) - 2.1 Monitoring of behavior by others without feedback (k=19) - 4.1 Instruction on how to perform a behavior | *Mixed-Methods Appraisal tool:*  Most of the RCTs were of good quality, usually only missing the ‘lack of allocation concealment’ criteria. Patient concealment is not possible with this type of intervention; however, some described concealment of investigators. | **Critical Low**  More than one critical flaw (unsatisfactory risk of bias assessment, no consideration of bias when interpreting results)  More than one non-critical weakness (excluded studies not listed, no justification for selection of study design, no information on funding sources, no discussion of heterogeneity) |
| Substance Use |  |  |  |  |  |  |  |  |  |  |
| Kaner 2017 [27]  Systematic Review and meta-analysis | eHealth and mHealth  Computer or mobile device (laptop, phone or tablet) responsive to user input to generate personalized content which aimed to change the participants’ alcohol-related behaviors. Interventions were not restricted to those accessible online. | Searches:  up to 2017  Publications  1997-2015 | 57 studies reported in 55 papers  RCTs | General population  N=34,390 | Alcohol-related behaviors | *Main effects, intervention versus control or minimal intervention:*   - Quantity of alcohol consumed per week: 22.8g (95% CI 15.4 to 30.3; I^2^ = 78%; k=41) of alcohol per week less than control group participants at the longest reported follow-up point. - Frequency of drinking per week: estimated difference -0.16 days drinking per week (95% CI -0.24 to -0.09; I^2^=38.64%; k=15), which equates to less than one day fewer drinking per month linked to a digital intervention. - Frequency of binges per week: estimated difference -0.24 (95% CI -0.35 to -0.13; I^2^=53.16%; k=15), which equates to about one binge fewer per month linked to a digital intervention. - Intensity of drinking: estimated difference -4.63 g/alcohol per drinking day (95% CI -8.02 to -1.23; I^2^=83.13%; k=15), which equates to less than one unit fewer per drinking day. - Binge drinkers: the risk ratio of being a binge drinker at the time of longest follow-up among those randomised to a digital intervention relative to those randomised to a control or minimal intervention condition was 0.98 (95% CI 0.97 to 1.00; I^2^=0%; k=9).   *NB. Sub-group and sensitivity analyses are not reported here.* | *Meta regression:*  BCTs associated with reduced alcohol consumption in unadjusted models:   - Goal setting (B -43.94, SE 17.14, 95% CI -78.59 to -9.30, P = 0.01, R^2^adj 6.64%) - 1.2 Problem solving (B -48.03, SE 14.72, 95% CI -77.79 to -18.27, P < 0.01, R^2^adj 25.01%) - 4.2 Information about antecedents (B -74.20, SE 21.53, 95% CI -117.72 to -30.68, P < 0.01, R^2^adj 32.15%) - 8.2 Behavior substitution (B -123.71, SE 30.14, 95% CI -184.63 to -62.80, P < 0.001, R^2^adj 48.53%) - 9.1 Credible source (B -39.89, SE 16.22, 95% CI -72.66 to -7.11, P = 0.02, R^2^adj 15.60%).   BCTs associated with reduced alcohol consumption in adjusted models that included BCTs with B > 23 in the unadjusted model:   - 8.2 Behaviour substitution (B -95.12, SE 33.09, 95% CI -162.90 to -27.34, P = 0.01) - 1.2 Problem solving (B -45.92, SE 21.99, 95% CI -90.97 to -0.87, P = 0.05) - 9.1 Credible source (B -32.09, SE 13.94, 95% CI -60.64 to -3.55, P = 0.03) | Of the BCTs uniquely present in experimental  arms, the  five most frequently used were:   - 2.2 Feedback on behavior (85.7%, k=36) - 6.2 Social comparison (81.0%, n=34) - 5.3 Information about social and environmental consequences (71.4%, k=30) - 2.7 Feedback on outcomes of behavior (69.0%, k=29) - 3.1 Social support (unspecified) (64.3%, k=27) | *Cochrane Collaboration’s Risk of Bias:*   - One study had low risk of bias across all domains. - The remaining studies had unclear or high risk of bias in one or more domain. - High risk of bias was predominantly identified for blinding and incomplete outcome data. Removing high risk trials from the analysis resulted in slightly smaller, but still significant, effect sizes.   Potential publication bias was identified for the main analysis (quantity of alcohol per week) suggesting under-reporting of results with little or no evidence  of an intervention effect. | **High**  No critical flaws  One non-critical weakness (no explanation for selection of study designs) |
| Griffiths 2018 [104]  Systematic review and meta-analysis | eHealth  Computer (PC or laptop), video or DVD, mobile telephone or portable handheld device (e.g., tablet or iPad). This included email, video, DVDs, websites or web-based games, mobile or tablet applications and SMS text messages or MMS multimedia messages. | Searches:  up to 2017  Publications:  1991-2017 | 12 studies  RCT and quasi-randomized controlled trial | General population: Women aged 16 years or older at any stage of pregnancy, reporting to be current cigarette smokers  N=2,970 | Smoking cessation during pregnancy | *Main effects, intervention versus control:*   - Odds of quitting smoking during pregnancy (OR 1.44; 95%CI 1.04 to 2.00; p= .03; I^2^= 17.7%; k=12) - The effect estimate favoured the control group in three trials. | *Subgroup analyses* BCTs significantly associated with the effectiveness of digital interventions for smoking cessation in pregnancy:   - 4.2 Information about antecedents (OR = 2.06, 95% CI 1.25 to 3.41; *p=*0.01) - 1.4 Action planning (OR = 1.97, 95% CI 1.27 to 3.05; *p<*0.01) - 1.2 Problem solving (OR = 1.75, 95% CI 1.25 to 2.46; *p<* 0.01) - 1.1 Goal setting (behavior) (OR = 1.71, 95% CI 1.22 to 2.41; *p<*0.01) - 1.5 Review behavior goals (OR = 1.69, 95% CI 1.16 to 2.45; *p*=0.01) - 3.1 Social support (unspecified) (OR = 1.63, 95% CI 1.14 to 2.33; *p*=0.01) - 9.2 Pros and cons (OR = 1.61, 95% CI 1.09 to 2.39; *p*=0.02)   *Meta-regression*   - Interventions using a larger number of BCTs produced a greater effect (coefficient of 0.11 (SE 0.05; 95% CI 0.02 to 0.19; p= 0.02). | - 1.2 Problem solving (k=6) - 1.1 Goal setting (behavior) (k=5) - 1.4 Action planning (k=5) - 2.3 Self-monitoring of behavior (k=5) - 3.1 Social support (unspecified) (k=5) - 4.2 Information about antecedents (k=5) - 1.5 Review behavior goals (k=4) - 6.1 Demonstration of the behavior (k=4) - 9.2 Pros and cons (k=4) - 12.5 Adding objects to the environment (k = 4). | *Cochrane Collaboration’s Risk of Bias:*   - Many studies had a high risk of bias on one or more key domains (*k*=7), with high risk most commonly assigned for incomplete intervention implementation. - One study was found to have a low risk of bias across all domains - Four studies had an overall unclear risk of bias.   The funnel plot revealed possible publication bias and missing unpublished trials with negative effects. | **Moderate**  No critical flaws  More than one non-critical weakness (no explanation for selection of study designs, insufficient detail on included studies, no information on funding sources). |
| McCrabb 2019 [105]  Systematic review and meta-analysis | eHealth  Solely smoking cessation programs delivered via the internet (including combinations of internet-based interventions with additional  support such as smoking cessation medication and telephone support calls; interventions which include social  media or social support components; and excluding those  interventions which target multiple health risk behaviors  with a smoking cessation component) | Searches:  Up to 2017  Publications:  2005-2017 | 45 studies  RCTs | General population: Tobacco smokers  N=65,736 | Smoking abstinence | *Main effects, intervention versus comparison:*  Short term effects (<6 months):   - Smoking cessation (all outcome measures; overall OR 1.29; 95% CI 1.12 to 1.50; p= .001; I^2^ = 81.7%; k=34). - “prolonged abstinence” (OR 1.43; 95%CI 1.09 to 1.87; p= .009) - “30-day point prevalence abstinence” (OR 1.75; 95% CI 1.13 to 2.72; p= .013).   Long-term effects (≥ 6 months):   - Smoking cessation (all outcome measures; overall OR, 1.19, 95% CI = 1.06, 1.35, *p* = 0.004; I^2^ = 56.8%; k=31) - “prolonged abstinence” (OR = 1.40, 95% CI = 1.19, 1.63, *p* < .001 - No long-term effects found for “30-day point prevalence abstinence” or 7-day point prevalence abstinence. | *Meta-regression*   - No association between the difference in the number of BCTs between intervention and control arms and intervention effectiveness in the short term (OR = 1.01, 95% CI 0.98, 1.04, p = .556).   BCTs effective at increasing short-term effectiveness:   - 1.1 Goal setting [behavioral], - 1.2 Problem solving - 1.4 Action planning - 3.1 Social support [unspecified], - 5.1 Information about health consequences, - 9.2 Pros and cons, and - 11.1 Pharmacological support - The difference in the number of BCTs in the long term was not significantly associated with treatment effectiveness (OR = 1.02, 95% CI 0.99, 1.05, *p* = 0.16)   BCTs found to be associated with increased program effectiveness in the long term.   - 1.2 Problem solving - 1.4 Action planning - 3.1 Social support [unspecified] - 5.1 Information about health consequences - 9.2 Pros and cons - 11.1 Pharmacological support | In the intervention groups;   - 1.2 Problem solving (71.7%) - 3.1 Social support [unspecified] (67.5%) - 11.1 Pharmacological support (63.0%) - 1.1 Goal setting [behavior] (56.5%) | *Cochrane Collaboration’s Risk of Bias:*   - Overall, most studies were rated as low risk of bias (n = 30); however, approximately a quarter (n = 11) rated as unclear due to lack of information or high on each domain. - A total of four studies were rated as having an overall high risk of bias. - Funnel plots showed there may be publication bias for studies reporting short-term outcomes; however, this does not appear to be the case for the studies reporting long-term outcomes. This was confirmed using Egger’s test of bias (short-term p = .003, long-term p = .151). | **Low**  One critical flaw (no consideration of bias when interpreting results)  More than one non-critical weakness (no justification for selection of study design, excluded studies not listed, no information on funding sources, no assessment of impact of risk of bias, no discussion of heterogeneity) |
| Howlett 2022 [106]  Systematic review | eHealth and mHealth  Computer or mobile devices (e.g., laptops or smartphones) and being specific to the service user (not readily available libraries of content). Interventions could be asynchronous or synchronous. | Searches:  Up to 2020  Publications:  2008-2019 | 62 studies  RCTs, NRCTs, quasi-randomised trials, and natural experimental studies (pre- and post-studies, interrupted time series studies) | Clinical population: adults experiencing alcohol and/or substance misuse  N= *Not reported* | Behaviour change related to alcohol and/or substance misuse | *Main effect: Not reported*  *Other relevant findings:*   - 19 studies (34%) were rated as very promising - 23 studies (42 %) as quite promising - 13 studies (24 %) as not promising   (7 studies utilising single group pre-post designs were not included in the promise ratings calculations as a very promising rating was not possible) | Effectiveness:  *Sensitivity analysis of promise ratios removing studies with high risk of bias*  *For studies targeting alcohol misuse the most promising BCTs were:*   - 2.3 Self-monitoring of behaviour (7:1 promise ratio) - 12.3 Avoidance/ reducing exposure to cues for behaviour (5:1 promise ratio) - 9.2 Pros and cons (5:1 promise ratio) - 8.1 Behaviour practice/rehearsal (2:1 promise ratio) - 9.1 Credible source (2:1 promise ratio)   *For studies targeting substance misuse the most promising BCTs were:*   - 1.2 Problem Solving (2:1 promise ratio) - 2.3 Self-monitoring of behaviour (2:1 promise ratio) | *For studies targeting alcohol misuse (k=52):*   - 2.2 Feedback on behaviour (*k*=27) - 1.2 Problem solving (*k*=25) - 1.1 Goal setting (behaviour) (*k*=23)   *For studies targeting substance misuse (k=10):*   - 2.2 Feedback on behaviour (*k*=6) - 1.2 Problem solving (k=5) - 2.3 Self-monitoring of behaviour (*k*=5) - 1.1 Goal setting (behaviour) (*k*=5) | *The Cochrane Risk of Bias (RoB2) tool:*   - All of the 53 RCTs had either some concerns (*k* = 39) or were judged to have high risk of bias (*k* = 14) - The risk of bias domains judged to have the most high-risk studies were ‘Randomisation process’ (6/53 studies judged low risk) and ‘Selection of the reported result’ domain (6/53 studies judged high risk) | **Moderate**  No critical flaws  More than one non-critical weakness (no explanation for selection of study designs, excluded studies not listed). |
| Getty 2019 [107]  Meta-analysis | mHealth  Mobile telephones | Searches:  1995-2019  Publications:  2013-2018 | 7 studies  RCT and within-subject design studies | Mixed: frequent drinkers, smokers, HIV and substance misusers  N=222 | Substance use – Drugs, tobacco, alcohol | *Main effects, intervention versus control:*   - Percentage of negative samples: pooled effect size d= 0.94 (95% CI 0.63 to 1.25; I^2^=6%; k=5), with contingency management performing better than the non- contingency management condition. - Quit rate: pooled effect size d= 0.46 (95% CI 0.27 to 0.66; I^2^=0%; k=2), with contingency management performing better than the non- contingency management condition. - Longest duration abstinent: pooled effect size of d= 1.08 (95% CI 0.69 to 1.46; I^2^=0%; k=2; n=119), with contingency management performing better than the non- contingency management condition. - Mobile telephone delivered contingency management performs significantly better than control conditions in reducing tobacco and alcohol use among adults not in treatment for substance use disorders. | *Quantitative analysis: Not reported* | - 10.1 Material incentives [monetary] (n=6), - Escalating schedule of reinforcement whereby the amount of reinforcement increased progressively following consecutive achievement (n=7) - 14.8 Reward alternative behavior [to reinforce abstinence] (n=unknown) | *Quality Assessment Tool for Quantitative Studies:*   - Overall, most of the retrieved studies had a high quality of data collection and reporting withdrawals/dropouts. - None of the studies were double-blinded, as blinding both participants and providers to contingency management interventions is not possible due to the nature of the intervention. - All studies employing a RCT design included details regarding the method used to randomize participants.   Studies employing a within subjects design were rated as being of moderate quality as per guidelines from the Effective Public Health Practice Project (EPHPP) quality assessment tool. | **Critical Low**  More than one critical flaw (appropriateness of meta-analytic method unclear, no consideration of bias when interpreting results)  More than one non-critical weakness (missing information on study population inclusion criteria, no explanation for selection of study designs, data extraction not done in duplicate, excluded studies not listed, insufficient detail on included studies, no information on funding sources, no assessment of impact of risk of bias, no discussion of heterogeneity) |
| Humphreys 2021 [111]  Systematic review | eHealth  Entirely via the web | Searches:  2009-2019  Publications:  2008-2019 | 45 studies  RCT or case-controlled trials | General population: At-risk groups  N=32,201 | Behaviour change related to alcohol use, binge eating, or gambling | *Main effect: Not reported*  *Other relevant findings:*   - 66% (21/32) of alcohol interventions, 83% (5/6) of eating behavior interventions, 43% (3/7) of gambling interventions were effective. | *Quantitative analysis: Not reported*  *Other relevant findings:*  Present in effective high-quality studies:   - 2.2 Feedback on behavior - 2.3 Self-monitoring of behavior - 2.4 Self-monitoring of outcomes of behavior - 4.1 Instruction on how to perform the behavior. - 6.2 Social comparison | - 2.3 Self-monitoring of behaviour (k=39) - 2.2 Feedback on behaviour (k=30) - 6.2 Social comparison (k=27) - 5.3 Information about social and environmental consequences (k=26) - 4.1 Instruction on how to perform a behavior (k=25) - 1.2 Problem solving (k=25) | *Office of Health Assessment and Translation (OHAT) Risk of Bias Rating Tool:*   - 56% (25/45) of papers were rated as having high quality (OHAT>70%) | **Moderate**  No critical flaws  More than one non-critical weakness (no explanation for selection of study designs, excluded studies not listed, no information on funding sources, no discussion of heterogeneity) |
| Staiger 2020 [108]  Systematic review | mHealth  Mobile app (not web-based or SMS in isolation). Most apps were stand alone, but some had additional adjunct components such as supportive counseling; motivational interviewing; educational messages; links to resources; peer group supports such as Facebook groups; nicotine replacement therapy; audio-guided relaxation; and even a high-risk patient locator, which sends an alert to patients if they are approaching a high-risk drinking location. | Searches:  2007-2019  Publications:  2013-2018 | 20 studies  RCT or matched control trial | General population and clinical population: community, university / college students, and clinical or dependent patients  N=16,133* | Substance use – Drugs, tobacco, alcohol | *Main effect: Not reported*  *Other relevant findings:*   - 6/20 app interventions reported significant reductions in substance use at post or follow-up compared with a comparison condition, with small to moderate effect sizes. | *Quantitative analysis: Not reported*  *Other relevant findings:*   - 3/6 effective apps included normative feedback, and 1 app included personalized feedback (actual consumption compared with goals). | - 2.3 Self-monitoring (behavior) - Personalised feedback - Psychoeducation | *Cochrane Collaboration’s Risk of Bias:*   - 3 studies had low risk of bias across all domains. - The remaining studies had unclear or high risk of bias for one or more domains. - High risk of bias was most commonly found for blinding of participants and personnel. | **Low**  One critical flaw (no pre-registered protocol)  More than one non-critical weakness (missing information on inclusion criteria for population, no justification for selection of study design, unclear whether data extraction was done in duplicate, excluded studies not listed, no information on funding sources, no discussion on heterogeneity) |
| Perski 2022 [109]  Systematic review | eHealth and mHealth  Websites, text messages, apps, and wearables | Searches:  2000-2020  Publications:  2007-2020 | 14 studies  RCTs, pilot RCT, NR pilot studies, mixed method studies | Clinical population and general population  N=1,154* (experimental studies only) | Reduction in urges, smoking cessation, alcohol reduction | *Main effect: Not reported*  *Other relevant findings:*   - 2/2 medium-sized RCTs found mixed results for alcohol consumption - 5/5 small-sized pilot RCTs found mixed results for smoking, alcohol consumption, and illicit substance use - 5/5 small-sized single- or two-arm non-randomized pilot studies reported mixed results for smoking, alcohol consumption, and cannabis use | *Quantitative analysis: Not reported* | - 7.1 Prompts/cues (k=14; 100%) - 3.1 Social support (unspecified) k=6; 43%) - 12.3 Avoidance/ reducing exposure to cues for the behaviour (k=3; 21%) - Personalisation (k=14; 100%) | *mHealth Evidence Reporting and Assessment (mERA) checklist:*   - None of the 14 studies reported full details for all 16 quality criteria. - 5/ 14 studies reported full details on intervention delivery or user feedback. - Many studies reported insufficient details on either infrastructure, interoperability, usability testing, access of individual participants, cost assessment, limitations for delivery at scale, contextual adaptability, replicability, data security, compliance with national guidelines and fidelity of delivery. | **Critical Low**  More than one critical flaw (no risk of bias assessment, no consideration of bias when interpreting results)  More than one non-critical weakness (excluded studies not listed, insufficient details on included studies, no information on funding sources) |
| Lehto 2011 [110]  Systematic review | eHealth  Web-based;  all aimed at persuading the users in some way | Searches: 2004-2009  Publications:  2004-2009 | 23 studies  RCTs or quasi-experimental design | *Not reported* | Tobacco or alcohol related behaviour | *Main effect: Not reported*  *Other relevant findings:*   - 12/20 studies reported statistically significant between group differences in health behaviours. - For smoking cessation, 4 trials reported significant effects in favour of interventions groups compared to control. - For alcohol interventions, 2 reported positive effects in interventions groups compared to control. | *Quantitative analysis: Not reported* | ‘Primary task’ category persuasion techniques  Included:   - 8.7 Graded tasks [reduction] (k=23) - 2.3 Self-monitoring (behaviour) (k=23) - 9.3 Comparative imagining of future outcomes [simulation] (k=14) - personalization (k=13), - tailoring (k=8) - tunnelling (k=4)   ‘Dialogue support’ category:   - 7.1 Prompts/cues [reminders] (k=10) - 9.1 Credible source [ask-an-expert service] (k=9) - Suggestion (k=4) - Similarity (k=4)   ‘Social support’ category:   - Asynchronous peer discussion forum - Synchronous chat rooms. | *CONSORT 2010 checklist:*   - There were some concerns with the quality of 4 studies but they were still included.   No quality assessment or risk of bias reported. | **Critical Low**  More than one critical flaw (no pre-registered protocol, no risk of bias assessment, no consideration of bias when interpreting results)  More than one non-critical weakness (missing information on inclusion criteria for population and comparator, no justification for selection of study design, study selection and data extraction not done in duplicate, insufficient detail on included studies, no information on funding sources, no discussion of heterogeneity) |

*Total sample size was calculated from data available in the paper and only in cases where sample sizes were reported for every included study. App = application; BG = blood glucose; BMI = body mass index; BP = blood pressure; CHD = coronary heart disease; CI= confidence interval; CVD = cardiovascular disease; DBCI = digital behavior change intervention; DBP = diastolic blood pressure; GRADE = Grading of Recommendations Assessment, Development and Evaluation GWG = gestational weight gain; HbA1c = glycated haemoglobin; HDL-C = high density lipoprotein cholesterol; HRQoL = health-related quality of life; I^2^ = heterogeneity statistic; ITT = intention-to-treat; LDL-C = low density lipoprotein cholesterol; MD = mean difference; MET = metabolic equivalent; MVPA = moderate-to-vigorous physical activity; NCD = non-communicable disease; NRCTs = non-randomised controlled trials; OR= odds ratio; PA = physical activity; PDA = personal digital assistant; QoL = quality of life; RCT = randomised controlled trial; RoB = Risk of Bias; SBP = systolic blood pressure; SMD= standardised mean difference; SMS = short messaging service; TAU = treatment as usual; TC = total cholesterol; TG = triglyceride. Significance is considered p<0.05.
