# Supplementary material for: Effective Behavior Change Techniques in Digital Health Interventions for the Prevention or Management of Noncommunicable Diseases: An Umbrella Review
Source: Ann Behav Med. 2023 Aug 25;57(10):817–35. doi: 10.1093/abm/kaad041 (PMC10498822; doi:10.1093/abm/kaad041)
Supplement: kaad041_suppl_Supplementary_File_2 [file kaad041_suppl_supplementary_file_2.docx]

***Appendix 1:*** Search strategy for the Embase database.

| **Search terms** ^[[1]](#footnote-1)^ | **Description** |
| --- | --- |
| #1 'wireless technology'/en  #2 'smartphone'/en  #3 'internet'/en  #4 (computer$ NEAR/3 (handheld OR 'hand held' OR mobile)):ab,ti  #5 (tablet$ NEAR/1 (computer$ OR pc$)):ab,ti  #6 'ehealth':ab,ti OR 'e-health':ab,ti OR 'electronic-health':ab,ti OR 'e-intervention':ab,ti  #7 'web-based':ab,ti OR 'webbased':ab,ti OR 'internet-delivered treatment':ab,ti  #8 'digital health':ab,ti  #9 'cell phone'/en  #10 (((mobile OR cell* OR smart) AND phone$) OR smartphone):ab,ti  #11 (mhealth OR m-health OR mobile-health):ab,ti  # 12 (mobile AND app*):ab,ti #13 #1 OR #2 OR #3 OR #4 OR #5 OR #6 OR #7 OR #8 OR #9 OR #10 OR #11 OR #12 | Terms for mHealth/eHealth applications |
| 14 'behaviour change':ab,ti OR 'behaviour change':ab,ti  #15 'active ingredient*':ti OR 'active element*':ti OR 'intervention component*':ti OR 'intervention function*':ti OR 'process variable*':ti OR 'mediat*':ti OR 'mechanism*':ti OR 'mechanism* of therapy':ti OR 'change mechanism*':ti OR 'therapeutic change':ti OR 'indirect effect*':ti OR 'working function':ti OR 'active ingredient*':ab OR 'active element*':ab OR 'intervention component*':ab OR 'intervention function*':ab OR 'process variable*':ab OR 'mediat* of change':ab OR 'mechanism* of change':ab OR 'mechanism* of therapy':ab OR 'change mechanism*':ab OR 'therapeutic change':ab OR 'indirect effect*':ab OR 'working function':ab  #16 #14 OR #15  #17 #13 AND #16 | Terms for behaviour change techniques |
| #18 'review'/it  #19 ((hand NEAR/2 search*):ab,ti) OR ((manual* NEAR/2 search*):ab,ti)  #20 "electronic database*":ab,ti OR "bibliographic database*":ab,ti OR "computeri?ed database*":ab,ti OR "online database*":ab,ti  #21 #19 OR #20  #22 #17 AND #18 OR #21  #23 'meta analysis'/exp  #24 'meta-analys*':ti,ab OR 'meta analys*':ti,ab OR 'metaanalys*':ti,ab 🡪subject heading  #25 (systemati* NEAR/5 (review* OR overview)):ab,ti  #26 (quantitative* NEAR/5 (review* OR overview OR synthes*)):ab,ti  #27 (methodologi* NEAR/5 (review* OR overview$)):ab,ti  #28 "integrative research review":ab,ti OR 'research integration':ab,ti  #29 #23 OR #24 OR #25 OR #26 OR #27 OR #28  #30 #22 AND #29 | Terms for systematic reviews |
| #31 #30 AND [2007-2021]/py | Publication period |

For details on the abbreviations/field codes used, see[:https://ospguides.ovid.com/OSPguides/embase.htm](https://ospguides.ovid.com/OSPguides/embase.htm)

1. For details on the abbreviations/field codes used, see[:https://ospguides.ovid.com/OSPguides/embase.htm](https://ospguides.ovid.com/OSPguides/embase.htm) [↑](#footnote-ref-1)
