# Supplementary material for: Effective Behavior Change Techniques in Digital Health Interventions for the Prevention or Management of Noncommunicable Diseases: An Umbrella Review
Source: Ann Behav Med. 2023 Aug 25;57(10):817–35. doi: 10.1093/abm/kaad041 (PMC10498822; doi:10.1093/abm/kaad041)
Supplement: kaad041_suppl_Supplementary_File_3 [file kaad041_suppl_supplementary_file_3.docx]

Supplementary File 3. Excluded studies with reasons for exclusion.

| Amanvermez 2022 | Wrong health domain (i.e. not NCDs) |
| --- | --- |
| Adler 2017 | No BCTs/process variables/intervention components/active elements reported |
| Aguiar 2022 | Wrong focus (i.e. App design/development, content analyses, user satisfaction, technical details) |
| Al-Durra 2015 | No effectiveness indices reported (e.g. only frequency of BCTs) |
| Alhasani 2022 | No effectiveness indices reported (e.g. only frequency of BCTs) |
| Ang 2021 | No BCTs/process variables/intervention components/active elements reported |
| Arigo 2020 | No effectiveness indices reported (e.g. only frequency of BCTs) |
| Aromatario 2019 | No effectiveness indices reported (e.g. only frequency of BCTs) |
| Bardus 2015 | No effectiveness indices reported (e.g. only frequency of BCTs) |
| Bardus 2016 | No effectiveness indices reported (e.g. only frequency of BCTs) |
| Belisario 2013 | No BCTs/process variables/intervention components/active elements reported |
| Bossen 2014 | No BCTs/process variables/intervention components/active elements reported |
| Brouwer 2011 | No effectiveness indices reported (e.g. only frequency of BCTs) |
| Bunova 2022 | No effectiveness indices reported (e.g. only frequency of BCTs) |
| Bustamante 2021 | No effectiveness indices reported (e.g. only frequency of BCTs) |
| Cao 2022 | No effectiveness indices reported (e.g. only frequency of BCTs) |
| Caponnetto 2021 | No BCTs/process variables/intervention components/active elements reported |
| Carey 2009 | No BCTs/process variables/intervention components/active elements reported |
| Chaudhry 2020 | No BCTs/process variables/intervention components/active elements reported |
| Cheatham 2018 | No BCTs/process variables/intervention components/active elements reported |
| Chen 2012 | No BCTs/process variables/intervention components/active elements reported |
| Cho 2018 | No effectiveness indices reported (e.g. only frequency of BCTs) |
| Chu 2021 | No BCTs/process variables/intervention components/active elements reported |
| Cole-Lewis 2010 | No BCTs/process variables/intervention components/active elements reported |
| Corbett 2018 | No effectiveness indices reported (e.g. only frequency of BCTs) |
| Coulon 2016 | No effectiveness indices reported (e.g. only frequency of BCTs) |
| Cugelman 2011 | Wrong health domain (i.e. not NCDs) |
| DeLeon 2014 | No effectiveness indices reported (e.g. only frequency of BCTs) |
| Dettore 2015 | Wrong health domain (i.e. not NCDs) |
| Domhardt 2019 | Wrong health domain (i.e. not NCDs) |
| Domhardt 2020 | Wrong health domain (i.e. not NCDs) |
| Domhardt 2021 | Wrong health domain (i.e. not NCDs) |
| Duan 2021 | No BCTs/process variables/intervention components/active elements reported |
| Edwards 2016 | No effectiveness indices reported (e.g. only frequency of BCTs) |
| Eisenstadt 2021 | Wrong health domain (i.e. not NCDs) |
| Elaheebocus 2018 | Wrong focus (i.e. App design/development, content analyses, user satisfaction, technical details) |
| Enwald 2010 | No BCTs/process variables/intervention components/active elements reported |
| Faessen 2022 | No effectiveness indices reported (e.g. only frequency of BCTs) |
| Finch 2016 | No BCTs/process variables/intervention components/active elements reported |
| Gan 2022 | Wrong focus (i.e. App design/development, content analyses, user satisfaction, technical details) |
| Gosak 2021 | Wrong focus (i.e. App design/development, content analyses, user satisfaction, technical details) |
| Grace-Farfaglia 2019 | No effectiveness indices reported (e.g. only frequency of BCTs) |
| Greenwell 2016 | Wrong health domain (i.e. not NCDs) |
| Gurman 2012 | Wrong health domain (i.e. not NCDs) |
| Hayman 2021 | No effectiveness indices reported (e.g. only frequency of BCTs) |
| Hedaoo 2017 | No full-text |
| Howarth 2018 | No BCTs/process variables/intervention components/active elements reported |
| Hrynyschyn 2021 | No BCTs/process variables/intervention components/active elements reported |
| Islam 2022 | No effectiveness indices reported (e.g. only frequency of BCTs) |
| Jagroep 2022 | E/M-Health application not primary intervention |
| Johnson 2022 | No BCTs/process variables/intervention components/active elements reported |
| Kamalumpundi 2022 | Wrong health domain (i.e. not NCDs) |
| Keller 2022 | No BCTs/process variables/intervention components/active elements reported |
| Lavoie 2022 | No effectiveness indices reported (e.g. only frequency of BCTs) |
| Lee 2018 | No effectiveness indices reported (e.g. only frequency of BCTs) |
| Lyons 2014 | No effectiveness indices reported (e.g. only frequency of BCTs) |
| Manning 2022 | No full-text |
| Martín-Martín 2021 | No effectiveness indices reported (e.g. only frequency of BCTs) |
| McKay 2018 | No effectiveness indices reported (e.g. only frequency of BCTs) |
| McLaughlin 2021 | No BCTs/process variables/intervention components/active elements reported |
| McMillan 2017 | No BCTs/process variables/intervention components/active elements reported |
| Melendez-Torres 2022 | No BCTs/process variables/intervention components/active elements reported |
| Messiah 2020 | No BCTs/process variables/intervention components/active elements reported |
| Middelweerd 2014 | No effectiveness indices reported (e.g. only frequency of BCTs) |
| Mogoa»ôe 2017 | Wrong health domain (i.e. not NCDs) |
| Moulton-Perkins 2022 | No full-text |
| Musiat 2022 | Wrong health domain (i.e. not NCDs) |
| O'Boyle 2022 | No full-text |
| O'Connor 2021 | No effectiveness indices reported (e.g. only frequency of BCTs) |
| O'Reilly 2013 | No BCTs/process variables/intervention components/active elements reported |
| Oh 2021 | No BCTs/process variables/intervention components/active elements reported |
| Orr 2015 | No BCTs/process variables/intervention components/active elements reported |
| Ouedraogo 2022 | No effectiveness indices reported (e.g. only frequency of BCTs) |
| Patterson 2021 | No BCTs/process variables/intervention components/active elements reported |
| Peiris 2014 | No BCTs/process variables/intervention components/active elements reported |
| PƒÉsƒÉrelu 2017 | No BCTs/process variables/intervention components/active elements reported |
| Poorman 2015 | No BCTs/process variables/intervention components/active elements reported |
| Pugatch 2018 | No effectiveness indices reported (e.g. only frequency of BCTs) |
| Richards 2018 | No BCTs/process variables/intervention components/active elements reported |
| Robinson 2021 | No BCTs/process variables/intervention components/active elements reported |
| Rosser 2009 | No effectiveness indices reported (e.g. only frequency of BCTs) |
| Rossi 2017 | No effectiveness indices reported (e.g. only frequency of BCTs) |
| Russell 2018 | Wrong health domain (i.e. not NCDs) |
| Sawesi 2016 | No BCTs/process variables/intervention components/active elements reported |
| Schembre 2018 | No effectiveness indices reported (e.g. only frequency of BCTs) |
| Schultchen 2021 | No effectiveness indices reported (e.g. only frequency of BCTs) |
| Sediva 2022 | No effectiveness indices reported (e.g. only frequency of BCTs) |
| Sezgin 2022 | No BCTs/process variables/intervention components/active elements reported |
| Shams 2021 | No effectiveness indices reported (e.g. only frequency of BCTs) |
| Sharpe 2017 | No effectiveness indices reported (e.g. only frequency of BCTs) |
| Simon 2021 | No BCTs/process variables/intervention components/active elements reported |
| Sin 2020 | Wrong health domain (i.e. not NCDs) |
| Slater 2017 | No effectiveness indices reported (e.g. only frequency of BCTs) |
| Sotirova 2021 | Wrong focus (i.e. App design/development, content analyses, user satisfaction, technical details) |
| Spaulding 2021 | No BCTs/process variables/intervention components/active elements reported |
| Steinkamp 2019 | No effectiveness indices reported (e.g. only frequency of BCTs) |
| Steinkamp 2019 | No effectiveness indices reported (e.g. only frequency of BCTs) |
| Steubl 2021 | Wrong health domain (i.e. not NCDs) |
| Stevenson 2019 | Wrong health domain (i.e. not NCDs) |
| Szinay 2020 | No effectiveness indices reported (e.g. only frequency of BCTs) |
| Teepe 2021 | No effectiveness indices reported (e.g. only frequency of BCTs) |
| Thomas 2021 | No effectiveness indices reported (e.g. only frequency of BCTs) |
| Tighe 2020 | No BCTs/process variables/intervention components/active elements reported |
| Tofighi 2017 | No effectiveness indices reported (e.g. only frequency of BCTs) |
| Tonkin 2017 | No effectiveness indices reported (e.g. only frequency of BCTs) |
| Usman 2022 | No BCTs/process variables/intervention components/active elements reported |
| Versluis 2016 | No BCTs/process variables/intervention components/active elements reported |
| Vugts 2018 | Wrong health domain (i.e. not NCDs) |
| Wang 2022 | No BCTs/process variables/intervention components/active elements reported |
| Williamson 2022 | No effectiveness indices reported (e.g. only frequency of BCTs) |
| Xie 2022 | No BCTs/process variables/intervention components/active elements reported |
| Yang 2021 | No effectiveness indices reported (e.g. only frequency of BCTs) |
| Yang 2022 | No BCTs/process variables/intervention components/active elements reported |
| Yogeswaran 2021 | Wrong health domain (i.e. not NCDs) |
| Zhang 2021 | No BCTs/process variables/intervention components/active elements reported |
| Zhang 2022 | No BCTs/process variables/intervention components/active elements reported |

Notes: Ten duplicates that were manually detected are not reported since these are already included in the table or were included in the review. Studies that obviously did not fulfil the inclusion criteria for the review as listed under ‘Types of studies’, ‘Types of articles’, ‘Types of participants’, and ‘Types of interventions’ are not listed.
