# Supplementary material for: Effective Behavior Change Techniques in Digital Health Interventions for the Prevention or Management of Noncommunicable Diseases: An Umbrella Review
Source: Ann Behav Med. 2023 Aug 25;57(10):817–35. doi: 10.1093/abm/kaad041 (PMC10498822; doi:10.1093/abm/kaad041)
Supplement: kaad041_suppl_Supplementary_File_4 [file kaad041_suppl_supplementary_file_4.docx]

Supplementary File 4. AMSTAR 2 Assessment

| Author | Item |  |  |  |  |  |  |  |  |  |  |  |  |  |  |  | Confidence Rating |
| --- | --- | --- | --- | --- | --- | --- | --- | --- | --- | --- | --- | --- | --- | --- | --- | --- | --- |
|  | 1 | 2 | 3 | 4 | 5 | 6 | 7 | 8 | 9 | 10 | 11 | 12 | 13 | 14 | 15 | 16 |  |
| Aalbers 2011 | Y | N | Y | N | Y | Y | N | PY | PY | N | NA | NA | Y | N | NA | N | Critically Low |
| Akinosun 2021 | Y | PY | N | PY | Y | Y | Y | PY | Y | N | Y | N | N | N | PY | Y | Low |
| Antoun 2022 | N | N | N | PY | Y | Y | Y | PY | Y | N | Y | Y | Y | Y | Y | Y | Low |
| Armitage 2020 | Y | PY | N | PY | Y | Y | N | PY | Y | N | Y | Y | Y | Y | PY | Y | Moderate |
| Athilingam 2018 | Y | N | N | PY | N | N | N | N | PY | N | NA | NA | Y | Y | NA | Y | Low |
| Besson 2020 | Y | N | N | PY | Y | Y | N | PY | N | N | NA | NA | N | N | NA | N | Critically Low |
| Bond 2021 | Y | N | N | PY | N | N | N | PY | PY | N | NA | NA | Y | Y | NA | Y | Low |
| Buckingham 2019 | Y | PY | N | PY | Y | Y | N | PY | PY | N | NA | NA | Y | Y | NA | Y | Moderate |
| Chen 2020 | Y | PY | N | PY | N | Y | N | PY | PY | N | NA | NA | Y | N | NA | Y | Moderate |
| Connelly 2013 | Y | N | Y | PY | N | N | N | PY | N | N | NA | NA | N | N | NA | Y | Critically Low |
| Cucciniello 2021 | Y | N | Y | Y | Y | N | N | PY | PY | N | NA | NA | Y | N | NA | Y | L ow |
| Dao 2021 | Y | PY | N | PY | Y | Y | Y | PY | Y | N | NA | NA | N | N | NA | Y | Low |
| Davies 2012 | Y | N | Y | PY | N | Y | N | PY | PY | N | Y | Y | Y | Y | Y | Y | Low |
| Davis 2020 | Y | PY | N | PY | Y | Y | N | PY | PY | N | NA | NA | Y | N | NA | Y | Moderate |
| de Leeuwerk 2022 | Y | PY | N | PY | Y | Y | N | PY | Y | N | Y | Y | Y | Y | PY | Y | Moderate |
| Dening 2020 | Y | PY | N | PY | Y | N | N | PY | PY | N | NA | NA | N | Y | NA | Y | Low |
| Direito 2017 | Y | PY | Y | PY | Y | Y | N | PY | Y | N | Y | Y | N | Y | PY | Y | Low |
| Donovan 2022 | Y | PY | N | Y | Y | Y | N | PY | N | N | NA | NA | N | N | NA | Y | Critically Low |
| Duff 2017 | Y | N | Y | PY | Y | Y | N | PY | Y | N | NA | NA | N | N | NA | Y | Critically Low |
| Dugas 2020 | Y | N | N | N | N | Y | N | PY | N | N | NA | NA | N | N | NA | Y | Critically Low |
| El-Gayar 2021 | Y | N | N | PY | Y | N | N | PY | Y | N | Y | Y | Y | Y | Y | Y | Low |
| Ester 2022 | N | PY | Y | PY | Y | Y | N | PY | Y | N | NA | NA | Y | Y | NA | Y | Moderate |
| Fanning 2012 | N | N | N | PY | Y | Y | N | N | N | N | Y | N | N | Y | N | Y | Critically Low |
| Free 2013 | Y | PY | N | Y | Y | Y | Y | Y | Y | N | Y | Y | Y | Y | Y | Y | Moderate |
| Fu 2017 | Y | N | N | PY | N | N | N | Y | Y | N | NA | NA | N | N | NA | Y | Critically :Low |
| Furness 2020 | Y | PY | N | PY | Y | Y | N | PY | Y | N | Y | N | Y | N | N | Y | Low |
| Getty 2019 | N | PY | N | PY | Y | N | N | N | Y | N | N | N | N | N | Y | Y | Critically Low |
| Gitonga 2022 | N | PY | N | PY | Y | Y | N | Y | Y | N | Y | N | N | Y | Y | Y | Low |
| Griffiths 2018 | Y | PY | N | PY | Y | Y | N | N | Y | N | Y | Y | Y | Y | Y | Y | Moderate |
| Hadjiconstantinou 2016 | Y | PY | N | PY | Y | Y | N | PY | PY | N | Y | N | Y | Y | Y | Y | Moderate |
| Hardeman 2019 | Y | PY | N | Y | Y | Y | N | Y | N | N | NA | NA | N | N | NA | Y | Critically Low |
| Harris 2011 | N | PY | Y | Y | Y | Y | Y | PY | Y | Y | Y | Y | Y | Y | Y | Y | High |
| Hohberg 2022 | Y | PY | N | PY | Y | Y | N | PY | Y | N | NA | NA | Y | Y | NA | Y | Moderate |
| Howlett 2022 | Y | PY | N | PY | Y | Y | N | PY | Y | Y | NA | NA | Y | Y | NA | Y | Moderate |
| Humphreys 2021 | N | PY | N | Y | Y | N | N | PY | Y | N | NA | NA | Y | N | NA | Y | Moderate |
| Kanejima 2019 | Y | N | N | N | N | N | N | PY | Y | N | Y | N | Y | Y | N | Y | Critically Low |
| Kaner 2017 | Y | Y | N | Y | Y | Y | Y | Y | Y | Y | Y | Y | Y | Y | Y | Y | High |
| Khoong 2021 | N | PY | N | Y | N | Y | N | Y | N | N | N | N | N | N | N | Y | Critically Low |
| Kim 2021 | Y | N | N | PY | Y | Y | N | PY | Y | N | Y | Y | Y | Y | Y | Y | Low |
| Knowlden 2022 | Y | Y | N | PY | Y | Y | N | PY | PY | Y | NA | NA | Y | N | NA | Y | Moderate |
| Laranjo 2020 | Y | Y | N | PY | Y | N | PY | PY | Y | Y | Y | Y | Y | Y | Y | Y | Moderate |
| Lehto 2011 | N | N | N | Y | N | N | Y | N | N | N | NA | NA | N | N | NA | Y | Critical Low |
| Levine 2015 | N | PY | N | PY | Y | Y | N | PY | Y | N | NA | NA | Y | N | NA | Y | Moderate |
| Li 2020 | Y | PY | N | PY | Y | Y | N | PY | Y | Y | Y | Y | Y | Y | Y | Y | Moderate |
| Liu 2013 | Y | N | N | PY | N | Y | N | PY | N | N | Y | N | N | N | Y | Y | Critically Low |
| Liu 2020 | Y | N | Y | PY | Y | Y | N | PY | Y | N | Y | N | N | Y | Y | Y | Critically Low |
| Luo 2021 | Y | PY | Y | PY | Y | Y | Y | PY | Y | N | NA | NA | N | N | NA | Y | Low |
| Lyzwinski 2014 | Y | N | N | PY | N | N | N | PY | Y | N | N | N | Y | N | Y | Y | Critically Low |
| McCrabb 2019 | Y | PY | N | PY | Y | Y | N | PY | Y | N | Y | N | N | N | Y | Y | Low |
| McMahon 2020 | Y | PY | N | PY | Y | Y | N | PY | Y | N | Y | Y | Y | Y | N | Y | Low |
| Miller 2017 | Y | N | N | PY | N | N | Y | PY | Y | N | Y | Y | Y | Y | Y | Y | Low |
| Milne-Ives 2020 | Y | PY | Y | PY | Y | Y | N | N | Y | N | NA | NA | Y | N | NA | Y | Moderate |
| Muellmann 2018 | Y | PY | N | PY | Y | Y | N | Y | Y | N | NA | NA | N | N | NA | Y | Low |
| Murimi 2019 | Y | N | N | PY | Y | Y | N | PY | N | N | NA | NA | Y | N | NA | Y | Critically Low |
| Newby 2020 | Y | Y | Y | PY | Y | N | N | PY | Y | N | Y | Y | Y | N | N | Y | Low |
| Palacios 2017 | Y | N | N | PY | Y | Y | N | PY | Y | N | NA | NA | N | Y | NA | Y | Critically Low |
| Pal 2013 | Y | Y | N | PY | Y | Y | Y | Y | Y | Y | Y | Y | Y | Y | Y | Y | High |
| Palmer 2018 | Y | Y | Y | PY | Y | Y | Y | Y | Y | Y | NA | NA | Y | Y | NA | Y | High |
| Patterson 2022 | N | PY | N | PY | N | Y | N | Y | Y | N | Y | NA | N | N | NA | Y | Low |
| Perski 2022 | Y | PY | Y | PY | Y | Y | N | N | N | N | NA | NA | N | Y | NA | Y | Critically Low |
| Pfaelffli Dale 2015 | Y | N | N | PY | Y | Y | Y | Y | Y | N | NA | NA | Y | N | NA | Y | Low |
| Pouls 2021 | Y | PY | N | PY | Y | Y | N | PY | Y | N | NA | NA | Y | N | NA | Y | Moderate |
| Qan'ir 2019 | N | N | Y | PY | Y | N | N | PY | Y | N | NA | NA | Y | Y | NA | Y | Low |
| Rhodes 2020 | Y | PY | N | PY | Y | Y | N | PY | Y | N | Y | N | N | N | N | Y | Critically Low |
| Roberts 2017 | N | PY | Y | PY | Y | Y | N | Y | Y | N | Y | N | Y | Y | Y | Y | Moderate |
| Rodriguez Rocha 2019 | Y | N | Y | PY | N | Y | N | PY | Y | N | Y | Y | N | Y | Y | Y | Critically Low |
| Schoeppe 2016 | N | N | N | PY | Y | Y | N | Y | N | N | NA | NA | N | N | NA | Y | Critically Low |
| Seo 2015 | N | N | N | PY | Y | N | N | PY | Y | N | Y | N | N | Y | Y | Y | Critically Low |
| Sherrington 2016 | Y | PY | N | PY | Y | N | N | N | Y | N | Y | N | N | Y | N | Y | Critically Low |
| Staiger 2020 | N | N | N | PY | Y | N | N | Y | Y | N | NA | NA | Y | N | NA | Y | Low |
| Stephenson 2017 | Y | PY | N | PY | N | N | N | PY | Y | N | Y | N | Y | Y | N | Y | Low |
| Taylor 2022 | N | N | N | PY | Y | N | N | Y | Y | N | NA | NA | N | N | NA | Y | Low |
| Thomas Craig 2020 | Y | PY | N | PY | Y | Y | N | PY | N | N | NA | NA | N | Y | NA | Y | Critically Low |
| Tong 2021 | Y | PY | N | PY | Y | N | Y | PY | Y | Y | Y | Y | Y | Y | Y | Y | Moderate |
| Tong 2018 | N | Y | Y | PY | Y | Y | Y | PY | Y | Y | Y | N | N | N | N | Y | Critically Low |
| Triantafyllidis 2018 | Y | N | N | PY | Y | N | N | PY | Y | N | NA | NA | N | N | NA | Y | Critically Low |
| VanVugt 2013 | N | N | Y | PY | Y | Y | N | Y | PY | N | NA | NA | N | N | NA | Y | Critically Low |
| Vetrovsky 2021 | Y | Y | Y | PY | Y | Y | N | Y | Y | N | Y | Y | Y | Y | Y | Y | Moderate |
| Villinger 2019 | N | N | N | PY | Y | Y | Y | PY | Y | Y | Y | Y | Y | Y | Y | Y | Low |
| Webb 2010 | N | N | N | PY | N | N | N | N | N | N | Y | N | N | Y | N | N | Critically Low |
| Western 2021 | Y | PY | Y | PY | Y | Y | N | Y | Y | N | Y | Y | Y | Y | Y | Y | Moderate |
| Willmott 2019 | N | N | N | PY | Y | Y | N | PY | Y | N | NA | NA | Y | N | NA | Y | Low |
| Wright 2021 | N | PY | N | PY | Y | Y | N | PY | Y | Y | NA | NA | N | N | NA | Y | Low |
| Xu 2022 | N | N | N | PY | Y | Y | N | PY | Y | N | NA | NA | Y | Y | NA | Y | Low |
| Yang 2020 | N | N | N | PY | Y | Y | N | N | N | N | Y | N | N | N | Y | N | Critically Low |

Notes: Critical domains are highlighted by grey columns. In this review, item 7 (justification for excluding individual studies) was considered a non-critical weakness if individual excluded studies were not listed but a PRISMA flowchart was present that provided a summary of excluded studies and reasons for exclusion. If individual excluded studies were not listed or excluded studies were not summarised in a PRISMA flow chart then this was regarded a critical flaw.

Y = Yes; PY = partial yes; N=No; NA= not applicable
